# Improvements to seismicity forecasting based on a Bayesian spatio-temporal ETAS model

**DOI:** 10.1038/s41598-022-24080-1

**Published:** 2022-12-05

**Authors:** Hossein Ebrahimian, Fatemeh Jalayer, Behnam Maleki Asayesh, Sebastian Hainzl, Hamid Zafarani

**Affiliations:** 1 Department of Structures for Engineering and Architecture, University of Naples Federico II, Naples, Italy; 2 Institute for Risk and Disaster Reduction (IRDR), University College London, London, UK; 3 Institute of Geosciences, University of Potsdam, Postdam, Germany; 4 GFZ German Research Center for Geosciences, Postdam, Germany; 5 International Institute of Earthquake Engineering and Seismology (IIEES), Tehran, Iran

**Keywords:** Natural hazards, Statistics, Seismology

## Abstract

The epidemic-type aftershock sequence (ETAS) model provides an effective tool for predicting the spatio-temporal evolution of aftershock clustering in short-term. Based on this model, a fully probabilistic procedure was previously proposed by the first two authors for providing spatio-temporal predictions of aftershock occurrence in a prescribed forecasting time interval. This procedure exploited the versatility of the Bayesian inference to adaptively update the forecasts based on the incoming information provided by the ongoing seismic sequence. In this work, this Bayesian procedure is improved: (1) the likelihood function for the sequence has been modified to properly consider the piecewise stationary integration of the seismicity rate; (2) the spatial integral of seismicity rate over the whole aftershock zone is calculated analytically; (3) background seismicity is explicitly considered within the forecasting procedure; (4) an adaptive Markov Chain Monte Carlo simulation procedure is adopted; (5) leveraging the stochastic sequences generated by the procedure in the forecasting interval, the N-test and the S-test are adopted to verify the forecasts. This framework is demonstrated and verified through retrospective early forecasting of seismicity associated with the 2017–2019 Kermanshah seismic sequence activities in western Iran in two distinct phases following the main events with Mw7.3 and Mw6.3, respectively.

## Introduction

Within the first days elapsed after the occurrence of an earthquake and in the presence of an ongoing seismic sequence, emergency decision-making can benefit enormously from the short-term operational seismicity forecasts^1–10^. The Epidemic Type Aftershock Sequence (ETAS) model is a widely used stochastic model to describe earthquake temporal^11^ and spatio-temporal^12^ occurrence and clustering of seismicity within a seismic sequence (see also^13–15^). It is an epidemic-type stochastic point process^16^ in which every earthquake within the sequence is a potential triggering event for subsequent earthquakes by generating its own aftershocks based on a Modified Omori (MO^17^) decay. According to the study by^18^, the ETAS model is the best model for describing short-term seismicity. The ETAS model performed quite well in retrospectively forecasting the seismicity within various operational frameworks in California, Greece, Italy, Japan, Spain, New Zealand, and Iceland^6,19–34^. It has been employed for time-dependent seismic hazard^8,25,33,35,36^, and risk and loss forecasting^8,35,37–40^.

Ogata^11^ employed the maximum likelihood (ML) criterion parameter estimation (which serves currently as the most well-established and widespread method) for the temporal ETAS model. The method was extended also for the spatio-temporal ETAS model^12–14^. Several attempts were made for developing improved algorithms to attain ML estimates of ETAS parameters^41,42^. Algorithms based on numerical optimization methods for ML estimation may not be very efficient for seismicity forecasting within an ongoing seismic sequence for the following reasons: (a) they are computationally demanding and may encounter convergence problems (especially when the log-likelihood function is extremely flat or multimodal); (b) some algorithm parameters need to be tuned; something that sounds difficult to do automatically. Thus, the model parameters are usually calibrated a priori. New algorithms based on Simulated Annealing optimization technique that allows for a more automatic ML estimation of model parameters were developed^43,44^; however, even this procedure is not totally autonomous and proper choice of tuning parameters is warranted. In case of providing early forecasts immediately after a large earthquake (e.g., using incomplete catalogues), ML estimation of the ETAS model parameters may include large estimation errors^45^. This may cause bias in ETAS parameters that arises from missing data^46,47^.

Bayesian parameter estimation coupled with efficient simulation procedures, such as Markov Chain Monte Carlo Simulation, has the advantage of adaptively tuning into the ongoing sequence. This procedure can provide both joint probability distributions for the ETAS model parameters and the predicted spatio-temporal seismicity evolution. This is particularly important for early forecasts based on incomplete datasets. Bayesian parameter estimation has been employed in different aftershock models (MO model and spatio-temporal MO model both for L’Aquila 2009 seismic sequence in Italy^24,37^; temporal ETAS model for L’Aquila 2009 seismic sequence^26^, for 38 aftershock sequences in Japan from 1990–2014^45^, for 2016 Kumamoto earthquake sequence in Japan^48^, for 2019 Ridgecrest California seismic sequence^49^; spatio-temporal ETAS model for 2010–2019 southern California dataset^50^). It is to note that ETAS-based seismicity rate forecasts can be improved by incorporating additional main shock information including the rupture geometry and the coulomb stress changes^51^ or other stress scalars such as maximum shear and von-mises stress^52^, which might be available within minutes to hours after the events.

Ebrahimian and Jalayer^28^ proposed a fully simulation-based framework for both Bayesian updating of spatio-temporal ETAS model parameters as well as a *robust* estimate of spatial distribution of events in a prescribed forecasting time interval after the main event. The procedure was applied for retrospective forecasting of early seismicity associated with the 2016 Amatrice seismic sequence in central Italy. The forecasting is robust (see^53,54^ for robust reliability assessment), since both the uncertainty in the ETAS model parameters and the uncertainty in the sequence of events that is going to occur during the forecasting interval is considered. This simulation-based framework consists of two parts: (a) Bayesian updating to learn the ETAS model parameters conditioned on the events that have already taken place (registered) in the ongoing seismic sequence. Markov Chain Monte Carlo (MCMC) simulation scheme^54^ is used to sample directly from the conditional posterior probability distribution for ETAS model parameters (see also^26,45,48^ for the use of MCMC in ETAS parameter estimation). (b) Adaptively generate plausible sequences of events during the forecasting interval. For a given forecasting interval, this leads to spatial distribution of the forecasted events and probability distribution of the number of events. The outcomes of this robust seismicity forecasting are directly applicable in adaptive daily aftershock hazard (e.g.,^24,33,55,56^) and risk assessment procedures (see e.g.,^35,37,39^).

This work strives to improve different aspects of the fully probabilistic seismicity forecasting framework proposed in^28^. These improvements are: (1) the likelihood function for Bayesian updating has been modified to adopt the piece-wise continuous formulation for a marked point process^57^, whereas the previous work assumed magnitude and interarrival time to be jointly Poissonian. (2) The spatial integral of the conditional seismicity rate over the aftershock zone is calculated analytically (previously it was assumed to be equal to unity). (3) An adaptive MCMC simulation procedure for Bayesian updating of the ETAS model parameters have been employed by using the concept of multi-dimensional kernel sampling density function (which decreases the computational time; previously we have considered a simple component-wise MCMC procedure). (4) The background seismicity has been incorporated within the robust seismicity forecasting framework (previously, we have considered the background seismicity as an added rate to the forecasted rate of seismicity). (5) The stochastic process for generating sequences, i.e. part (b) described above, within the forecasting interval has been adjusted to the updated likelihood function and to the exact integral over the areal extent of the aftershock zone. (6) Leveraging the stochastic sequences generated by the procedure in the forecasting interval, the N-test and the S-test (see^58,59^) are adopted to verify the forecasts. It is noted that this framework can automatically “tune-in” into the sequence of observed events and model updating, and forecasting are carried out without human interference and use of expert judgement. It is very efficient for early forecasts and in the presence of incomplete data. The refined framework is applied retrospectively to the Kermanshah seismic sequence 2017–2019 (see subsequent “The Kermanshah 2017–2019 seismic sequence” section) to provide early seismicity forecasting for two distinct phases of the sequence. The paper is organized as following: “The Kermanshah 2017–2019 seismic sequence” section provides an overview of the 2017–2019 Kermanshah seismic sequence. “Results” section shows the results of the seismicity forecasting for two important seismic sub-sequences including the Azgeleh mainshock (the main event with M_w_7.3 and its early triggered aftershocks, Phase 1), and the Sarpol-e Zahab event (the second main event with M_w_6.3 and its early triggered aftershocks, Phase 3). We have also investigated the sensitivity of the forecasts to some possible variations in the proposed workflow, which include: comparing two different spatial kernel density functions in the ETAS model; not considering the background seismicity; approximating the spatial integral of the conditional seismicity rate over the aftershock zone; and finally estimating the productivity coefficient of the ETAS model by two different approaches. “Discussion and conclusions” section is the general discussion and conclusions, and finally “Methods” section describes the proposed improved methodology for seismicity forecasting.

### The Kermanshah 2017–2019 seismic sequence

**Figure 1:**
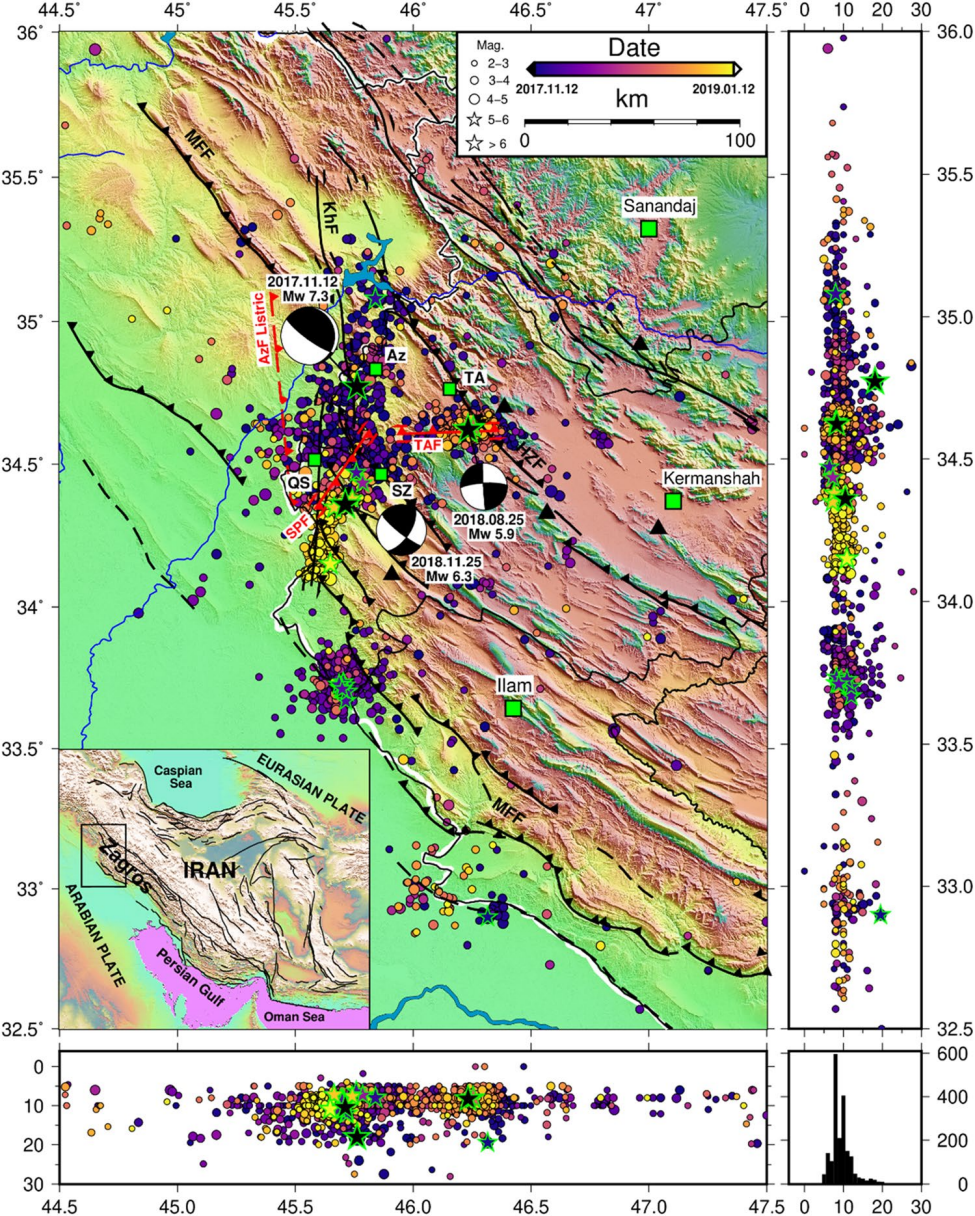
Main tectonic features of Iranian plateau; epicentral and depth distribution of the main events of Kermanshah seismic sequence 2017–2019 including Azgeleh, Tazehabad, and Sarpol-e Zahab earthquakes with black stars, and the distribution of seismic events (circles and green stars) with M_w_ ≥ 2.5 registered by IRSC from 12/11/2017 up to 12/01/2019. The green stars show the events with M_w_ ≥ 5. The color of stars and circles are based on the evolution of events. The black bitch balls show the focal mechanism of main events (based on the observations in^64^). The big green squares show the main cities and small squares illustrate the small cities and towns nearby the epicentral area of the mains events (Az: Azgeleh, TA: Tazehabad, SZ: Sarpol-e Zahab, and QS: Qasr-e Shirin). The red solid and dashed lines show the surface projection of causative faults of the main events (AzF Listric: Azgeleh Listric slip model, TAF: Tazehabad fault, and SPF: Sarpol –e Zahab fault). The black solid lines in the map and the inset map show the main active faults in the area and the Iranian plateau (MFF: Main Front Fault, HZF: High Zagros Fault, and KhF: Khanaqin Fault); black triangles are the IRSC stations. The histogram in the right lower part illustrates the depth distribution of the seismic events.

Iranian plateau is a seismically active region whose active tectonic is dominated by the convergence of Arabian and Eurasian plates (~ 20 to 30 mm/year, see the inset map in Fig. 1). Approximately one third of this overall convergence rate is accommodated by the “Zagros” range as a central part of Alpine-Himalayan orogenic belt in southwestern Iran (see the inset map in Fig. 1)^60^. The Zagros orogen is one of the youngest, and most tectonically active intracontinental belts in the world at the leading edge of the Arabian-Eurasian continental collision zone^61,62^. On 12 November 2017 (18:18 UTC), an earthquake with M_w_7.3 struck the Zagros fold-and-thrust belt (ZFTB) in the Western Iran near the Iraq border in the area where large earthquakes had not been documented for several centuries. This severe earthquake occurred near the two small cities of Azgeleh and Sarpol-e Zahab (Az and SZ, see Fig. 1). This event which is called herein “Azgeleh” main shock (Azgeleh MS) inflicted around 630 casualties and caused immense buildings’ damages and economic losses^63^. Based on the comprehensive study performed in^64^, this event is an oblique-slip thrust with a considerable dextral component (Strike 354°, Dip 16°, and Rake 137°). They proposed a listric fault geometry for characterizing the Azgeleh MS (see the corresponding fault “AzF” Listric in Fig. 1), with a shallow angle plane (eastern plane) dipping ~ 3°, a ramp dipping ~ 16° (central plane), and a steeper ramp dipping 25° (western plane). This MS triggered intense seismicity within the aftershock zone shown in Fig. 1 (the position of this area within the map of Iran is shown in the inset). From 12/11/2017 up to 18/04/2020 (i.e., in the time interval of around 2.5 years after the Azgeleh MS), about 9000 seismic events were recorded based on the catalog of Iranian Seismological Center (IRSC) issued by the Institute of Geophysics of the University of Tehran (IGUT seismic network) in the area shown in Fig. 1. From this pool of seismicity, 2318 events have M_w_ ≥ 2.5. In addition to the Azgeleh MS, 19 events with M_w_ ≥ 5.0 (one event, which will be discussed later, has M_w_ ≥ 6.0), and more than 125 events with magnitude larger than 4 and less than 5 (4 ≤ M_w_ < 5) were recorded within this sequence. Since this sequence has initiated in the Kermanshah province of Iran, it is called herein “*Kermanshah seismic sequence*”. Figure 1 illustrates Kermanshah seismic sequence 2017–2019 in the time span of 12/11/2017 up to 12/01/2019, where events with M_w_ ≥ 2.5 are colored according to their occurrence time. The data is extracted from IRSC catalog issued by IGUT. The M_w_7.3 Azgeleh MS initiated “Phase 1” of Kermanshah seismic sequence. About 9 months after Azgeleh MS, on 25 August 2018 (22:13 UTC), an earthquake with M_w_5.9 occurred 45 km to the east of the epicenter of Azgeleh MS, close to the town Tazehabad (TA, see Fig. 1) with three casualties. At first glance, this new event was considered as an aftershock of the Azgeleh MS or a triggered event on the known faults of the study area (i.e., HZF or MFF faults shown in Fig. 1); however, reported focal mechanism from national and international seismological agencies and interferometric observation revealed that this event was associated to a previously unknown fault. The non-linear and linear inversion of the down-sampled data by^64^ showed a triggered east–west trending fault (Strike 267°, Dip 78°, and Rake 2°) with dip to the north for this event. The event is called “Tazehabad” earthquake, and the corresponding left-lateral strike slip fault is called “TAF” in Fig. 1). The M_w_5.9 Tazehabad earthquake initiated “Phase 2” of this seismic sequence. About 3 months after the Tazehabab event and one year after the Azgeleh MS, on 25 November 2018 (16:37 UTC) another earthquake with M_w_6.3 struck near the Sarpol-e Zahab and Qasr-e Shirin cities (SZ and QS in Fig. 1). This event is called “Sarpol-e Zahab” earthquake, which started “Phase 3” of the Kermanshah seismic sequence. Fathian et al.^64^ showed that the best-fitting inversion on the coseismic surface deformations of the Sarpol-e Zahab event agrees well with a single fault plane with a NE trend dipping to the southeast (Strike 34°, Dip 63°, and Rake 170°, see right-lateral strike slip fault “SPF” in Fig. 1). As a result of the characterized main events, Kermanshah seismic sequence 2017–2019 is composed of three main sub-sequences. These sub-sequences can be seen within the evolution of seismic activities in Fig. 1, where the events are colored according to their occurrence time.

## Results

### Daily forecasts of seismicity for the first days after the event Mw7.3 of 12/11/2017 (Phase 1)

**Figure 2:**
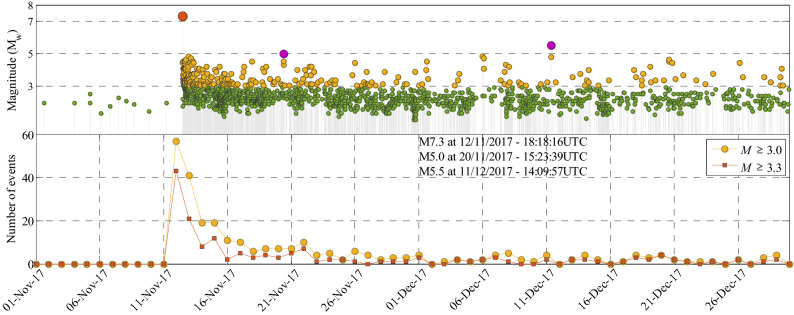
Magnitude vs. time (top) and the number of events with *M* ≥ 3.0 and *M* ≥ 3.3 (bottom) occurred within a 24-h interval starting from 6:00 UTC of each day from 01/11/2017 (*T*_o_) until 30/12/1017 including the Azgeleh MS of M_w_7.3 within phase 1 of the complex Kermanshah 2017–2019 seismic sequence.

In this section, we strive to perform robust forecasts for the spatio-temporal evolution of the events in specific time intervals within phase 1 of the complex seismic sequence started by the Azgeleh MS of M_w_7.3, which took place in November 12, 2017, at 18:18 (UTC). Figure 2 (top) shows the evolution of events within the catalog characterized by their magnitude within the aftershock (AS) zone in phase 1 starting from 01/11/2017 up to 30/12/2017. The AS zone (**A**, see “The epidemic-type aftershock sequence (ETAS) model for space–time clustering of aftershocks” section in “Methods”) is selected to be smaller than the whole zone shown in Fig. 1 in the latitudinal range of [32.50–35.50] and longitudinal range of [45–47]. Figure 2 (bottom) shows the evolution of the number of events within the AS zone in phase 1 with magnitude *M* ≥ 3.0 (orange circles) and *M* ≥ 3.3 (red squares) within a 24-h interval starting from 6:00 UTC each day (also reported at 6:00 UTC each day). The time origin *T*_o_ (the reference time) is set to 6:00 UTC of 01/11/2017 (i.e., 11 days before the main event). The seismicity after the occurrence of the mainshock increases considerably up to 15 November (afterwards, less than 20 events with *M* ≥ 3.0 within a day have occurred). The occurrence of two triggered events of M_w_5.0 at 20/11/2017 and M_w_5.5 at 11/12/2017 (with very close epicenters) did not significantly increase the seismicity of the site. We examine the proposed improved ETAS framework for the following time windows [*T*_*start*_, *T*_*end*_]: (a) [12/11/2017–21:00UTC, 13/11/2017–06:00UTC] (i.e., 9 h forecasting with *T*_*start*_ at 2 h and 42 min after the main event); (b) [13/11/2017–00:00UTC, 13/11/2017–06:00UTC] (i.e., 6 h forecasting with *T*_*start*_ at 5 h and 42 min after the main event); (c) [13/11/2017–06:00UTC, 14/11/2017–06:00UTC]; (d) [14/11/2017–06:00UTC, 15/11/2017–06:00UTC]; (e) [15/11/2017–06:00UTC, 16/11/2017–06:00UTC]. The latter three are one-day (24 h) forecasting intervals issued at 06:00 UTC each day. For each forecasting time window [*T*_*start*_, *T*_*end*_], the observation history, **seq**, comprises all the events in the interval [*T*_o_, *T*_*start*_] with magnitude greater than lower magnitude (${M}_{l}$), set herein to be equal to the completeness magnitude ${M}_{c}$ (i.e., ${M}_{l}={M}_{c}$).

#### Estimating the magnitude of completeness ${{\varvec{M}}}_{{\varvec{c}}}$ for each forecasting interval

**Figure 3:**
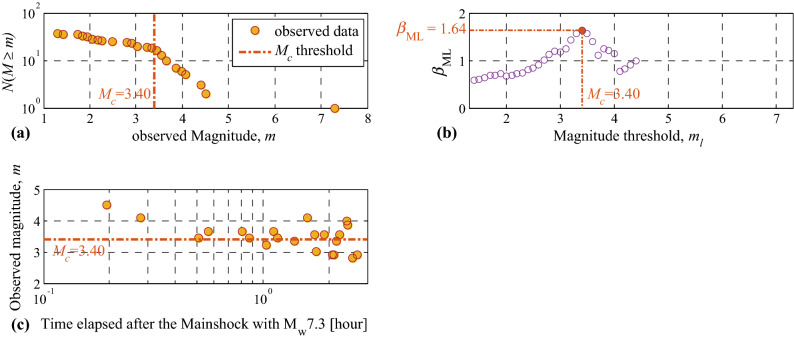
The three methods (Section SI-3-Results) for estimation/validation of ${M}_{c}$ for the first forecasting interval [12/11/2017–21:00UTC, 13/11/2017–06:00UTC] in phase 1 of the seismic sequence.

${M}_{c}$ Is estimated for each of the five forecasting intervals (see Supplementary Information Section SI-3-Results for description of three different strategies for estimation of ${M}_{c}$). Figure 3 illustrates the three ${M}_{c}$ estimation procedures for the first forecasting interval (a) described above. Figure 3a shows a frequency-magnitude semi-logarithmic plot, which shows a value of ${M}_{c}=3.40$ (the first method introduced in Section SI-3-Results). Figure 3b illustrates the mode (i.e. posterior maximum likelihood estimate) of the posterior probability distribution ${\beta }_{\mathrm{ML}}$ estimated through Bayesian inference as a function of magnitude thresholds ${m}_{l}$ (the second method introduced in Section SI-3-Results). It shows that the maximum value ${\beta }_{\mathrm{ML}}=1.64$ leads to ${M}_{c}=3.40$. Figure 3c (the third method discussed in SI-3-Results) is merely a visual check to see if the observation history **seq** is complete at the time of issuing the forecast by setting ${M}_{c}=3.40$ (i.e., 2 h and 42 min elapsed after the main event). ${M}_{c}$ estimations based on the three methods for time intervals (b) to (e) are illustrated in Section SI-3-Results. In summary, we set ${M}_{c}=3.40$ for the first (early) forecasting interval (a), ${M}_{c}=3.30$ for the two subsequent forecasting intervals (b, and c), and ${M}_{c}=3.0$ for the ultimate two forecasting intervals (d, and e). Based on the background seismicity data provided in *Supplementary Information* (Section SI-4-Results, Figure SI-5b), the long-term background seismicity slope *β* for $M\ge 4.50$ is equal to $1.67$.

#### Bayesian Inference for **θ**

The first step towards providing seismicity forecasts (Eq. 9) is sampling from the distribution of model parameters **θ** based on posterior (target) probability distribution *p*(**θ**|**seq**,*M*_*l*_) (“Sampling **θ** from the distribution p(**θ**|**seq**, M_l_)” section in “Methods”). The vector **θ** = [*β*, *α*, *c*, *p*, *d*, *q*, *γ*], considering magnitude-dependent spatial kernel density (see “The epidemic-type aftershock sequence (ETAS) model for space–time clustering of aftershocks” section in “Methods”) is updated for each forecasting interval mentioned before based on the data provided (observed) within **seq** by applying the Bayesian updating routine illustrated in Eq. (18). It is considered that parameters *K* (Eq. 17), *K*_*t*_ (Eq. 3 as a function of *c* and *p*), *K*_*r*_ (Eq. 5 as a function of *d*, *q* and *γ*) are derived as functions of other parameters within vector **θ**. It is to mention that *K* is calculated as described in “Calculating K” section using Eq. (17) and is not learned directly through the Bayesian updating. Samples for **θ** are generated using an adaptive MCMC procedure from *p*(**θ**|**seq**,*M*_*l*_), as noted in “Sampling **θ** from the distribution p(**θ**|**seq**, M_l_))” section (see also Section SI-1-Method) with 6 chains (simulation levels). *p* and *q* values equal to or smaller than one are rejected according to Eqs. (3) and (4) and the constraints are *p* > 1 (making sure that the temporal process is asymptotically described by a long-term Gutenberg Richter seismicity model) and *q* > 1 (making sure that the spatial kernel integral is asymptotically equal to unity).

The first simulation level, for which a component-wise updating approach is employed, contains 520 original seeds (*Nseed* = 520). To bypass the initial transient effect of the Markov chain, the first 20 samples are discarded and *Nseed* = 500 is used for the first simulation level. For the next simulation levels (i.e., chains 2 to 6) we have employed the adaptive kernel estimate (Section SI-1-Method) ) with *Nseed* = 1000, by performing MCMC updating in a block-wise manner. The *N*_*d*_ ≤ *Nseed* shows the number of distinct Markov chain samples generated within the last (the 6th) simulation level. The background seismicity is also considered within the likelihood estimation as addressed in “Calculating the likelihood of the observed sequence p(seq|***θ***, M_l_)” section (according to background seismicity data provided in Section SI-4-Results). Table 1 illustrates the histograms of marginal posterior PDFs for the model parameters **θ** = [*β*, *α*, *c*, *p*, *d*, *q*, *γ*] (shown with bar plots) together with their prior PDFs (illustrated with dashed-orange lines); in addition, the histogram of parameter *K* derived based on Eq. (17) is shown in the last column. The statistics of the samples including mean and [2%-98%] confidence interval (CI) of the posterior of model parameters {**θ** = [*β*, *α*, *c*, *p*, *d*, *q*, *γ*], *K*} are also shown on the corresponding marginal distribution in Table 1. The last row of Table 1 reports the statistics for the prior marginal PDFs of **θ**. To capture the outliers of *K*, we have set the upper limit of the horizontal axis to be around 98th percentile of *K*. Since all ETAS parameters are positive, we assigned a multivariate lognormal distribution as prior to the model parameters **θ** (see Eq. 19; zero correlations are considered at the prior level) with a coefficient of variation (COV) equal to 0.5 for all the parameters (high COV is assumed to avoid using an over-informative prior distribution). The prior median values for both $\beta$ and $\alpha$ are set to $1.0\times \mathrm{ln}10=2.3026$. This selection was done deliberately to allow the Bayesian updating algorithm to infer these two parameters. It can be observed in Table 1 that $\alpha <\beta$ holds for all generated samples based on MCMC simulation. This is interesting investigation as we did not put any restriction on these two parameters while performing the Bayesian updating ($\alpha <\beta$ is a necessary condition for a stable “subcritical” process^65^). Prior median value $c={10}^{-1.53}\approx 0.03$ (day) is set based on the value provided by the MO Italian generic model parameters^66^, which is close to the California generic model (= 0.05; see^67,68^), and equal to the New Zealand generic model (= 0.03; see^69^). Prior median values assigned to [*p*, *d*, *q*] are equal to [1.1, 1.0, 1.5], which were used in^28^. The prior median for $\gamma =0.20$ is selected based on^70^. With reference to Table 1, the mean value of parameter $\beta$ matches quite well the values obtained based on ${\beta }_{\mathrm{ML}}$ (see Fig. 3 for the first forecasting interval and also figures in Section SI-3-Results for the subsequent forecasting intervals). The mean values of Parameter $\alpha \cong 1.05-1.12$ are quite invariant as the sequence evolves. The mean value of parameter *c* $\cong$ 0.05 (day) seems to be invariable in the first few days after the main event, which is reasonable as it measures the time offset. Parameters *p* and *d* gradually decrease as the observed data in **seq** accumulates, while *q* increases. Finally, the mean of the magnitude-dependent coefficient of the kernel density $\gamma \cong 0.31-0.39$. It is to note that as the sequence evolves and observed data in **seq** accumulates, the variation in the posterior of model parameters reduces. The temporal evolution of ETAS model parameters for both phases 1 and 3 of the seismic sequence is shown in Fig. 8 and will be discussed later in “Daily forecasts of seismicity after the event Mw6.3 of 25/11/2018 (Phase 3)” section.

**Table 1:**
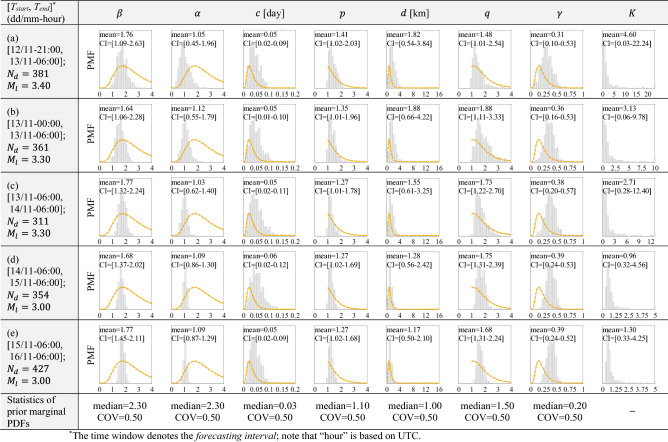
Distribution of the ETAS model parameters including marginal PDFs of posterior (shown as histograms) and lognormal PDFs of prior (shown with dashed orange lines), and the statistics of posterior (including mean and [2%-98%] confidence interval (CI) for providing early seismicity forecasts after M_w_7.3 at 12-November 2017.

##### Discussion on the correlations between pairs of model parameters **θ** in Phase 1

**Figure 4:**
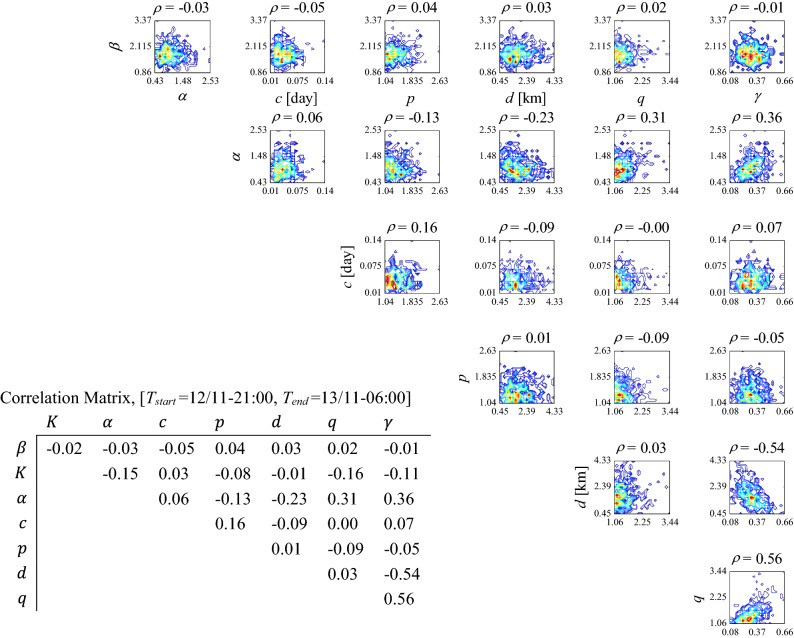
Contour lines representing the correlation between pairs of the posterior marginal samples of **θ** = [*β*, *α*, *c*, *p*, *d*, *q*, *γ*] for the first forecasting interval (1st row in Table 1) and the corresponding correlation matrix.

Figure 4 illustrates the contour lines showing the correlation structure between each of the sample marginal posteriors [*β*, *α*, *c*, *p*, *d*, *q*, *γ*] shown in the first row of Table 1 for the first forecasting interval (a). The correlation coefficient *ρ* is reported above each figure. We have reported the complete correlation matrix of the ETAS parameters **θ** = [ *β*, *K*, *α*, *c*, *p*, *d*, *q*, *γ*] in the left side of the contour plots in Fig. 4. It is revealed that *β* has almost no correlation or small correlation (up to the order of around 10%) with other ETAS parameters, as expected from the functional form of the seismicity rate *λ*_ETAS_ in Eqs. (1) and (2). Parameter *K* reveals small correlations with all ETAS parameters. Indeed, *K* is related to other ETAS parameters through Eq. (17). Parameter *α* shows correlation with temporal parameters (*c* and *p*) and even more significant correlation with the parameters of the spatial kernel density (*d*, *q*, *γ*). There is correlation between the temporal parameters (*c*, *p*), significant negative correlation between the spatial parameters (*d*, *γ*), and significant positive correlation between (*q*, *γ*). However, parameters (*d*, *q*) show no dependence. The correlation structure for ETAS parameters for the subsequent forecasting intervals (b) to (e) are shown in *Supplementary Information* (Section SI-5-Results). As the sequence evolves, parameter *K* tends to show more correlation with *p*. The correlations between *α* and temporal/spatial parameters hold more-or-less at the same amount. On the same page, the absolute values of correlations between (*c*, *p*) and (*d*, *γ*) increase, while the correlation between (*q*, *γ*) decreases.

#### Representing the robust forecasting results

Given that **θ** and **seq** are known, sequences of events taking place during the forecasting interval, denoted as **seqg**, are generated according to “Generating sequences according to p(seqg|**θ**, **seq**, M_l_)” section (“Methods”). Based on the description provided in Section SI-4-Results, ${M}_{\mathrm{max}}=7.5$ is considered for generating **seqg** for all the forecasting intervals within Phase 1 (“Daily forecasts of seismicity for the first days after the event Mw7.3 of 12/11/2017 (Phase 1)” section) and Phase 3 (“Daily forecasts of seismicity after the event Mw6.3 of 25/11/2018 (Phase 3)” section). Based on samples generated for **θ** and **seqg** in Eq. (9), the robust estimate for the number of events with *M* ≥ *m* in a spatial cell units centered at (*x*,*y*) within the aftershock zone is obtained. This robust estimate is calculated as the expected number of events considering the uncertainties in the spatio-temporal distribution of the sequence of events.

**Figure 5:**
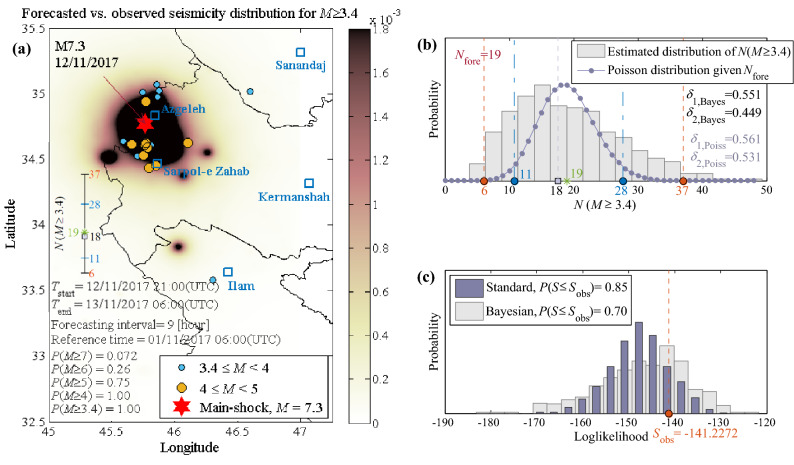
(**a**) The map showing forecasted vs. observed seismicity distribution in the aftershock zone for the 1st forecasting time window [*T*_*start*_ = 12/11/2017–21:00UTC, *T*_*end*_ = 13/11/2017–06:00UTC] including: the expected value for the number of events in each cell unit with *M* ≥ 3.40; the reported *P*(*M* ≥ *m*); the earthquakes that occurred during the corresponding forecasting time window; the mainshock of M_w_7.3; and bar chart showing the observed vs. the percentiles of the forecasted number of events. (**b**) N-test based on the simulation-based Bayesian workflow and the Poisson distribution; consistent with the error-bar on the left-side of (**a**), the green star shows the observed number of events within the forecasting interval (= 19), the grey-filled square is the median value or the 50th percentile (= 18); blue circles are the 16th and 84th percentiles (= 11 & 28); red circles show the 2nd and 98th percentiles (= 6 & 37); ${N}_{\text{fore}}=19$ is the expected number of events *N*(*M* ≥ 3.40). (c) S-test based on the simulation-based Bayesian framework and the standard method.

Figure 5a shows the forecasted seismicity maps for the number of events with *M* ≥ *M*_*l*_ = 3.40 issued for the first forecasting time window (a) in Phase 1 (as shown in Table 1) within each spatial cell unit (latitude/longitude cells of a 0.01° × 0.01° grid). The maps illustrate the expected number of events in each cell unit (${\mathbb{E}}[N(x, y, {M \ge M}_{l}|\mathbf{s}\mathbf{e}\mathbf{q}, {M}_{l})]$ in Eq. (9), Section “Methods”). The earthquakes of interest occurred within the corresponding forecasting interval, [*T*_*start*_, *T*_*end*_], are illustrated as colored dots (distinguished by their magnitudes) with the main event of M_w_7.3 (red star) illustrated on all plots. We also report the forecasted probabilities of having earthquakes with magnitude equal to or larger than *m* = [*M*_*l*_, 4, 5, 6, 7], denoted as *P*(*M* ≥ *m*), in the whole aftershock zone and in the corresponding forecasting interval (see Eq. 12). Inside Fig. 5a, the observed (shown as a green star) vs. forecasted number of events (shown in an error-bar format) is illustrated for events with *M* > *M*_*l*_ for the entire aftershock zone. The forecasted number of events on the error-bar chart features: the median value (the 50th percentile, equivalent to the exponential of the logarithmic mean) marked with a gray-filled square; the (logarithmic) mean ± one (logarithmic) standard deviation indicating the interval between 16 and 84th percentiles (marked with blue horizontal lines and numbered in blue); the (logarithmic) mean ± two (logarithmic) standard deviations indicating the interval between 2nd and 98th percentiles (marked with black horizontal lines and numbered in red). Obviously, we have rounded the forecast statistics to the nearest integers. This bar chart helps in locating the observed number of events (marked and numbered in green) within plus or minus certain number of standard deviations from the median estimate.

The N-test (see “N-test based on the simulation-based Bayesian framework” section in “Methods”) is used to validate the total number of events forecasted by the proposed method. Figure 5b shows the N-test associated with each forecasting interval. The robust estimate for the number of events within the forecasting interval using Eq. (9) will lead to the distribution of events with $M\ge {M}_{l}$, denoted as $N\left(M\ge {M}_{l}|{\varvec{\uptheta}},\mathbf{s}\mathbf{e}\mathbf{q}, {M}_{l}\right)$ in Eq. (12), as shown with the histogram in these figures. This distribution is shown also with error-bar chart in Fig. 5a. The observed number of events within the forecasting interval, ${N}_{\text{obs}}$, is shown as a green star (as also shown on the bar chart). For the N-test, we use the same colors as the bar chart to represent the forecasted number of events: the median value (the 50th percentile) is marked with a grey-filled square; the 16th and 84th percentiles (marked with blue circles and numbered in blue); the 2nd and 98th percentiles (marked with red circles and numbered in red). The classical N-test based on a Poisson distribution with the mean value equal to the forecasted number of events ${N}_{\text{fore}}={\mathbb{E}}\left[N\left(M\ge {M}_{l}|{\varvec{\uptheta}},\mathbf{s}\mathbf{e}\mathbf{q}, {M}_{l}\right)\right]$(see “Methods” “N-test based on the simulation-based Bayesian framework” section and Eq. 12) is also shown on the same figure. It can be noticed that the distribution of $N\left(M\ge {M}_{l}|{\varvec{\uptheta}},\mathbf{s}\mathbf{e}\mathbf{q}, {M}_{l}\right)$ according to the histogram shows a larger spread with respect to the equivalent Poisson distribution. The terms ${\delta }_{1,\mathrm{ Poiss}}$ and ${\delta }_{2,\mathrm{ Poiss}}$ (based on the Poisson distribution), and ${\delta }_{1,\mathrm{ Bayes}}$ and ${\delta }_{2,\mathrm{ Bayes}}$ (based on the Bayesian simulation-based workflow) represent the probabilities that the number of events $n\le {N}_{\text{obs}}$ and $n\ge {N}_{\text{obs}}$ given ${N}_{\text{fore}}$ as described in “N-test based on the simulation-based Bayesian framework” section (“Methods”). They are used as an indicator for validating the forecasts within different time intervals.

The S-test (see “S-test based on the simulation-based Bayesian framework” section in “Methods”) is used to validate the spatial distribution of the forecasts provided by the proposed method. Figure 5c illustrates the S-test results regarding each forecasting interval based on the two methods described, namely, the standard and the Bayesian methods. It shows the distribution of the vector of the simulated log-likelihood values ${\mathbf{S}}_{\mathrm{sim}}$ (see Eq. 34) based on standard (in dark blue) and Bayesian (in light grey) histograms. The observed likelihood (*S*_obs_; see Eq. 33) is marked with red circle and numbered in red. The probability *P*(*S* ≤ *S*_obs_) based on both methods are reported on each figure. In the *Supplementary Information* (Section SI-6-Results), the forecasted seismicity maps, N-test and S-test results for *M* ≥ *M*_*l*_ are illustrated for the subsequent forecasting time windows (b-e) in Phase 1 (as shown in Table 1). Moreover, Fig. 9 (in “Daily forecasts of seismicity after the event Mw6.3 of 25/11/2018 (Phase 3)” section) shows the evolution of the N-test and S-test in the five forecasting intervals in Phase 1.

For this first and early forecasting interval (2 h and 42 min after the main event), **seq** includes 16 events with *M* ≥ 3.4. It is noted that the observed number of events (= 19) is equal to the ${N}_{\text{fore}}={\mathbb{E}}\left[N\left(M\ge {M}_{l}|{\varvec{\uptheta}},\mathbf{s}\mathbf{e}\mathbf{q}, {M}_{l}\right)\right]$ reported in Fig. 5b. The probabilities ${\delta }_{1}$ and ${\delta }_{2}$ (based on Bayesian method or Poisson distribution) are around 50% which indicate that the forecast matches observed number of events. Both methods for S-test indicate that the spatial distribution of forecasts reasonably matches the observed one.

### Daily forecasts of seismicity after the event Mw6.3 of 25/11/2018 (Phase 3)

**Figure 6:**
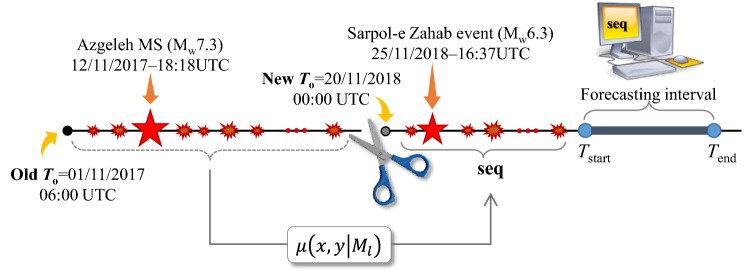
Sketch showing the shift in the time of origin *T*_o_.

Phase 3 of the complex Kermanshah seismic sequence 2017–2019 (described in Section “Introduction”) initiates by the Sarpol-e Zahab event of M_w_6.3, which took place in November 25, 2018, at 16:37 (UTC), around one year after the Azgeleh MS of M_w_7.3 in Phase 1. Given the time elapsed from the occurrence of the Azgeleh MS, it is computationally unjustifiable and tedious to consider entire **seq** consisted of the events of interest starting from the time origin *T*_o_ (i.e., 01/11/2017–6:00UTC used for Phase 1). To this end, we have shifted the time origin *T*_o_ from old *T*_o_ = 01/11/2017–06:00 UTC to new *T*_o_ = 20/11/2018–00:00 UTC (where new *T*_o_ is more than 5 days before the occurrence of M_w_6.3 earthquake), as shown in Fig. 6. With this shift in the time origin *T*_o_, the spatial rate of background seismicity, $\mu \left(x,y\left|{M}_{l}\right.\right)$ (see “Robust estimation for the number of aftershock events” section), is updated with respect to the long-term seismicity used for Phase 1 of the sequence. All the seismicity data from the old *T*_o_ up to the new *T*_o_, in addition to the long-term background seismicity is used to calculate the updated $\mu \left(x,y\left|{M}_{l}\right.\right)$ that is added as a constant term to the contribution of the triggering events (see “Robust estimation for the number of aftershock events” section). Herein, we use this background seismicity to conservatively approximate the triggering effect of the events that occurred in the first part of the sequence in the time interval [old *T*_o_ – new *T*_o_] (see the following “Estimating the updated background seismicity” section for the calculation of the updated $\mu \left(x,y\left|{M}_{l}\right.\right)$ in detail).

**Figure 7:**
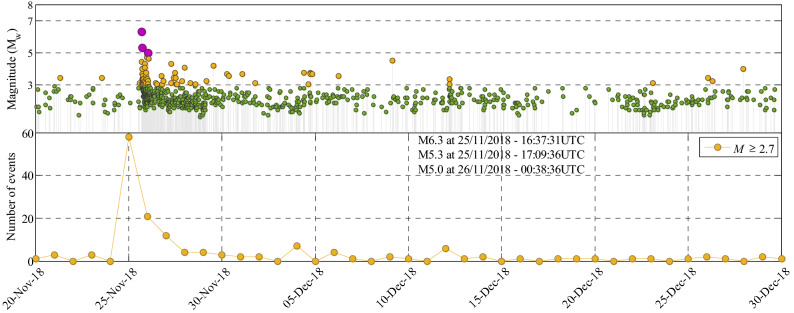
Magnitude vs. time (top) and the number of events with *M* ≥ 2.7 (bottom) occurred within a 24-h interval starting from 0:00 UTC of each day from 20/11/2018 (new *T*_o_) until 30/12/1017 including the Sarpol-e Zahab event of M_w_6.3 within phase 3 of the Kermanshah 2017–2019 seismic sequence.

The reference-time shift has been shown^28^ to work well in providing operational seismicity forecasts and avoids considering all the observed data within the **seq** (this claim will be supported by further studies in “6-h forecasting for the time interval [25/11/2018–18:00 UTC-26/11/2018–00:00 UTC]” section). We set ${{M}_{l}=M}_{c}=2.70$ (see *Supplementary Information* Section SI-7-Results, Figure SI-14a and its discussion). Figure 7 (top) shows the evolution of the events within Phase 3, and Fig. 7 (bottom) shows the evolution of the number of events in phase 3 starting from new *T*_o_ in the AS zone and with *M* ≥ 2.7 (orange circles) within a 24-h interval starting from 0:00 UTC each day (also reported at 0:00 UTC each day). The seismicity after the occurrence of M_w_6.3 increases considerably on 25 November and decreases very quickly the day after (around 20 events with *M* ≥ 2.70 occurred). Note also the two triggered events with very close epicenters of M_w_5.3 and M_w_5.0 occurred on 25/11/2018 and 26/11/2018, respectively. In the following, we describe the forecasting for the period from 25 to 26th of November 2018.

#### Estimating the updated background seismicity

The rate of seismicity is estimated based on **seq** consisting of 1337 events with $M\ge {M}_{l}=2.7$ (*Supplementary Information* Section SI-7-Results, Figure SI-14a) in the interval [old *T*_o_ – new *T*_o_] (Fig. 6). The ETAS model parameter **θ** is estimated with the same priors described in “Bayesian Inference for **θ**” section (the last row in Table 1). The background seismicity is drawn from the long-term seismicity of the site that is the same used in Phase 1. The histograms of marginal posterior PDFs for the model parameters **θ** together with their statistics are shown in first row of Table SI-2 (*Supplementary Information* Section SI-8-Results, labeled a). It is noted that parameter *p* is estimated almost close to 1.0 with a very small CI = [1.04, 1.12]. The median of these model parameters will be used as the new priors for the upcoming forecasting intervals. The correlations between ETAS parameters are also reported and discussed in Section SI-9-Results (first correlation matrix). The forecasted seismicity within the 24-h interval, [20/11/2018–00:00 UTC – 21/11/2018–00:00 UTC], is estimated in terms of the expected number of events in the spatial cell unit with $M\ge {M}_{l}=2.7$ denoted as ${\mathbb{E}}[N(x, y, {M\ge M}_{l}|\mathbf{s}\mathbf{e}\mathbf{q}, {M}_{l})]$ (see Eq. 9); thus, the updated background seismicity rate is $\mu \left(x,y\left|{M}_{l}\right.\right)$=${\mathbb{E}}[N(x, y, {M\ge M}_{l}|\mathbf{s}\mathbf{e}\mathbf{q}, {M}_{l})]$ (“Robust estimation for the number of aftershock events” section in “Methods”), which includes the rate of background events due to the long-term seismicity plus the triggering effect of the events in **seq**. The updated $\mu \left(x,y\left|{M}_{l}\right.\right)$ is used as a constant background rate in Eq. (7) to Eq. (9), and it conservatively does not represent the expected decay with time after the new *T*_o_. $\mu \left(x,y\left|{M}_{l}\right.\right)$ is plotted in Figure SI-18a (Section SI-10-Results). It is observed that the updated background seismicity has increased more than 300 times and that the probability of occurrence of $M\ge 6$ is more than 12 times higher the same probability estimated based on the long-term background seismicity, which reveals an alarming level (this was verified by Sarpol-e Zahab event of M_w_6.3).

#### 6-h forecasting for the time interval [25/11/2018–18:00 UTC-26/11/2018–00:00 UTC]

After the occurrence of Sarpol-e Sahab event with M_w_6.3 at 16:37 UTC of 25/11/2018, we provide a 6-h prediction of seismicity for the forecasting interval starting from *T*_*start*_ set to 18:00 UTC (i.e., 1 h and 23 min after the occurrence of M_w_6.3 event). Considering new *T*_o_ (Fig. 6), the data within the time interval [new *T*_o_ = 20/11/2018–00:00 UTC, *T*_*start*_ = 25/11/2018–18:00 UTC] forms the **seq**, which includes 27 events with $M\ge {M}_{l}=2.7$ (${M}_{l}={M}_{c}$; see Section SI-7-Results, Figure SI-14b and its discussion). The histograms of marginal posterior PDFs for the model parameters **θ** together with their statistics are shown in second row of Table SI-2 (Section SI-8-Results, labeled b). The spatial distribution of the forecasted seismicity with $M\ge 2.7$ is shown in Figure SI-19a (Section SI-10-Results). Observed events with $M\ge 2.7$ are also shown on the map. The Sarpol-e Zahab M_w_6.3 is shown with red-colored hexagram to show the epicenter of the main event –although this event is not within the observed events in the forecasting time interval. According to the error-bar plot in the same figure, the total number of registered events within the 6-h forecasting interval (green star; = 38) is higher than the forecasted values (median; = 18). This can be attributed (1) to the small number of observed input data in **seq**; (2) the presence of the triggered event with M_w_5.3 at 17:09. The N-test result in Figure SI-19b shows the histogram for the distribution of the number of events based on the Bayesian workflow with ${N}_{\text{fore}}={\mathbb{E}}\left[N\left(M\ge 2.70|{\varvec{\uptheta}},\mathbf{s}\mathbf{e}\mathbf{q}, {M}_{l}=2.70\right)\right]=18$ and other related statistics (similar to the error-bar chart in Figure SI-19a). The probabilities ${\delta }_{1}$ and ${\delta }_{2}$ (based on Bayesian method or Poisson distribution) show that the real number of events falls in the upper tail of the distributions (i.e., underestimation in the forecasted seismicity). However, the distribution of the forecasted seismicity is validated through the standard/Bayesian S-test method (Figure SI-19c) that shows a 30% value for the probability *P*(*S* ≤ *S*_obs_). Although less successful in predicting the number of events, Figure SI-19a reports exceedance probabilities *P*(*M* ≥ 5) more than 30 times and *P*(*M* ≥ 6) around 90 times compared to their corresponding initial estimates based on the updated background seismicity in Figure SI-18a. In order to validate the reference-time shift consideration, we perform the same forecast done here considering the whole events with $M\ge {M}_{l}=2.7$ in the sequence (= 1364) starting from [old *T*_o_ = 01/11/2017–06:00 UTC, *T*_*start*_ = 25/11/2018–18:00 UTC], which form the **seq**. The marginal distributions of the ETAS model parameters have smaller confidence interval compared to those reported in Table SI-2 (row b) as the Bayesian updating of model parameters is done based on higher data points in the observation history. Figure SI-19d shows the spatial distribution of the forecasted seismicity, where the Azgeleh M_w_7.3 mainshock is indicated by a red-colored hexagram (while the Sarpol-e Zahab M_w_6.3 is illustrated with magenta-colored triangle). The error-bar plot in the same figure shows the distribution of the forecasted number of events with median = 15 (event) and a smaller confidence interval (as noted previously) with respect to the similar error-bar in Figure SI-19a. The N-test result in Figure SI-19e shows the histogram for the distribution of the number of events with smaller dispersion (compared to Figure SI-19b) with ${N}_{\text{fore}}=15$ being close to the previous estimate in Figure SI-19b (= 18) that is obtained based on the shift in the time origin. With reference to the S-test (Figure SI-19f.), the distribution of the forecasted seismicity is even better predicted with *P*(*S* ≤ *S*_obs_) more than 70%. As a result, the shift in the time of origin (herein, more than one year) of the sequence, by introducing a non-uniformly distributed background seismicity, does not affect the results of our forecast overall while it relieves the computational cost of summing up the triggering properties of all the events that took place in an ongoing sequence in the time interval of more than one year. Neglecting the time-decay in the triggering of the events before “new *T*_o_” can even furnish more conservative estimates.

#### 5-h forecasting for the time interval [25/11/2018–19:00 UTC-26/11/2018–00:00 UTC] and two subsequent forecasts

To get more reliable early forecasts, we set *T*_*start*_ = 25/11/2018–19:00 UTC (i.e., 2 h and 23 min after the occurrence of M_w_6.3 event) and keep the same *T*_*end*_. For this forecasting interval, ${M}_{c}=2.70$ (Section SI-7-Results). Thus, **seq** includes 43 events occurred from new *T*_o_ up to *T*_*start*_ with $M\ge {M}_{l}=2.7$ (where ${M}_{l}={M}_{c}$). The marginal distribution of the updated ETAS model parameters **θ** is shown in Table SI-2, (Section SI-8-Results, labeled c). The spatial distribution of the forecasted seismicity (the expected value for the number of events with $M\ge 2.7$), the N-test, and S-test results are shown in Figure SI-20 (Section SI-10-Results) for this 5-h forecasting interval. The total number of registered events (green star; = 22) is within + 1 standard deviation confidence interval (also verified by the probabilities ${\delta }_{1}$ and ${\delta }_{2}$). The S-test also shows that the forecasted spatial distribution of seismicity matches quite well the observed spatial pattern.

**Figure 8:**
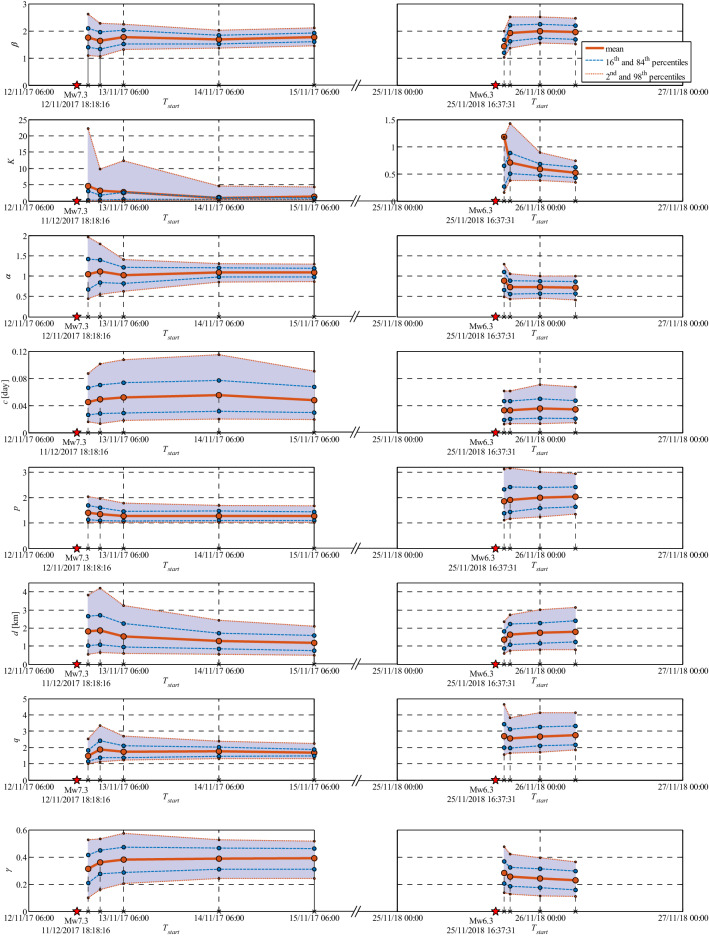
Summary and timeline of the evolution of ETAS model parameters for Phase 1 (left column) and Phase 3 (right column).

Seismicity forecasts are also provided for two subsequent back-to-back intervals; namely, a 6-h time interval with *T*_*start*_ = 26/11/2018–00:00UTC and a 18-h forecast with *T*_*start*_ = 6:00UTC of the same day with ${M}_{c}=2.70$ (Section SI-7-Results). The last two rows of Table Table SI-2 (d, e) illustrate the statistics of updated model parameters **θ**. The evolution of ETAS model parameters for Phase 3 of the seismic sequence is shown in Fig. 8. It can be seen that all parameters start to stabilize after the early forecasts. As expected, parameter *β* stabilizes to a slightly larger value in Phase 3 (1.95); in both phases it is estimated to be larger than background *β* (1.67). In both phases, *α* is smaller than β (sub-critical process). As expected, *α* for Phase 3 is smaller than that of Phase 1, which is also expected given the lower productivity of this phase. Note that the value of *K* shows discontinuity between the two phases, this is due to the shift in the time of origin. The larger *p* value in Phase 3 indicates a steeper time decay. The same is through for parameter *q* which indicates a stronger spatial attenuation in Phase 3. Parameter *γ* indicates a stronger magnitude dependence of the spatial kernel in Phase 1 compared with Phase 3.

**Figure 9:**
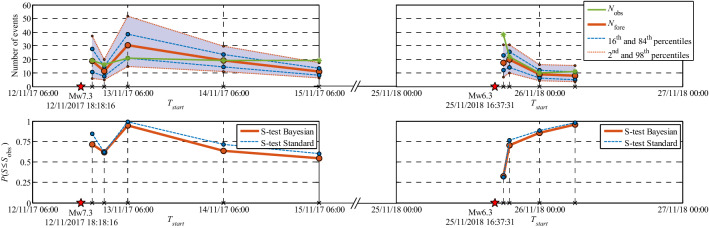
Summary and timeline of the evolution of N-Test and S-test results for Phase 1 (left column) and Phase 3 (right column).

The predicted seismicity distribution maps and the corresponding error-bar plots, together with the N-test and S-test results are shown in Figures SI-21 and SI-22 (Section SI-10-Results). For the 6-h forecasting interval (Figure SI-21a), the M_w_5.0 event occurred 38 min after *T*_*start*_ (see also Fig. 7). The forecasts for these two latter time intervals manage to properly predict the observed seismicity in terms of the number of events with $M\ge 2.7$, as well as its spatial content through the seismicity tests. The correlations between ETAS parameters for the four forecasting intervals in Phase 3 are reported and discussed in detail in Section SI-9-Results. Figure 9 summarizes the evolution of the N-test and S-test in the four forecasting intervals in Phase 3.

### Sensitivity analyses

In this section, the sensitivity of the resulting robust forecasts to some possible variations to the proposed method are investigated.

#### Using the simple spatial kernel density compared to the magnitude-dependent kernel density

An alternative kernel density, denoted herein as the simple spatial kernel density with two spatial term parameters (*d*, *q*), can be used instead of the magnitude-dependent kernel with the three related parameters (*d*, *q*, *γ*). Table SI-3 (Section SI-11-Results) shows the histograms of marginal posterior PDFs for the six ETAS model parameters **θ** = [*β*, *α*, *c*, *p*, *d*, *q*] where the simple spatial kernel density term is used. The distribution of parameter $\beta$ and its statistics matches very well those reported in Table 1. The mean values of Parameter $\alpha \cong 0.70-0.80$ are around 2/3 of those reported in Table 1. The distribution of temporal parameters (*c*, *p*) are more-or-less invariant. The posterior distributions of spatial parameters (*d*, *q*) are different (see Table 1) in the sense that parameter *d* has a higher mean and dispersion, while parameter *q* behaves in an opposite manner. Parameter *K* has a higher estimate in case of simple spatial kernel density (to be expected since it has to fulfill Eq. (13) for each realization of **θ**). The correlation matrices of 7 model parameters are illustrated in Section SI-11-Results for different forecasting intervals. Comparing the correlation structure with those obtained based on the magnitude-dependent spatial kernel density (Fig. 4 and Section SI-5), it is revealed that *β* has no correlation and *K* reveals a sort of small correlations with other ETAS parameters (similar behavior for both kernels). Parameter *α* has smaller correlation with temporal parameters (*c* and *p*), and spatial parameters (*d*, *q*) in case of simple kernel density. This is the reason for having different estimates for this parameter between the two spatial models. In the magnitude-dependent kernel density model, *α* reveals more correlation with the magnitude-related parameter *γ* compared to the other parameters (see Fig. 4 and Section SI-5). The correlations between temporal parameters (*c*, *p*) and spatial parameters (*d*, *q*) increases as the sequence evolves. It is to note that in the magnitude-dependent spatial kernel density model, there is no dependence between the spatial parameters (*d*, *q*).

Table 2 compares the results of seismicity forecasting test, N-test and S-test, based on alternative spatial kernel density terms, for the five forecasting intervals indicated in Table 1. Both models for spatial kernel density are similarly evaluated through the N-test and S-test. It is interesting to highlight that S-test results in terms of $P\left(S\le {S}_{\mathrm{obs}}\right)$ becomes higher as the forecast evolves showing that the simple kernel density provides a better spatial forecast of seismicity compared to the more complex magnitude-dependent model (although this might not be a general conclusion).

**Table 2:**
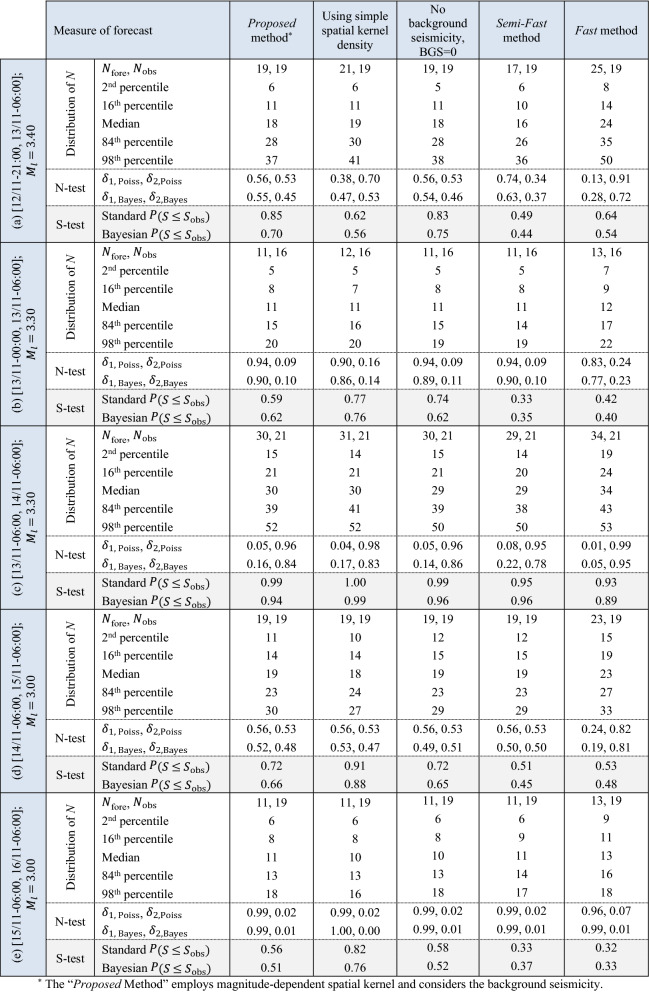
N-test and S-test results in different forecasting intervals considering different hypotheses.

#### Considering background seismicity

Since the background seismicity is low compared to the big jump in seismicity due to the M_w_7.3 mainshock for Phase 1 (i.e., the first term *N*_*b*_ compared to the second term in Eq. (7), “Methods”), it is expected that the effect of not considering the background seismicity (BGS) spatial data $\mu \left(x,y\left|{M}_{l}\right.\right)$ within the short term operational forecasting may not be significant. Table 2 compares the results of observations vs. forecasts in terms of N-test and S-test for the cases with BGS (*Proposed* method) with the case without BGS (BGS = 0) for all forecasting intervals indicated in Table 1. It can be observed from Table 2 that assuming BGS = 0 leads to no apparent difference with respect to considering BGS based on both N-test and S-test results. That is, neglecting the effect of long-term BGS within the proposed robust seismicity forecasting framework will not significantly affect the forecasted number of events. This appears to be useful while operational forecasting is undertaken within an ongoing seismic sequence with limited time and input data.

#### Effect of some simplifications within the robust seismicity forecasting framework

The proposed framework can be simplified/approximated by considering the following assumptions (see “Robust estimation for the number of aftershock events” section in “Methods”):

1. Characterizing parameter *K*: this parameter can be calculated through the closed-form expression in Eq. (17) (“Calculating *K*” section, *Calculate K*), or can be learnt through the Bayesian updating framework (“Calculating *K*” section, *Learn K*). It is to note that the parameter *K* learned through the Bayesian inference does not necessarily satisfy Eq. (13) for each sample **θ**.
2. The integral over the whole aftershock zone **A**: the term ${\mathrm{I}}_{r}$ (see Section “Methods”, Eq. (16)), can be solved analytically. However, by assuming an infinite spatial domain, ${\mathrm{I}}_{r}$ can be *approximated* with ${\widetilde{\mathrm{I}}}_{r}$, over the infinite space.

**Figure 10:**
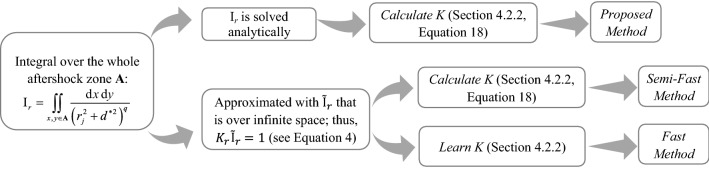
A schematic view of different approximations in the procedure to provide seismicity forecasts.

Figure 10 shows a general overview of schemes to provide seismicity forecasts based on different levels of approximation that encompass: (a) *Proposed method* is the suggested method; (b) *Semi-Fast method* approximates the integral over the aftershock zone with ${\widetilde{\mathrm{I}}}_{r}$; (c) *Fast method* learns *K* though Bayesian inference (i.e., **θ** = [*β*, *K*, *α*, *c*, *p*, *d*, *q*, *γ*]) and approximates the integral over the aftershock zone with ${\widetilde{\mathrm{I}}}_{r}$.

##### Discussion on the effect of considering the integration over the whole aftershock zone (using *Semi-Fast method*)

Table SI-4 (Section SI-12-Results) shows the histograms of marginal posterior PDFs for the model parameters **θ** together with their prior PDFs using *Semi-Fast method* as opposed to results shown in Table 1 for the *Proposed method*. Comparing these two Tables, apart from the spatial kernel density parameters of distance *d* and *q*, the statistics (mean and CI) of the other parameters are very close (as the parameter estimation evolves, they become even closer). Parameters *d* and *q* have higher estimates (consequently higher mean, 2%, and 98% statistics) in case of *Semi-Fast* method compared to the *Proposed method*. In terms of the correlation structure among the parameters, we have reported the correlation matrix between the ETAS model parameters **θ** = [*β*, *K*, *α*, *c*, *p*, *d*, *q*, *γ*] in Section SI-12-Results for *Semi-Fast method* which shows the same trends as those observed for the *Proposed method*.

Table 2 summarizes the N-test and S-test results of the *Semi-Fast method* to be contrasted with the first column of Table 2 associated with the *Proposed method*. There are some differences between the two: (1) The first forecasting interval for *Semi-Fast* has lower expected value (*N*_fore_) with respect to *Proposed method;* (2) the *Proposed method* outperforms the *Semi-Fast* based on S-test results. In terms of N-test, as the sequence evolves, the differences between the test results becomes negligible. This observation has been also made in^71^, where the assumption of an infinite spatial domain was shown to have negligible effect on likelihood. (3) Overall, the computational time for the *Semi-Fast method* is observed to be around 75% of that of the *Proposed method*.

##### Discussion on the effect of calculating *K*

Table SI-5 in Section SI-13-Results illustrates the histograms of marginal posterior PDFs for the model parameters **θ** using *Fast method*. It can be compared with Table SI-4 based on *Semi-Fast method*. The parameter *K* is learnt through the MCMC procedure assuming that the model parameter **θ** has a multivariate lognormal prior (see “Bayesian Inference for **θ**” section), for which we set a high COV = 1.0 for parameter *K* compared to other model parameters with COV = 0.5 (“Bayesian Inference for **θ**” section). In the ballpark, the mean estimates for parameter *K* are quite the same between *Fast* and *Semi-Fast* methods with the CI for *Semi-Fast method* being wider. Although low number of observed data is available for learning *K* through the Bayesian framework at the very beginning of Phase 1, *K* does not show sensitivity to the choice of the prior in the *Fast method* (see Table SI-5). Parameter $\alpha$ has lower estimates based on the *Fast method*. However, as the sequence evolves, it gets closer to *Semi-Fast* estimates. The most critical parameter in the *Fast method* seems to be the temporal decay random variable *p*, which has a mean estimate very close to its lower threshold (= 1.0) and a very low variability. The temporal parameter *c* as well as the spatial parameters (*d*, *q*, *γ*) are quite close to those from the *Semi-Fast method*.

We have reported the correlation matrix between the ETAS model parameters in *Supplementary Information* (Section SI-13-Results) for *Fast method*. Compared to previous discussions on the correlation of the ETAS parameters by employing the *Proposed* and *Semi-Fast* methods, parameter *β* shows higher correlations with other model parameters while using *Fast* *method* (although the absolute value of the correlation is small). Parameter *K* shows a very high correlation with the temporal parameter *p*, and as the sequence evolves, this negative correlation increases (changing from around -55% to -80%). The negative correlations also appear in the *Proposed*/*Semi-Fast* methods but with lower intensities. This high correlation between (*K*, *p)* affects the correlation between (*c*, *p*) so that the amount of correlation between the latter two parameters are smaller than those appear in the *Proposed*/*Semi-Fast*. Parameter *α* shows the same order of correlation with temporal/spatial parameters in the *Fast* method compared to the other two methods. Higher correlations appear between *p* and the parameters of the spatial kernel density (compared to the *Semi-Fast* *method*). Like the previous two methods, there is a high negative correlation between (*d*, *γ*), and as the sequence evolves, the positive correlation between (*q*, *γ*) decreases.

Table 2 summarizes the N-test and S-test results of the *Fast method*. Comparing *Fast*/*Semi-Fast* (see the last two columns of Table 2): (1) For the first forecasting interval, *Fast method* slightly overestimated the mean number of forecasted events (*N*_fore_ = 25 vs 19 registered events with *M* ≥ 3.4 took place) and the distribution of the forecasted number of events shows higher dispersion with respect to *Semi-Fast* *method*; (2) As the sequence evolves, the forecasts issued by the *Fast method* gets closer to those of *Semi-Fast method* (and consequently the *Proposed method*). This shows that *Fast method* is more reliable as the sequence evolves. However, like the *Semi-Fast method*, S-test shows that this method cannot predict the spatial pattern of seismicity as perfect as the Proposed method (it stills passes the test). (3) Overall, the time of conducting the *Fast method* is less than 50% of that for *Semi-Fast method* and 37% of that for *Proposed method*, making the procedure appealing for operational earthquake forecasting.

Based on the above discussions, it is recommended to do the *Proposed* *method* at least for very early forecasts. As the sequence evolves, the computationally less time-consuming *Fast* (or even the *Semi-Fast*) methods can be used.

## Discussion and conclusions

We have improved and tested a Bayesian and fully simulation-based workflow for spatio-temporal early seismicity forecasting based on ETAS model. This workflow encompasses new/modified features of the Bayesian and fully probabilistic seismicity forecasting framework proposed previously by the first two authors^28^. Regarding the Bayesian updating of the ETAS model parameters, the improvements concern (a) modification of the likelihood function and its calculation; (b) adoption of an adaptive MCMC simulation technique by using multi-dimensional kernel sampling density functions; (c) derivation of a close-form expression for productivity parameter *K* as a function of other ETAS parameters; (d) incorporation of seismicity tests such as N-test and S-test in the proposed workflow to see how well the forecasted number of events and the forecasted spatial distribution of seismicity match the observed ones. Additionally, the improved workflow is capable of (a) analytically solving the integral of the spatial kernel density over the whole aftershock zone instead of assuming it to be equal to unity; (b) incorporate the background seismicity within the robust seismicity forecasting framework; (c) consider magnitude-dependent spatial kernel density in the ETAS model.

As a demonstration of this procedure, we have applied the fully simulation-based workflow for providing retrospective early forecasts of seismicity in two distinct phases of the recent Kermanshah 2017–2019 seismic sequence in western Iran starting with the events of M_w_7.3 and M_w_6.3, respectively. Here are some observations:

- After an initial transition time in the order of few hours (2 h and 42 min after the main event of M_w_7.3 in Phase 1, and 2 h and 23 min after the occurrence of M_w_6.3 in Phase 3), the model tunes into the sequence and provides very reliable forecasts.
- The workflow traces the time evolution of the ETAS model parameters as the sequence goes on. Overall, the parameters tend to stabilize after the first early forecast intervals. Evolution of seismicity parameter *β* matches the expectations (a slight increase with respect to background seismicity). Parameter *α* is always smaller than *β* ensuring sub-critical behavior in the point process. Comparing Phase 1 and Phase 3, the ETAS parameters in Phase 3 reveal a seismic sequence with weaker productivity and stronger spatio-temporal decay, as matched by the observations.
- The productivity coefficient *K* is derived based on the constraint that the observed number of events previous to the time of starting the forecast is equal to the forecast provided based on each realization of the vector of model parameters (*Proposed method*). Alternatively, one can choose to learn *K* together with other ETAS model parameters (called herein *Fast method*) through the Bayesian inference. This is going to reduce the computational time by around 37% at the cost of losing accuracy in the early forecasting intervals.
- Neglecting the long-term background seismicity does not significantly affect the forecasted number of events in this study (Phase 1 forecasts).
- For providing early forecast of seismicity for M_w_6.3 event in Phase 3, we performed a shift in the time of origin (more than one year) of the sequence by introducing a non-uniformly distributed background seismicity (which is calculated by exploiting the proposed method). This shift relieves the computational burden of summing up the triggering properties of all the events that took place in an ongoing sequence at the cost of neglecting the (negligible) time-decay (beyond the first day of Phase 3) in their triggering contribution.
- Adoption of a magnitude-dependent spatial kernel (compared to a simpler functional form) shows no significant differences in the seismicity forecasting estimates. However, S-test results reveal that the simple kernel performs better in forecasting the spatial distribution of seismicity.
- The integral of the spatial kernel density over the whole aftershock zone can be solved analytically (*Proposed method*) or can be approximated by assuming that the integral is over an infinite spatial domain (*Semi-Fast method*). In the early stage of forecasting, the *Proposed method* outperforms *the Semi-Fast method* based on both N-test and S-test results. As the sequence evolves, the differences between the test results becomes negligible. However, the computational time of conducting the *Semi-Fast method* is around 75% of the required time for performing *Proposed method*. Therefore, the use of *Semi-Fast method* is only recommended for providing forecasts as the sequence evolves.

## Methods

### The epidemic-type aftershock sequence (ETAS) model for space–time clustering of aftershocks

The ETAS model^11,12^ is a marked stochastic point process model widely used to describe the temporal and spatial clustering of seismicity (see also^13–16,57^). In this model, every earthquake (being spontaneous or triggered) is a potential triggering event for its own subsequent earthquakes (aftershocks). Let *λ*_ETAS_(*t*, *x*, *y*, *m*|**θ**, **seq**_*t*_, *M*_*l*_) denote the conditional rate of occurrence of events with magnitude *M* greater than or equal to *m* (the seismicity rate) based on the ETAS model at time *t* in the cell unit centered at the Cartesian coordinate (*x*, *y*)$\in$ **A** where **A** is the aftershock zone. The Cartesian area can be discretized into mutually exclusive and collectively exhaustive, MECE, subsets or spatial cell units centered at *x* and *y*. The rate *λ*_ETAS_ is conditioned on: the vector of ETAS model parameters **θ** (defined subsequently); observation history up to the time *t* denoted as **seq**_*t*_ = {(*t*_*j*_, *x*_*j*_ , *y*_*j*_, *m*_*j*_), *t*_*j*_ < *t*, *m*_*j*_ ≥ *M*_*l*_} where *t*_*j*_ is the occurrence time of the *j*th event (with *t*_*j*_ < *t*) with magnitude *m*_*j*_ and location (*x*_*j*_, *y*_*j*_)$\in$ **A**; the *lower magnitude M*_*l*_ (should be greater than or equal to the *completeness magnitude M*_*c*_ of the catalog of events up to the time *t*; i.e. *M*_*l*_ ≥ *M*_*c*_; see the note on the calculation of *M*_*c*_ at the end of this section). *λ*_ETAS_(.) can be shown as follows:

1 $$\lambda_{{\,{\text{ETAS}}}} \left( {\left. {t,x,y,m} \right|{{\varvec{\uptheta}}},{\mathbf{seq}}_{t} ,M_{l} } \right) = P\left( {M \ge m|{{\varvec{\uptheta}}},M_{l} } \right) \cdot \lambda_{{\,{\text{ETAS}}}} \left( {\left. {t,x,y} \right|{{\varvec{\uptheta}}},{\mathbf{seq}}_{t} ,M_{l} } \right)$$

where *P*(*M* ≥ *m*|**θ**, *M*_*l*_) or the “marker” is the conditional probability that *M* ≥ *m* given **θ** and *M*_*l*_ and λ_ETAS_(*t*, *x*, *y*|**θ**, **seq**_*t*_, *M*_*l*_) is the conditional seismicity rate based on ETAS model given **θ seq**_*t*_ and *M*_*l*_. The spatio-temporal triggering effect of a given sequence on the seismicity rate can be seen through *λ*_ETAS_(*t*, *x*, *y*|**θ**, **seq**_*t*_, *M*_*l*_). Using a truncated exponential (Gutenberg-Richter) model, it is easy to show that $P\left(M\ge m\left|{\varvec{\uptheta}},\right.{M}_{l}\right)=\mathrm{exp}\left(-\beta \left(m-{M}_{l}\right)\right)$, which is the CCDF (complementary cumulative density function) of an exponential distribution, and *β* is related to Gutenberg-Richter relation. Equation (1) can be written according to the general ETAS model as follows:

2 $$\lambda_{{\,{\text{ETAS}}}} \left( {\left. {t,x,y,m} \right|{{\varvec{\uptheta}}},{\mathbf{seq}}_{t} ,M_{l} } \right) = \underbrace {{e^{{ - \beta \,\left( {m - M_{l} } \right)}} }}_{{P\left( {M \ge m|{{\varvec{\uptheta}}},M_{l} } \right)}} \cdot \underbrace {{\sum\limits_{{t_{j} < t}} {Ke^{{\alpha \left( {m_{j} - M_{l} } \right)}} \cdot \frac{{K_{t} }}{{\left( {t - t_{j} + c} \right)^{p} }} \cdot \frac{{K_{r} }}{{\left( {r_{j}^{2} + d^{ * 2} } \right)^{q} }}} }}_{{\lambda_{{\,{\text{ETAS}}}} \left( {\left. {t,x,y} \right|{{\varvec{\uptheta}}},{\mathbf{seq}}_{t} ,M_{l} } \right)}}$$

The conditional (triggering) rate *λ*_ETAS_(*t*, *x*, *y*|**θ**, **seq**_*t*_, *M*_*l*_) in Eq. (2) is a summation taken over every *j*th event occurred before time *t*. It is comprised of three terms as follows:

- (a) The productivity function $K{e}^{\alpha \left({m}_{j}-{M}_{l}\right)}$ represents the number of events with magnitudes equal to or greater than *M*_*l*_ (productivity of event ${m}_{j}$; see^11^), where *α* (magnitude^−1^) determines the exponential increase due to events with magnitudes larger than *M*_*l*_ and *K* (shocks or events) is the productivity coefficient and measures the intensity of the exponential function in generating triggered aftershocks.
- (b) The typical normalized aftershock time decay law with the functional from ${K}_{t}/{\left(t-{t}_{j}+c\right)}^{p}$ is a temporal distribution based on modified Omori (MO) model^17,72^. Utsu^17^ showed that the occurrence rate of aftershocks has a functional form being inversely proportional to the time to the power of *p*, for which the *p* value affects the decay rate of aftershocks in time. The parameter *c* (time, e.g. day) eliminates the singularity issue at *t* = *t*_*j*_, and partially reflects the effect of incomplete detection of small aftershocks taken place shortly after the mainshock^72^. The parameter *K*_*t*_ is a normalizing coefficient that satisfies the achievement of asymptotic compatibility between ETAS prediction and the long-term seismicity, i.e., integrating the time-dependent term over infinite time will in limit be equal to unity (see also^11,72,73^):
  3 $$K_{t} \int\limits_{{t_{j} }}^{ + \infty } {\frac{{{\text{d}}t}}{{\left( {t - t_{j} + c} \right)^{p} }}} = 1 \Rightarrow K_{t} \left( {\frac{{c^{1 - p} }}{p - 1} - \left. {\frac{{\left( {t - t_{j} + c} \right)^{1 - p} }}{p - 1}} \right|_{\,t \to + \infty } } \right) = 1\,\,\,\mathop{\longrightarrow}\limits^{p\,\, > \,\,1}\,\,K_{t} = \left( {p - 1} \right)\,c^{p - 1}$$
  It is to note that when *p* > 1, the integral converges at *t* →  + $\infty$, while for *p* < 1, it goes to infinity as *t* →  + $\infty$. Thus, the condition *p* > 1 is an important constraint in the ETAS parameter estimation. Detailed discussions on other decay models have been furnished in^74^.
- (c) The term ${K}_{r}/{\left({{r}_{j}}^{2}+{{d}^{\divideontimes }}^{2}\right)}^{q}$ is the joint (two-dimensional) probability density function of coordinates (*x*,*y*)$\in$ **A** around the epicenter of the *j*th event (*x*_*j*_, *y*_*j*_), with *r*_*j*_ the distance between the location (*x*, *y*) and the epicenter (*x*_*j*_, *y*_*j*_); i.e., ${{r}_{j}}^{2}={\left(x-{x}_{j}\right)}^{2}+{\left(y-{y}_{j}\right)}^{2}$. Herein, the following two functions are used as ${d}^{\divideontimes }$:
  - ${d} ^{\divideontimes }=d$: the so-called *simple spatial kernel density* herein is characterized by two parameters *d* (distance, e.g. km) and *q* defining the spatial distribution (decay) of the direct offspring triggered by the *j*th event with epicenter at (*x*_*j*_, *y*_*j*_). This spatial decay footprint model is a function of only (*x*-*x*_*j*_, *y*-*y*_*j*_) with a circular symmetry around (*x*_*j*_, *y*_*j*_). It has been used by many researchers before (see e.g.,^12,21,75^). The normalizing coefficient *K*_*r*_ is defined such that integrating the spatial term over infinite space will in limit be equal to unity (see also^75^):
    4 $$\begin{gathered} \int\limits_{ - \infty }^{ + \infty } {\int\limits_{ - \infty }^{ + \infty } {\frac{{K_{r} {\text{d}}x{\text{d}}y}}{{\left( {(x - x_{j} )^{2} + (y - y_{j} )^{2} + d^{2} } \right)^{q} }}} } = K_{r} \int\limits_{0}^{ + \infty } {\frac{{2\pi r{\text{d}}r}}{{\left[ {r^{2} + d^{2} } \right]^{q} }}} = 1\quad \mathop{\longrightarrow}\limits^{q\,\, > \,\,1}\,\,K_{r} = \frac{(q - 1)}{\pi }d^{{\,2\,\left( {q - 1} \right)}} \hfill \\ \Rightarrow \frac{{K_{r} }}{{\left( {r_{j}^{2} + d^{ * 2} } \right)^{q} }} = \frac{(q - 1)}{\pi }d^{{\,2\,\left( {q - 1} \right)}} \cdot \frac{1}{{\left( {r_{j}^{2} + d^{2} } \right)^{q} }} \hfill \\ \end{gathered}$$
    when *q* > 1, the integral converges at *r* →  + $\infty$. Thus, the condition *q* > 1 is another key constraint the ETAS model parameter estimation. It is shown in different studies (see e.g.,^12,75^) that this spatial model fits the data better than the normal distribution model (being also proposed as a spatial model) expressed by $\frac{1}{2\pi {d}^{2}}{e}^{-{{r}_{j}}^{2}/2{d}^{2}}$. The latter is characterized by one parameter while the model used herein has two parameters.
  - ${d}^{\divideontimes }=d{e}^{\gamma {m}_{j}}$: the so-called *magnitude-dependent spatial kernel density* herein is a widely used model^15,76^, has three parameters (*d*, *q*, and *γ*), and is characterized by introducing a decay model that takes into account also the magnitude of the triggered events *m*_*j*_. The kernel density, that accounts for the observed exponential increase of the rupture dimensions with earthquake magnitude^77^, is expressed as follows:
    5 $$\frac{{K_{r} }}{{\left( {r_{j}^{2} + d^{ * 2} } \right)^{q} }} = \underbrace {{\frac{(q - 1)}{\pi }\left( {de^{{\gamma m_{j} }} } \right)^{{\,2\,\left( {q - 1} \right)}} }}_{{ = K_{r} }} \cdot \frac{1}{{\left( {r_{j}^{2} + \left( {de^{{\gamma m_{j} }} } \right)^{2} } \right)^{q} }} = \frac{(q - 1)}{{\pi d^{2} e^{{2\gamma m_{j} }} }} \cdot \left( {1 + \frac{{r_{j}^{2} }}{{d^{2} e^{{2\gamma m_{j} }} }}} \right)^{ - q}$$

These two spatial kernels are both isotropic. Recent study^78^ shows the superiority of anisotropic spatial kernel especially in the presence of earthquake doublets. With reference to Eq. (2), the ETAS model parameters **θ** can have seven parameters **θ** = [*β*, *K*, *α*, *c*, *p*, *d*, *q*] using the simple spatial kernel density with ${d}^{\divideontimes }=d$, or eight parameters **θ** = [*β*, *K*, *α*, *c*, *p*, *d*, *q*, *γ*] employing the magnitude-dependent spatial kernel density with ${d}^{\divideontimes }=d{e}^{\gamma {m}_{j}}$. Strictly speaking, the derived parameters *K*_*t*_ (see Eq. (3), function of *c* and *p*), and ${K}_{r}=\frac{q-1}{\pi }{{d}^{\divideontimes }}^{2\left(q-1\right)}$ (see Eq. (4) as function of *d* and *q*, or Eq. (5) as a function of *d*, *q* and *γ*) satisfy the achievement of asymptotic compatibility between ETAS predictions and the long-term seismicity. Parameter *K*, which has a direct relation with the number of events taken place before time *t*, will be discussed later (see “Calculating *K*” section). The rate of events with magnitude (exactly) equal to *m*, denoted as *λ*_ETAS_(*t*, *x*, *y*, *M* = *m*|**θ**, **seq**_*t*_, *M*_*l*_) herein, is calculated by taking the derivative of Eq. (1) with respect to magnitude *m*:

6 $$\lambda_{{\,{\text{ETAS}}}} \left( {t,x,y,M = m|{{\varvec{\uptheta}}},{\mathbf{seq}}_{t} ,M_{l} } \right) = \left| {\frac{\partial }{\partial m}\lambda_{{\,{\text{ETAS}}}} \left( {t,x,y,m|{{\varvec{\uptheta}}},{\mathbf{seq}}_{t} ,M_{l} } \right)} \right| = \,\underbrace {{\beta e^{{ - \beta \,\left( {m - M_{l} } \right)}} }}_{{p\left( {M = m|{{\varvec{\uptheta}}},M_{l} } \right)}} \cdot \lambda_{{\,{\text{ETAS}}}} \left( {t,x,y|{{\varvec{\uptheta}}},{\mathbf{seq}}_{t} ,M_{l} } \right)$$

where *p*(*M* = *m*|**θ**, *M*_*l*_) is the probability density function, PDF, of magnitude at *m*.

*Note on the estimation of the completeness magnitude* *M*_***c***_**:** It is known that aftershocks catalogs compiled immediately following a strong, shallow mainshock are not completely registered^72,79–82^. Therefore, for providing early forecasts, proper estimation of ${M}_{c}$ is a significant component for defining the observed aftershock sequence (observation history) to estimate the rate *λ*_ETAS_ (see Eq. (2)). It is noted that having a high estimate for ${M}_{c}$ would deprive the data of potentially valuable information that are crucial for Bayesian updating of ETAS model parameters (see “Sampling **θ** from the distribution *p*(**θ**|**seq**, *M*_*l*_)” section). Herein, we have employed three different strategies for estimation of ${M}_{c}$ (see *Supplementary Information* Section SI-3-Results for a detailed discussion): (1) Plotting the frequency-magnitude distribution of events in the observation history (available data at the time of forecasting) in a semi-logarithmic scale and visually detecting ${M}_{c}$ as the point where the curve becomes approximately linear after the flat lower magnitude ranges. (2) Employing Bayesian inference for plotting the maximum likelihood of the posterior probability distribution of *β* versus various magnitude thresholds; ${M}_{c}$ can be detected as the magnitude threshold, where the plot of maximum likelihood estimates of *β* becomes quasi-invariant or reaches its peak value. (3) The semi-logarithmic plot, showing the observed earthquake magnitudes as a function of the time elapsed after the mainshock, is a visual check for the value of ${M}_{c}$ estimated by the previous two approaches. This is to ensure that the observed catalog of data at the time of issuing a forecast or the time of interest is complete for magnitudes $\ge {M}_{c}$ and does not include small magnitude ranges missed in an early time interval right after the occurrence of the main event.

In principle, *M*_*c*_ can be defined as a function of the mainshock magnitude and the elapsed time since the mainshock (see^76^). Parameter estimation of this time-dependent detection probability function could be incorporated directly into our Bayesian approach. Methods to fit the corresponding parameters of the detection function along with the ETAS parameters are proposed in^5,83^. However, this would be computationally too expensive in our Bayesian approach at this time and is deferred for future work.

### Robust estimation for the number of aftershock events

The conditional number of events, denoted as *N*(*x*, *y*, *m*|**θ***,***seq**, *M*_*l*_), in the spatial cell unit centered at (*x*, *y*) with magnitude greater than or equal to *m* in the forecasting interval [*T*_*start*_, *T*_*end*_] can be calculated as:

7 $$\begin{aligned} N\left( {\left. {x,y,m} \right|{{\varvec{\uptheta}}},{\mathbf{seq}},M_{l} } \right) &= \int\limits_{{T_{start} }}^{{T_{end} }} {\left( {\underbrace {{\mu \left( {x,y,m|{{\varvec{\uptheta}}},M_{l} } \right) + \lambda_{{{\text{ETAS}}}} \left( {\left. {t,x,y,m} \right|{{\varvec{\uptheta}}},{\mathbf{seq}},M_{l} } \right)}}_{{\lambda \left( {\left. {t,x,y,m} \right|{{\varvec{\uptheta}}},{\mathbf{seq}},M_{l} } \right)}}} \right)\,{\text{d}}t} = \int\limits_{{T_{start} }}^{{T_{end} }} {\lambda \left( {\left. {t,x,y,m} \right|{{\varvec{\uptheta}}},{\mathbf{seq}},M_{l} } \right)\,{\text{d}}t}\\ &= N_{b} (x,y,m|{{\varvec{\uptheta}}},M_{l} ) + \int\limits_{{T_{start} }}^{{T_{end} }} {\lambda_{{{\text{ETAS}}}} \left( {\left. {t,x,y,m} \right|{{\varvec{\uptheta}}},{\mathbf{seq}},M_{l} } \right){\text{d}}t}\\ \end{aligned}$$

where **seq** = {(*t*_*i*_, *x*_*i*_, *y*_*i*_, *m*_*i*_), *T*_o_ ≤ *t*_*i*_ < *T*_*start*_, *m*_*i*_ ≥ *M*_*l*_, *i* = 1:*N*_o_}; *t*_*i*_ is the arrival time for the *i*th event with magnitude *m*_*i*_ and location (*x*_*i*_, *y*_*i*_)$\in$ **A**; *µ*(*x*, *y*, *m*|**θ,*** M*_*l*_) is the time-invariant spatial rate representing the background seismicity of the area showing events with magnitude greater than or equal to *m* (*M* ≥ *m*) in the cell unit centered at (*x*, *y*)$\in$ **A**. Moreover, *µ*(*x*, *y*, *m*|**θ**, *M*_*l*_) can be expressed as the product of the “marker” *P*(*M* ≥ *m*|***θ***, *M*_*l*_) and the spatial (inhomogeneous) seismicity term *µ*(*x*, *y*|*M*_*l*_): $\mu \left(x,y,m\left|{\varvec{\uptheta}},{M}_{l}\right.\right)={e}^{-\beta \left(m-{M}_{l}\right)}\cdot \mu \left(x,y\left|{M}_{l}\right.\right)$. The time scale of *µ*(*x*, *y*|*M*_*l*_) is the same as *λ*_ETAS_ (e.g., daily or weekly). ${N}_{b}$ represents the average number of events occurred due to the background seismicity with *M* ≥ *m* in the forecasting interval [*T*_*start*_, *T*_*end*_], and estimated as ${N}_{b}\left(x,y,m\left|{{\varvec{\uptheta}},M}_{l}\right.\right)={e}^{-\beta \left(m-{M}_{l}\right)}\cdot \mu \left(x,y\left|{M}_{l}\right.\right)\cdot ({T}_{end}-{T}_{start})$. The term $\lambda \left(\cdot \right)=\mu \left(\cdot \right)+{\lambda }_{\mathrm{ETAS}}\left(\cdot \right)$ defines the total spatio-temporal conditional intensity function as the sum of time-invariant (spatial) background seismicity plus the ETAS seismicity rate. A *robust* estimate^26,28,53,54,84^ of the average number of events in the spatial cell unit centered at (*x*, *y*) with magnitude greater than or equal to *m* in the forecasting interval [*T*_*start*_, *T*_*end*_], denoted as ${\mathbb{E}}[N(x, y, m|\mathbf{s}\mathbf{e}\mathbf{q}, {M}_{l})]$, can be calculated over the domain of the model parameters Ω_**θ**_ as follows:

8 $$\begin{gathered} {\mathbb{E}}\left[ {N\left( {\left. {x,y,m} \right|{{\varvec{\uptheta}}},{\mathbf{seq}},M_{l} } \right)} \right] = \int\limits_{{\Omega_{{{\varvec{\uptheta}}}} }} {\left( {N_{b} \left( {\left. {x,y,m} \right|{{\varvec{\uptheta}}},M_{l} } \right) + \int\limits_{{T_{start} }}^{{T_{end} }} {\lambda_{{{\text{ETAS}}}} \left( {\left. {t,x,y,m} \right|{{\varvec{\uptheta}}},{\mathbf{seq}},M_{l} } \right){\text{d}}t\,} } \right) \cdot p\left( {{{\varvec{\uptheta}}}|{\mathbf{seq}},M_{l} } \right)\,{\text{d}}{{\varvec{\uptheta}}}} \hfill \\ \hfill \\ \end{gathered}$$

where ${\mathbb{E}}\left[\cdot \right]$ denotes the expectation, and *p*(**θ**|**seq**, *M*_*l*_) is the posterior conditional PDF for **θ** given the **seq** and *M*_*l*_. As mentioned above, **seq** denotes the sequence of events taking place before the beginning of the forecasting interval, i.e. [*T*_o_, *T*_*start*_). Nonetheless, the triggering effect of the events taking place during the forecasting interval [*T*_*start*_, *T*_*end*_] is expected to play a major role^28^. We denote this sequence as **seqg**, which is unknown at the time of forecasts and is simulated/generated herein. Let us assume that a plausible **seqg** is defined as the events within the forecasting interval so that **seqg** = {(*IAT*_*i*_, *x*_*i*_, *y*_*i*_, *m*_*i*_), *T*_*start*_ ≤ *t*_*i*_≤*T*_*end*_, *m*_*i*_≥*M*_*l*_}, where *IAT*_*i*_=*t*_*i*_–*t*_*i*-1_ stands for the inter-arrival time. The *robust* estimate for the average number of aftershock events in Eq. (8) should also consider all the plausible sequences of events **seqg** (i.e., the domain Ω_**seqg**_) that can happen during the forecasting time interval:

9 $$\begin{gathered} {\mathbb{E}}\left[ {N\left( {\left. {x,y,m} \right|{{\varvec{\uptheta}}},{\mathbf{seq}},M_{l} } \right)} \right] = \hfill \\ \int\limits_{{\Omega_{{{\varvec{\uptheta}}}} }} {\left[ {N_{b} \left( {\left. {x,y,m} \right|{{\varvec{\uptheta}}},M_{l} } \right) + \int\limits_{{\Omega_{{{\mathbf{seqg}}}} }} {\left( {\int\limits_{{T_{start} }}^{{T_{end} }} {\lambda_{{{\text{ETAS}}}} \left( {\left. {t,x,y,m} \right|{\mathbf{seqg}},{{\varvec{\uptheta}}},{\mathbf{seq}},M_{l} } \right){\text{d}}t} } \right)\, \cdot p\left( {{\mathbf{seqg}}|{{\varvec{\uptheta}}},{\mathbf{seq}},M_{l} } \right)\,{\text{d}}{\mathbf{seqg}}\,} } \right]} \, \cdot p\left( {{{\varvec{\uptheta}}}|{\mathbf{seq}},M_{l} } \right)\,\,{\text{d}}{{\varvec{\uptheta}}}\, \hfill \\ \end{gathered}$$

where *λ*_ETAS_(*t*, *x*, *y*, *m*|**seqg**, **θ**, **seq**, *M*_*l*_) is the space–time clustering ETAS model considering also the sequence of events taking place within the forecasting interval denoted as **seqg**; *p*(**seqg**|**θ**, **seq**, *M*_*l*_) is the PDF for the generated sequence **seqg** given that **θ** and **seq** are known. The integral with respect to time in Eq. (9) is calculated piece-wise by summing over the [*t*_*i*-1_, *t*_*i*_] sub-intervals to span the entire interval [*T*_*start*_, *T*_*end*_]:

10 $$\begin{aligned} \int_{{t_{i - 1} }}^{{t_{i} }} {\lambda_{{{\text{ETAS}}}} \left( {t,x,y,m|{\mathbf{seqg}}_{i - 1} ,{{\varvec{\uptheta}}},{\mathbf{seq}},M_{l} } \right){\text{d}}t} & = e^{{ - \beta \,\left( {m - M_{l} } \right)}} \sum\limits_{{j:\,\,t_{j} < t_{i} }} {K\,e^{{\alpha \left( {m_{j} - M_{l} } \right)}} \cdot K_{t} \int_{{t_{i - 1} }}^{{t_{i} }} {\frac{{\,{\text{d}}t\,\,}}{{\left( {t - t_{j} + c} \right)^{p} }}} \cdot \frac{{K_{r} }}{{\left( {r_{j}^{2} + d^{ * 2} } \right)^{q} }}} \\ & = e^{{ - \beta \,\left( {m - M_{l} } \right)}} \sum\limits_{{j:\,\,t_{j} < t_{i} }} {K\,e^{{\alpha \left( {m_{j} - M_{l} } \right)}} \cdot K_{t} {\rm I}_{t} \,\left( {t_{i} ,t_{i - 1} ,t_{j} } \right) \cdot \frac{{K_{r} }}{{\left( {r_{j}^{2} + d^{ * 2} } \right)^{q} }}} \\ \end{aligned}$$

where **seqg**_*i*-1_ is the generated sequence up to the (*i*-1)th event, and the sequence of events that precede the *i*th generated event is {**seq**,**seqg**_*i*-1_}. The integral over time I_*t*_ is calculated as:

11 $${\rm I}_{t} \,\left( {t_{i} ,t_{i - 1} ,t_{j} } \right) = \int_{{t_{i - 1} }}^{{t_{i} }} {\frac{{\,{\text{d}}t\,\,}}{{\left( {t - t_{j} + c} \right)^{p} }}} = \frac{1}{1 - p}\left[ {\left( {t_{i} - t_{j} + c} \right)^{1 - p} - \left( {t_{i - 1} - t_{j} + c} \right)^{1 - p} } \right]\,;\,\,\,p > 1$$

The term “robust” herein implies that the set of all possible model parameters is used to estimate the conditional number of events *N*(*x*, *y*, *m*|**seq**, *M*_*l*_) rather than a single nominal model parameter. Equation (9) can be solved via a fully simulation-based framework. First, vector of model parameters **θ** are sampled from *p*(**θ**|**seq**, *M*_*l*_); then those samples are used to generate plausible sequences **seqg** taking place within the forecasting interval [*T*_*start*_, *T*_*end*_]. The sequence of events that precede *T*_*end*_ is {**seq**, **seqg**}, where **seq** remains unchanged (observed data) among plausible samples. In the next two sections, we are going to discuss how samples are drawn from the probability distributions *p*(**θ**|**seq**, *M*_*l*_) (“Sampling **θ** from the distribution *p*(**θ**|**seq**, *M*_*l*_)” section) and *p*(**seqg**|**θ**, **seq**, *M*_*l*_) (“Generating sequences according to *p*(**seqg**|**θ**, **seq**, *M*_*l*_)” section) through a simulation-based workflow.

The probability that an event exceeding a given magnitude level *m* occurs within the aftershock zone in the forecasting interval can be calculated as follows (see also^28,48^):

12 $$P\left( {M \ge m} \right) = 1 - \exp \left( { - {\mathbb{E}}\left[ {N\left( {\left. {M \ge m} \right|{{\varvec{\uptheta}}},{\mathbf{seq}},M_{l} } \right)} \right]} \right) = 1 - \exp \left( { - \iint\limits_{{x,\,y\, \in {\mathbf{A}}}} {{\mathbb{E}}\left[ {N\left( {\left. {x,y,m} \right|{{\varvec{\uptheta}}},{\mathbf{seq}},M_{l} } \right)} \right]{\text{d}}x\,{\text{d}}y\,}} \right)$$

where ${\mathbb{E}}\left[N\left(x,y,m|{\varvec{\uptheta}},\mathbf{s}\mathbf{e}\mathbf{q},{M}_{l}\right)\right]$ is calculated from Eq. (9).

#### Calculating *K*

The coefficient *K* in calculating *λ*_ETAS_ (see Eq. 2) is calibrated so that the number of events with magnitude greater than or equal to *M*_*l*_ taking place in time interval [*T*_o_ , *T*_*start*_) over the whole aftershock zone **A** is equal to observed number *N*_o_ based on **seq** (see also^28^):

13 $$\int\limits_{{T_{{\text{o}}} }}^{{T_{start} }} {\iint\limits_{{\left( {x,y} \right)\, \in {\mathbf{A}}}} {\lambda \left( {\left. {t,x,y} \right|{{\varvec{\uptheta}}},{\mathbf{seq}},M_{l} } \right){\text{d}}x\,{\text{d}}y\,}{\text{d}}t} \, = N_{{\text{o}}}$$

where the term *λ*(*t*,*x*,*y*|**θ**,**seq**_*t*_,*M*_*l*_) is the conditional rate, and according to Eq. (7), we have:

14 $$\begin{aligned} \int\limits_{{T_{{\text{o}}} }}^{{T_{start} }} {\iint\limits_{{x,\,y\, \in {\mathbf{A}}}} {\lambda \left( {t,x,y|{{\varvec{\uptheta}}},{\mathbf{seq}}} \right){\text{d}}x\,{\text{d}}y\,{\text{d}}t}} &= \int\limits_{{T_{{\text{o}}} }}^{{T_{start} }} {\iint\limits_{{x,\,y\, \in {\mathbf{A}}}} {\left[ {\mu \left( {x,y|M_{l} } \right) + \lambda_{{{\text{ETAS}}}} \left( {\left. {t,x,y} \right|{{\varvec{\uptheta}}},{\mathbf{seq}},M_{l} } \right)} \right]{\text{d}}x\,{\text{d}}y\,{\text{d}}t}}\\&= N_{{b{\text{o}}}} + \int\limits_{{T_{{\text{o}}} }}^{{T_{start} }} {\iint\limits_{{x,\,y\, \in {\mathbf{A}}}} {\lambda_{{{\text{ETAS}}}} \left( {\left. {t,x,y} \right|{{\varvec{\uptheta}}},{\mathbf{seq}},M_{l} } \right){\text{d}}x\,{\text{d}}y\,{\text{d}}t}} \\ \end{aligned}$$

where *N*_*b*o_ is the number of events due to the background rate *µ*(*x*, *y*|*M*_*l*_) with magnitude greater than or equal to *M*_*l*_ taking place in time interval [*T*_o_ , *T*_*start*_) over the whole aftershock zone **A**. The second (triple) integral term in Eq. (14) is calculated piece-wise by summing over the sub-intervals [*t*_*i*-1_, *t*_*i*_] (where *i* = 2:*N*_o_) and the last interval [${t}_{{N}_{\mathrm{o}}}$, *T*_*start*_] (where ${t}_{{N}_{\mathrm{o}}}$ is the arrival time of the event *N*_o_) as follows (see also Eq. (10) and Eq. (11); *λ*_ETAS_ = 0 in the interval [*T*_o_ , *t*_1_]):

15 $$\begin{gathered} \int\limits_{{T_{{\text{o}}} }}^{{T_{start} }} {\iint\limits_{{x,\,y\, \in {\mathbf{A}}}} {\lambda_{{{\text{ETAS}}}} \left( {\left. {t,x,y} \right|{\mathbf{seq}},M_{l} } \right){\text{d}}x\,{\text{d}}y\,{\text{d}}t}} = \sum\limits_{i = 2}^{{N_{{\text{o}}} }} {\int_{{t_{i - 1} }}^{{t_{i} }} {\iint\limits_{{x,y \in {\mathbf{A}}}} {\lambda_{{{\text{ETAS}}}} \left( {t,x,y|{{\varvec{\uptheta}}},{\mathbf{seq}},M_{l} } \right){\text{d}}t}} } + \int_{{t_{{N_{{\text{o}}} }} }}^{{T_{start} }} {\iint\limits_{{x,y \in {\mathbf{A}}}} {\lambda_{{{\text{ETAS}}}} \left( {t,x,y|{{\varvec{\uptheta}}},{\mathbf{seq}},M_{l} } \right){\text{d}}t}} \hfill \\ = \sum\limits_{i = 2}^{{N_{{\text{o}}} }} {\,\sum\limits_{{j:\,\,t_{j} < t_{i} }} {Ke^{{\alpha \left( {m_{j} - M_{l} } \right)}} \cdot K_{t} {\rm I}_{t} \,\left( {t_{i} ,t_{i - 1} ,t_{j} } \right) \cdot K_{r} {\rm I}_{r} \left( {x_{j} ,y_{j} } \right)} } + \,\sum\limits_{{j:\,\,t_{j} < T_{start} }} {\,Ke^{{\alpha \left( {m_{j} - M_{l} } \right)}} \cdot K_{t} \,{\rm I}_{t} \,\left( {T_{start} ,t_{{N_{{\text{o}}} }} ,t_{j} } \right) \cdot K_{r} {\rm I}_{r} \left( {x_{j} ,y_{j} } \right)\,} \hfill \\ \end{gathered}$$

where,

16 $${\rm I}_{r} \left( {x_{j} ,y_{j} } \right) = \iint\limits_{{x,y \in {\mathbf{A}}}} {\frac{{{\text{d}}x\,{\text{d}}y}}{{\left( {r_{j}^{2} + d^{ * 2} } \right)^{q} }}}$$

Therefore, using Eq. (14) and Eq. (15), *K* can be derived as follows:

17 $$K = \frac{1}{{K_{t} \,K_{r} }} \cdot \frac{{N_{{\text{o}}} - N_{{b{\text{o}}}} }}{{\sum\limits_{i = 2}^{{N_{{\text{o}}} }} {\,\sum\limits_{{j:\,\,t_{j} < t_{i} }} {e^{{\alpha \left( {m_{j} - M_{l} } \right)}} \,{\rm I}_{t} \,\left( {t_{i} ,t_{i - 1} ,t_{j} } \right)\,{\rm I}_{r} \left( {x_{j} ,y_{j} } \right)} } + \,\sum\limits_{{j:\,\,t_{j} < T_{start} }} {\,e^{{\alpha \left( {m_{j} - M_{l} } \right)}} \,{\rm I}_{t} \,\left( {T_{start} ,t_{{N_{{\text{o}}} }} ,t_{j} } \right)\,{\rm I}_{r} \left( {x_{j} ,y_{j} } \right)\,} }}$$

The integral over the whole aftershock zone **A**, denoted as ${\mathrm{I}}_{r}$ in Eq. (16), should be solved numerically. However, ${\mathrm{I}}_{r}$ can be *approximated* with integration over infinite space (denoted as ${\widetilde{\mathrm{I}}}_{r}$); thus, it can be shown (see Eq. 4) that ${K}_{r}{\widetilde{\mathrm{I}}}_{r}=1$. Schoenberg^71^ has shown that the assumption of an infinite spatial domain has negligible effect and so this approximation can be used to reduce the computational cost of evaluating the triple integral (double integral on spatial domain and another integral on time domain). This formal assumption was employed in many research efforts (e.g.,^28,50^). However, this approximation may cause significant errors when the considered aftershock zone **A** is not large enough to satisfy Eq. (4). Parameter *K* can be inferred based on two different approaches:

- *Calculate K*: The parameter *K* has a derived distribution based on Eq. (17) as a function of other ETAS model parameters. That is, the vector of model parameters **θ** = [*β*, *α*, *c*, *p*, *d*, *q*] has six parameters considering simple spatial kernel density, and the vector **θ** = [*β*, *α*, *c*, *p*, *d*, *q*, *γ*] has seven parameters employing the magnitude-dependent spatial kernel density*.*
- *Learn K*: Parameter *K* is learned within the Bayesian updating framework. In this approach, **θ** = [*β*, *K*, *α*, *c*, *p*, *d*, *q*] using the simple spatial kernel density, **θ** = [*β*, *K*, *α*, *c*, *p*, *d*, *q*, *γ*] employing the magnitude-dependent spatial kernel density.

Both methods for characterizing *K* are examined and compared herein for the case-study (see Sect. Sensitivity analyses, Results).

### Sampling **θ** from the distribution *p*(**θ**|**seq**, *M*_*l*_)

The probability distribution *p*(**θ**|**seq**, *M*_*l*_) is calculated using Bayesian parameter estimation (for more detail see^28^):

18 $$\underbrace {{p\left( {\left. {{\varvec{\uptheta}}} \right|{\mathbf{seq}},M_{l} } \right)}}_{{{\text{posterior}}}} = \frac{{p\left( {\left. {{\mathbf{seq}}} \right|{{\varvec{\uptheta}}},M_{l} } \right)p\left( {\left. {{\varvec{\uptheta}}} \right|M_{l} } \right)}}{{\int\limits_{{\Omega_{{{\varvec{\uptheta}}}} }} {p\left( {\left. {{\mathbf{seq}}} \right|{{\varvec{\uptheta}}},M_{l} } \right)\,p\left( {\left. {{\varvec{\uptheta}}} \right|M_{l} } \right){\text{d}}{{\varvec{\uptheta}}}} }} = C^{ - 1} \underbrace {{p\left( {\left. {{\mathbf{seq}}} \right|{{\varvec{\uptheta}}},M_{l} } \right)}}_{{{\text{likelihood}}}}\,\,\underbrace {{p\left( {\left. {{\varvec{\uptheta}}} \right|M_{l} } \right)}}_{{{\text{prior}}}}$$

where *C*^−1^ is a normalizing constant; *p*(**seq**|**θ**, *M*_*l*_) is the likelihood of the observed sequence given the vector of model parameters **θ** and lower cut-off magnitude *M*_*l*_, *p*(**θ**|*M*_*l*_) is the prior distribution for the vector ***θ***. The prior joint distribution *p*(**θ**|*M*_*l*_) can be estimated as the product of marginal lognormal PDFs for each model parameter (i.e., a multivariate lognormal distribution with zero correlation between the pairs of model parameters **θ**) whose central statistics (median) can be assigned based on the regional models; thus,

19 $$p\left( {\left. {{\varvec{\uptheta}}} \right|M_{l} } \right) = \frac{1}{{\left( {\prod\limits_{k = 1}^{n} {\theta_{k} } } \right)\sqrt {\left( {2\pi } \right)^{n} \left| {\mathbf{S}} \right|} }}\exp \left( { - \frac{1}{2}\left( {\ln {{\varvec{\uptheta}}} - {{\varvec{\upmu}}}_{{\ln {{\varvec{\uptheta}}}}} } \right)^{{\text{T}}} {\mathbf{S}}^{ - 1} \left( {\ln {{\varvec{\uptheta}}} - {{\varvec{\upmu}}}_{{\ln {{\varvec{\uptheta}}}}} } \right)} \right)$$

where *n* is the number of uncertain parameters in the vector **θ** ={**θ**_*k*_, *k* = 1:*n}*; µ_ln**θ**_ is the vector of the mean value of ln**θ** (= logarithm of the vector of median values) associated with the prior distribution; **S** is the covariance matrix. In order to sample from the posterior distribution *p*(**θ**|**seq**, *M*_*l*_) in Eq. (18), Markov Chain Monte Carlo (MCMC) simulation routine is employed. MCMC is particularly useful for drawing samples from the target posterior PDF *p*(**θ**|**seq**, *M*_*l*_), while it is known up to a scaling constant *C*^−1^ (see^54^). Although Eq. (18) looks daunting, we only need un-normalized PDFs in MCMC, as discussed in *Supplementary Information* (Section SI-1-Method). The MCMC routine here employs the Metropolis–Hastings (MH) algorithm^85,86^ in order to generate samples from the target probability distribution *p*(**θ**|**seq**, *M*_*l*_), and later to estimate the robust estimation based on Eq. (9). The MH algorithm generates a Markov chain that produces a sequence of samples [**θ**_1_ $\to$ **θ**_2_ $\to \cdots \to$ **θ**_*i*_ $\to \cdots$], where **θ**_*i*_ represents the state of Markov chain at *i*th iteration (the first few samples are often discarded to reduce the initial transient effect). It can be shown that the samples from the chain after the initial transient ones reflect samples from the target posterior distribution *p*(**θ**|**seq**, *M*_*l*_). To improve the rate of convergence of the simulation process, we used an adaptive MH algorithm (as proposed in^54^) herein that introduces a sequence of intermediate candidate evolutionary PDF’s that resemble more and more the target PDF. The Metropolis–Hastings routine and its adaptive version are described in detail in Section SI-1-Method.

#### Calculating the likelihood of the observed sequence *p*(**seq**|**θ**, *M*_*l*_)

**Figure 11:**
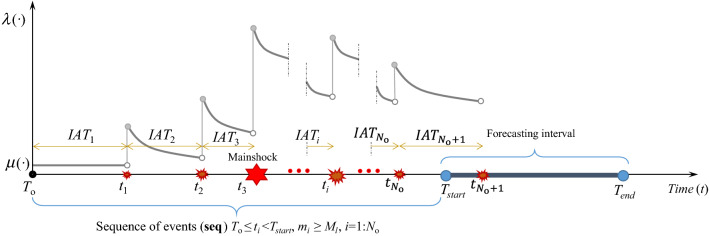
The main parameters required for constructing the likelihood function; the seismicity rate as a function of time showing how each event provokes a jump and is followed by a decay; and the illustration of **seq**.

Figure 11 shows the observed sequence, **seq** = {(*t*_*i*_, *x*_*i*_, *y*_*i*_, *m*_*i*_), *T*_o_ ≤ *t*_*i*_ < *T*_*start*_, *m*_*i*_ ≥ *M*_*l*_, *i* = 1:*N*_o_}, with *N*_o_ events where *i* = 1 is assigned to the first event (the schematic plot in Fig. 11 shows only the time scale). The likelihood of observing the sequence can be calculated as follows:

20 $$\begin{gathered} p\left( {{\mathbf{seq}}|{{\varvec{\uptheta}}},M_{l} } \right) = \left( {\prod\limits_{i = 1}^{{N_{{\text{o}}} }} {p\left( {IAT_{i} ,x_{i} ,y_{i} ,m_{i} \left| {{{\varvec{\uptheta}}},{\mathbf{seq}},M_{l} } \right.} \right)} } \right) \cdot P\left( {IAT_{{N_{{\text{o}}} + 1}} \ge T_{start} - t_{{N_{{\text{o}}} }} \left| {{{\varvec{\uptheta}}},{\mathbf{seq}},M_{l} } \right.} \right) \hfill \\ \,\,\,\,\,\,\,\,\,\,\,\,\,\,\,\,\,\,\,\,\,\,\,\,\,\,\,\,\,\,\, = \left( {\prod\limits_{i = 1}^{{N_{{\text{o}}} }} {p\left( {m_{i} \left| {{{\varvec{\uptheta}}},M_{l} } \right.} \right)} } \right) \cdot \left( {\prod\limits_{i = 1}^{{N_{{\text{o}}} }} {p\left( {IAT_{i} ,x_{i} ,y_{i} \left| {{{\varvec{\uptheta}}},{\mathbf{seq}},M_{l} } \right.} \right)} } \right) \cdot P\left( {IAT_{{N_{{\text{o}}} + 1}} \ge T_{start} - t_{{N_{{\text{o}}} }} \left| {{{\varvec{\uptheta}}},{\mathbf{seq}},M_{l} } \right.} \right) \hfill \\ \end{gathered}$$

where *p*(*IAT*_*i*_, *x*_*i*_, *y*_*i*_, *m*_*i*_|**θ**, **seq**, *M*_*l*_) is the conditional probability of observing event *i* within the **seq** with inter-arrival time *IAT*_*i*_=*t*_*i*_–*t*_*i*-1_, magnitude equal to *m*_*i*_ and epicenter location (*x*_*i*_, *y*_*i*_)$\in$ **A**. The CCDF *P*(${IAT}_{{N}_{\mathrm{o}}+1}\ge {T}_{start}-{t}_{{N}_{\mathrm{o}}}$|**θ**, **seq**, *M*_*l*_) is the conditional probability that the (*N*_o_ + 1)th event takes place out of the time window [${t}_{{N}_{\mathrm{o}}}$, *T*_*start*_), where *t*_*N*o_ is the arrival time of the *N*_o_th event (see Fig. 11). As shown in Eq. (6), the probability of observing an event with magnitude *m*_*i*_, denoted as *p*(*m*_*i*_|**θ**, *M*_*l*_), is history-independent. Thus, we can de-couple the probability of observing the magnitude *m*_*i*_, *p*(*m*_*i*_|**θ**, *M*_*l*_) and the conditional probability *p*(*IAT*_*i*_, *x*_*i*_, *y*_*i*_|**θ**, **seq**, *M*_*l*_ ) as shown in Eq. (20). In Fig. 11, the total conditional time- and history-dependent intensity function *λ*(*·*), as derived in Eq. (6), is shown with gray colored curves. Visibly, the value of *λ*(*·*) shows a spike right after the occurrence of an event and decays within the inter-arrival time to the next event. In the time interval *IAT*_1_ (= *t*_1_-*T*_o_), the seismicity rate is constant and equal to the time-invariant spatial background seismicity *µ*(*x*, *y*|*M*_*l*_) as shown in Fig. 11 (before *T*_o_, we consider only background seismicity; see also “Robust estimation for the number of aftershock events” section). We assume an exponential distribution for probability distribution of *IAT*_*i*_ consistent with a non-homogeneous Poisson point process. Thus, Eq. (20) can be written as:

21 $$\begin{gathered} p\left( {{\mathbf{seq}}|{{\varvec{\uptheta}}},M_{l} } \right) = \left( {\prod\limits_{i = 1}^{{N_{{\text{o}}} }} {\beta e^{{ - \beta \,\left( {m_{i} - M_{l} } \right)}} } } \right) \cdot \left( {\mu \left( {x_{1} ,y_{1} |M_{l} } \right) \cdot e^{{ - \int_{{T_{{\text{o}}} }}^{{t_{1} }} {\iint\limits_{{x,y\, \in {\mathbf{A}}}} {\mu \left( {x,y|M_{l} } \right)\,{\text{d}}x\,{\text{d}}y\,{\text{d}}t}} }} } \right) \cdot \,\,\,\,\, \hfill \\ \,\,\,\,\,\,\,\,\,\,\,\,\,\,\,\,\,\,\,\,\,\,\,\,\,\,\,\,\,\,\,\,\,\,\, \cdot \left( {\prod\limits_{i = 2}^{{N_{{\text{o}}} }} {\lambda \left( {t_{i} ,x_{i} ,y_{i} |{{\varvec{\uptheta}}},{\mathbf{seq}},M_{l} } \right) \cdot e^{{ - \int_{{t_{i - 1} }}^{{t_{i} }} {\iint\limits_{{x,y\, \in {\mathbf{A}}}} {\lambda \left( {t,x,y|{{\varvec{\uptheta}}},{\mathbf{seq}}} \right)\,{\text{d}}x\,{\text{d}}y\,{\text{d}}t}} }} } } \right)\, \cdot \,e^{{ - \int\limits_{{t_{{N_{{\text{o}}} }} }}^{{T_{start} }} {\iint\limits_{{x,y \in {\mathbf{A}}}} {\lambda \left( {t,x,y|{{\varvec{\uptheta}}},{\mathbf{seq}}} \right)\,{\text{d}}x\,{\text{d}}y\,{\text{d}}t}} }} \hfill \\ \end{gathered}$$

Equation (21) is derived considering that *λ*(*t*_1_, *x*_1_, *y*_1_|**θ**, **seq**, *M*_*l*_) = *µ*(*x*_1_, *y*_1_|*M*_*l*_) in the time interval [*T*_o_, *t*_1_], and that *P*(${IAT}_{{N}_{\mathrm{o}}+1}\ge {T}_{start}-{t}_{{N}_{\mathrm{o}}}$|**θ**, **seq**, *M*_*l*_) is the CCDF of the exponential distribution of ${IAT}_{{N}_{\mathrm{o}}+1}$. The *likelihood function* can be expressed as follows by combining the exponential terms in Eq. (21):

22 $$p\left( {{\mathbf{seq}}|{{\varvec{\uptheta}}},M_{l} } \right) = \,\mu \left( {x_{1} ,y_{1} |M_{l} } \right) \cdot \left( {\prod\limits_{i = 1}^{{N_{{\text{o}}} }} {\beta e^{{ - \beta \,\left( {m_{i} - M_{l} } \right)}} } } \right) \cdot \left( {\prod\limits_{i = 2}^{{N_{{\text{o}}} }} {\lambda \left( {t_{i} ,x_{i} ,y_{i} |{{\varvec{\uptheta}}},{\mathbf{seq}},M_{l} } \right)} } \right) \cdot e^{{ - \int\limits_{{T_{{\text{o}}} }}^{{T_{start} }} {\iint\limits_{{x,\,y\, \in {\mathbf{A}}}} {\lambda \left( {\left. {t,x,y} \right|{{\varvec{\uptheta}}},{\mathbf{seq}},M_{l} } \right)\,{\text{d}}x\,{\text{d}}y\,{\text{d}}t}} }}$$

where,

23 $$\lambda \left( {t_{i} ,x_{i} ,y_{i} |{{\varvec{\uptheta}}},{\mathbf{seq}},M_{l} } \right) = \mu \left( {x_{i} ,y_{i} |M_{l} } \right) + \sum\limits_{{j:\,\,t_{j} < t_{i} }} {K\,e^{{\alpha \left( {m_{j} - M_{l} } \right)}} \cdot \frac{{\,K_{t} \,\,}}{{\left( {t_{i} - t_{j} + c} \right)^{p} }} \cdot \frac{{K_{r} }}{{\left( {r_{ji}^{2} + d^{ * 2} } \right)^{q} }}}$$

where *r*_*ji*_ indicates the distance of the *i*th event with respect to the previously occurred *j*th event (*t*_*j*_ < *t*_*i*_). With reference to the Bayes formula in Eq. (18), the term *µ*(*x*_1_, *y*_1_|*M*_*l*_) is independent of any realization of the vector of model parameters **θ**; hence, this value can be eliminated from both nominator and denominator. Moreover, the MH algorithm employs the ratio of the likelihoods (see Section SI-1-Method), and constant terms can easily be disregarded. Thus, an estimator for the likelihood can be defined as follows (within the context of Bayesian inference):

24 $$p\left( {{\mathbf{seq}}|{{\varvec{\uptheta}}},M_{l} } \right) \approx \,\left( {\prod\limits_{i = 1}^{{N_{{\text{o}}} }} {\beta e^{{ - \beta \,\left( {m_{i} - M_{l} } \right)}} } } \right) \cdot \left( {\prod\limits_{i = 2}^{{N_{{\text{o}}} }} {\lambda \left( {t_{i} ,x_{i} ,y_{i} |{{\varvec{\uptheta}}},{\mathbf{seq}},M_{l} } \right)} } \right) \cdot e^{{ - \int\limits_{{T_{{\text{o}}} }}^{{T_{start} }} {\iint\limits_{{x,\,y\, \in {\mathbf{A}}}} {\lambda \left( {\left. {t,x,y} \right|{{\varvec{\uptheta}}},{\mathbf{seq}},M_{l} } \right)\,{\text{d}}x\,{\text{d}}y\,{\text{d}}t}} }}$$

With reference to “Calculating *K*” section, we have two approaches to follow:

- In case that we want to calculate parameter *K*, as noted in “Calculating *K*” section (*Calculate K*), the power of the exponential term in Eq. (24) becomes *N*_o_ (see Eq. 13); thus, the constant term ${e}^{{-N}_{\mathrm{o}}}$ can also be eliminated while employing the Bayesian updating framework in Eq. (18) (see also Section SI-1-Method). In this way, the likelihood estimator in Eq. (24) can be simplified as the product of the probabilities of observing *m*_*i*_, for *i* = 1:*N*_o_, and those of the total rate *λ*(*t*_*i*_, *x*_*i*_, *y*_*i*_|***θ***, **seq**_*t*_, *M*_*l*_) for *i* = 2:*N*_o_.
- If we learn parameter *K* within the MH algorithm (see Calculating *K*” section, *Learn K*), the triple integral (the power of the exponential term) in Eq. (24) can be solved based on Eq. (14) and Eq. (15). Even in this case, we can exclude the constant term ${e}^{{-N}_{b\mathrm{o}}}$ from the likelihood estimator (as it is independent of any realization of ***θ***). Thus, Eq. (15) is employed to estimate the triple integral of λ_ETAS_ over the time interval [*T*_o_ , *T*_*start*_]. As shown in Eq. (15), it is solved piece-wise by summing over the sub-intervals [*t*_*i*-1_, *t*_*i*_] (where *i* = 2:*N*_o_), and [*t*_*N*o_, *T*_*start*_].

### Generating sequences according to *p*(**seqg**|**θ**, **seq**, *M*_*l*_)

This section describes how the catalogue of stochastic events **seqg**, which will occur during the forecasting interval, is simulated. The event *i* in the generated catalogue is identified by the arrival time *t*_*i*_ = *t*_*i*-1_ + *IAT*_*i*_, the Cartesian coordinates (*x*_*i*_, *y*_*i*_) of the epicenter, and the magnitude *m*_*i*_. The sequence of events in the generated catalogue up to event *i* is denoted by **seqg**_*i*-1_. Thus, **seqg**_*i*_={**seqg**_*i*-1_, (*IAT*_*i*_, *x*_*i*_, *y*_*i*_, *m*_*i*_)}. Catalogue simulation continues until the arrival time of the simulated events do not fall outside the forecasting interval (i.e., *t*_*i*_ ≤ *T*_*end*_). The likelihood *p*(**seqg**|**θ**, **seq**, *M*_*l*_) for the generated catalogue **seqg** can be calculated using the rule of product in probability; that is conditioning the occurrence of *i*th event on the previous *i-*1 events:

25 $$p\left( {{\mathbf{seqg}}|{{\varvec{\uptheta}}},{\mathbf{seq}},M_{l} } \right) = \prod\limits_{i} {p\left( {IAT_{i} ,x_{i} ,y_{i} ,m_{i} |{\mathbf{seqg}}_{i - 1} ,{{\varvec{\uptheta}}},{\mathbf{seq}},M_{l} } \right)}$$

where *p*(*IAT*_*i*_, *x*_*i*_, *y*_*i*_, *m*_*i*_|**seqg**_*i*-1_, **θ**, **seq**, *M*_*l*_) is the conditional probability of observing event *i*. It is noted that the sequence of events that precede the *i*th generated event is {**seq**, **seqg**_*i*-1_}. For event *i* = 1 in Eq. (25), **seqg**_*i*-1_** = seqg**_0_ = $\varnothing$. The probability distribution *p*(*IAT*_*i*_, *x*_*i*_, *y*_*i*_, *m*_*i*_|**seqg**_*i*-1_, **θ**, **seq**, *M*_*l*_) can be further expanded (again using the probability product rule) as follows:

26 $$\begin{gathered} p\left( {IAT_{i} ,x_{i} ,y_{i} ,m_{i} |{\mathbf{seqg}}_{i - 1} ,{{\varvec{\uptheta}}},{\mathbf{seq}},M_{l} } \right) = \hfill \\ p\left( {m_{i} |{{\varvec{\uptheta}}},M_{l} } \right) \cdot p\left( {IAT_{i} |m_{i} ,{\mathbf{seqg}}_{i - 1} ,{{\varvec{\uptheta}}},{\mathbf{seq}},M_{l} } \right) \cdot p\left( {x_{i} ,y_{i} |IAT_{i} ,m_{i} ,{\mathbf{seqg}}_{i - 1} ,{{\varvec{\uptheta}}},{\mathbf{seq}},M_{l} } \right) \hfill \\ \end{gathered}$$

where *p*(*m*_*i*_|**θ**, *M*_*l*_) is the conditional marginal PDF for the magnitude *m*_*i*_ given the model parameters **θ**, and *M*_*l*_; *p*(*IAT*_*i*_|*m*_*i*_, **seqg**_*i*-1_, **θ**, **seq**, *M*_*l*_) is the conditional marginal PDF for inter-arrival time *IAT*_*i*_ given the history of past events {**seq**, **seqg**_*i*-1_} and given that the value of magnitude is equal to *m*_*i*_; finally, *p*(*x*_*i*_, *y*_*i*_|*IAT*_*i*_, *m*_*i*_, **seqg**_*i*-1_, **θ**, **seq**, *M*_*l*_) is the conditional joint PDF for the spatial position (*x*_*i*_, *y*_*i*_)$\in$ **A** given that *IAT*_*i*_ and *m*_i_ are known. The break-down into the product of several conditional PDFs is necessary as we need to sample *m*_*i*_, *IAT*_*i*_, and (*x*_*i*_, *y*_*i*_) directly from the three consecutive probability terms, respectively. To generate a plausible sequence of events during the forecasting interval [*T*_*start*_, *T*_*end*_], the procedure, illustrated originally in^28^, is adopted with some modifications herein and described in the following sections.

#### Sampling magnitude, *m*

The magnitude *m*_*i*_ of the *i*th event within **seqg** is simulated according to a truncated Exponential PDF with rate β, denoted as *p*(*M* = *m*_*i*_|**θ**, *M*_*l*_) in Eq. (6). The Exponential cumulative density function (CDF) used to generate *m* has the upper bound magnitude threshold *M*_max_:

27 $$P\left( {M \le m\left| {M_{l} \le M \le M_{\max } } \right.} \right) = \frac{{1 - e^{{ - \beta \,\left( {m - M_{l} } \right)}} }}{{1 - e^{{ - \beta \,\left( {M_{\max } - M_{l} } \right)}} }}$$

To sample from this CDF, generate a uniform random number *u* $\sim$ Uniform (0, 1):

28 $$m_{i} = \frac{ - 1}{\beta } \cdot \ln \left[ {1 - u \cdot \left( {1 - e^{{ - \beta \left( {M_{\max } - M_{l} } \right)}} } \right)} \right] + M_{l}$$

If the upper bound threshold ${M}_{\mathrm{max}}$ is not considered (${M}_{\mathrm{max}}\to +\infty$), Eq. (28) can be modified by eliminating the term $1-\mathrm{exp}(-\beta ({M}_{\mathrm{max}}-{M}_{l}))$ in the parenthesis. It is to note that we have considered the upper-bound threshold ${M}_{\mathrm{max}}$ just for generating the sequences within **seqg**. The ETAS rate (see Eqs. 2 and 6) has no magnitude upper threshold.

#### Sampling the inter-arrival time, *IAT*

To generate the inter-arrival time of the *i*th event, *IAT*_*i*_, within **seqg** (given that its magnitude *m*_*i*_ is already known), the Thinning algorithm^87^ (see also^57^) is employed herein. The thinning algorithm is based on simulating inter-arrival times from a homogeneous Poisson process with sufficiently large intensity, denoted here as λ_max_, and then thinning out (filtering) the points according to the conditional intensity function denoted as λ_gen_. The Thinning algorithm is as follows:

*Step (1)*: Calculate the temporal Poisson rate λ_max_ over the entire aftershock zone at time *t*_*i*-1_ having integrated out the spatial term, as follows (see Eq. 7):

29 $$\begin{aligned} \lambda_{\max } & = \iint\limits_{{x,y \in {\mathbf{A}}}} {\lambda \left( {t_{i - 1} ,x,y|{\mathbf{seqg}}_{i - 1} ,{{\varvec{\uptheta}}},{\mathbf{seq}},M_{l} } \right){\text{d}}x{\text{d}}y} = \iint\limits_{{x,y \in {\mathbf{A}}}} {\left( {\mu \left( {\left. {x,y} \right|M_{l} } \right) + \lambda_{{{\text{ETAS}}}} \left( {t_{i - 1} ,x,y|{\mathbf{seqg}}_{i - 1} ,{{\varvec{\uptheta}}},{\mathbf{seq}},M_{l} } \right)} \right){\text{d}}x{\text{d}}y} \\ & = \mu_{b} + \sum\limits_{{j:\,t_{j} < t_{i - 1} }} {K\,e^{{\alpha \left( {m_{j} - M_{l} } \right)}} \cdot \frac{{\,K_{t} \,\,}}{{\left( {t_{i - 1} - t_{j} + c} \right)^{p} }} \cdot K_{r} \underbrace {{\left(\,\, {\iint\limits_{{x,y \in {\mathbf{A}}}} {\frac{1}{{\left( {r_{j}^{2} + d^{ * 2} } \right)^{q} }}{\text{d}}x{\text{d}}y}} \right)}}_{{{\rm I}_{r} \left( {x_{j} ,y_{j} } \right)}}} = \mu_{b} + \sum\limits_{{j:\,t_{j} < t_{i - 1} }} {\frac{{\,KK_{t} K_{r} \cdot \,e^{{\alpha \left( {m_{j} - M_{l} } \right)}} \cdot {\rm I}_{r} \left( {x_{j} ,y_{j} } \right)\,\,}}{{\left( {t_{i - 1} - t_{j} + c} \right)^{p} }}} \\ \end{aligned}$$

where *µ*_*b*_ is the background rate over the whole area **A**. It is noted that the ETAS rate is estimated by summing up the rate of events taken place before *t*_*i*-1_. For generating the first event, *t*_*i*-1_ is set to *T*_*start*_.

*Step (2)*: Generate the inter-arrival time *IAT*_gen_ from a homogeneous Exponential CDF with the rate *λ*_max_ expressed as *F*(*IAT*) = 1-exp(-*λ*_max_·*IAT*). Since the inverse function *F*(*IAT*)^−1^ has a closed-from expression, we use the inverse transform sampling technique. To sample from this CDF, first generate a uniform random number *u* ~ Uniform (0, 1); the generated *IAT*_gen_ can analytically be drawn as *IAT*_gen_ = -ln(1-*u*)/ *λ*_max_.

*Step (3)*: Calculate the Poisson rate at time *t*_gen_ = *t*_*i*-1_ + *IAT*_gen_, denoted by *λ*_gen_, from Eq. (29) by substituting *t*_*i*-1_ with *t*_gen_. It is noted that the generated magnitude *m*_*i*_ in the previous section has no effect on the rate *λ*_gen_, as this rate is calculated based on events taking place before *t*_gen_. The rate *λ*_gen_ should always be equal to or smaller than *λ*_max_; i.e., *λ*_gen_ ≤ *λ*_max_. If this condition does not hold, go to *Step* (5).

*Step (4)*: Accept *t*_gen_ with the probability *pr* = *λ*_gen_/*λ*_max_ by first generating a uniform random number *u* ~ Uniform (0, 1); if *u* ≤ *pr*, then the time *t*_gen_ is accepted, and thus *t*_*i*_ = *t*_gen_. The procedure continues by generating the next inter-arrival time until *t*_*i*_ > *T*_*end*_. *λ*_max_ is going to change while generating each new *t*_*i*_.

*Step (5)*: Reject *t*_gen_ if *u* > *pr* or *λ*_gen_ >* λ*_max_ (i.e., *pr* = *λ*_gen_/*λ*_max_ > 1). Let us denote the rejected *t*_gen_ as *t*_gen_^(-)^ (in order to keep track of it for the next simulation). In the case of rejection, the procedure continues by sampling a new inter-arrival time *IAT*_gen_ from the homogeneous Exponential PDF with rate *λ*_gen_. The new generated arrival time is calculated as *t*_gen_ = *t*_gen_^(-)^ + *IAT*_gen_, *t*_gen_ ≤ *T*_*end*_. The quantities *λ*_gen_ and *pr* are calculated again to test whether the newly generated inter-arrival time is accepted or rejected.

Based on the above discussion, it is noted that *p*(*IAT*_*i*_|*m*_*i*_, **seqg**_*i*-1_, **θ**, **seq**, *M*_*l*_) = *p*(*IAT*_*i*_|**seqg**_*i*-1_, **θ**, **seq**, *M*_*l*_); i.e., *m*_*i*_ is independent of *IAT*_*i*_.

#### Generating the epicentral coordinate, (*x*, *y*)

The spatial coordinates are simulated from a spatial kernel *p*(*x*, *y*|*IAT*_*i*_, *m*_*i*_, **seqg**_*i*-1_, **θ**, **seq**, *M*_*l*_) that is obtained based on the epicenters of the previous events (i.e., {**seq**, **seqg**_*i*-1_}), and conditioned on the generated *m*_*i*_ and *IAT*_*i*_. This spatial kernel is a joint PDF of the epicentral (Cartesian) coordinates, from which (*x*_*i*_, *y*_*i*_) for the *i*th event can be generated given that the magnitude *m*_*i*_, the time of occurrence *t*_*i*_, and the previous (*i*-1) events within the generated sequence **seqg**_*i*-1_ are known (see also^28^):

30 $$p\left( {x,y|IAT_{i} ,m_{i} ,{\mathbf{seqg}}_{i - 1} ,{{\varvec{\uptheta}}},{\mathbf{seq}},M_{l} } \right) = \frac{{p\left( {IAT_{i} ,x,y,m_{i} |{\mathbf{seqg}}_{i - 1} ,{{\varvec{\uptheta}}},{\mathbf{seq}},M_{l} } \right)}}{{\iint\limits_{{x,y \in {\mathbf{A}}}} {p\left( {IAT_{i} ,x,y,m_{i} |{\mathbf{seqg}}_{i - 1} ,{{\varvec{\uptheta}}},{\mathbf{seq}},M_{l} } \right){\text{d}}x{\text{d}}}y}}$$

In other words, the joint PDF *p*(*x*, *y*|·) in Eq. (30) is estimated for all Cartesian coordinates (*x*, *y*)$\in$ **A** and is normalized to obtain the kernel. The likelihood in the nominator *p*(*IAT*_*i*_, *x*, *y*, *m*_*i*_|**seqg**_*i*-1_, **θ**, **seq**, *M*_*l*_) of Eq. (30) can be calculated as (see also Eq. 20):

31 $$p\left( {IAT_{i} ,x,y,m_{i} |{\mathbf{seqg}}_{i - 1} ,{{\varvec{\uptheta}}},{\mathbf{seq}},M_{l} } \right) = \underbrace {{\beta e^{{ - \beta \,\left( {m_{i} - M_{l} } \right)}} }}_{{p\left( {m_{i} \left| {{{\varvec{\uptheta}}},M_{l} } \right.} \right)}} \cdot \underbrace {{\lambda \left( {\left. {t_{i} ,x,y} \right|{\mathbf{seqg}}_{i - 1} ,{{\varvec{\uptheta}}},{\mathbf{seq}},M_{l} } \right) \cdot e^{{ - \int_{{t_{i - 1} }}^{{t_{i} }} {\lambda \left( {t,x,y|{\mathbf{seqg}}_{i - 1} ,{{\varvec{\uptheta}}},{\mathbf{seq}},M_{l} } \right){\text{d}}t} }} }}_{{p\left( {IAT_{i} ,x,y\left| {{\mathbf{seqg}}_{i - 1} ,{{\varvec{\uptheta}}},{\mathbf{seq}},M_{l} } \right.} \right)}}$$

where *λ*(*t*_*i*_, *x*, *y*|**seqg**_*i*-1_, **θ**, **seq**, *M*_*l*_) is obtained for each Cartesian coordinate (*x*, *y*)$\in$ **A** and for all *t* < *t*_*i*_ (see Eq. 7 and Eq. 29) and the exponential power is derived as (see also Eq. (10) and Eq. (11)):

32 $$\int_{{t_{i - 1} }}^{{t_{i} }} {\lambda \left( {t_{i} ,x,y|{\mathbf{seqg}}_{i - 1} ,{{\varvec{\uptheta}}},{\mathbf{seq}},M_{l} } \right){\text{d}}t} = \mu \left( {x,y|M_{l} } \right) \cdot IAT_{i} + \sum\limits_{{j:\,\,t_{j} < t_{i} }} {K\,e^{{\alpha \left( {m_{j} - M_{l} } \right)}} \cdot K_{t} {\rm I}_{t} \,\left( {t_{i} ,t_{i - 1} ,t_{j} } \right) \cdot \frac{{K_{r} }}{{\left( {r_{j}^{2} + d^{ * 2} } \right)^{q} }}}$$

The denominator in Eq. (30) is the sum over all the Cartesian coordinate (*x*, *y*)$\in$ **A**. Moreover, the PDF of observing *m*_*i*_, i.e. $\beta \mathrm{exp}(-\beta ({m}_{i}-{M}_{l}))$, can be dropped from both nominator and denominator of Eq. (30) since it is not location-dependent. Thus, it is interesting to mention that *p*(*x*, *y*|·) in Eq. (30) is independent of *m*_*i*_.

### N-test based on the simulation-based Bayesian framework

The N-test is intended to measure (in a probabilistic manner) how well the forecasted number of earthquakes matches the observed number of events^59^. According to this test, we fit a Poisson distribution to the forecasted number of events *N*_fore_ with magnitude greater than or equal to ${M}_{l}$. *N*_fore_ is the expected number of events in the forecasting interval integrated over the aftershock zone **A**, as in Eq. (12), ${N}_{\mathrm{fore}}={\mathbb{E}}\left[N\left(M\ge {M}_{l}|{\varvec{\uptheta}},\mathbf{s}\mathbf{e}\mathbf{q}, {M}_{l}\right)\right]$. The objective of the N-test is to verify if the observed number of events *N*_obs_ (with $M\ge {M}_{l}$ taking place within the forecasting interval) are not outliers. To this end, one needs to estimate the two probabilities that the number of events $n\le {N}_{\text{obs}}$ and $n\ge {N}_{\text{obs}}$ given ${N}_{\text{fore}}$, which are denoted as ${\delta }_{1,\mathrm{ Poiss}}=P(n\le {N}_{\text{obs}}|{N}_{\text{fore}})$ and ${\delta }_{2,\mathrm{Poiss}}=P(n\ge {N}_{\text{obs}}|{N}_{\text{fore}})$, respectively. These two probabilities should be greater than a threshold (say 0.025 to reflect 95% confidence interval) to guarantee that *N*_obs_ will not lie within the tails of our forecast.

The proposed simulation-based workflow provides as output the posterior probability distribution for the forecasted number of events $N\left(M\ge {M}_{l}|{\varvec{\uptheta}},\mathbf{s}\mathbf{e}\mathbf{q}, {M}_{l}\right)$ according to Eq. (9) that is integrated over the aftershock zone **A**. Therefore, it is quite straightforward to compute the percentiles of this distribution (i.e., 50th, 16th, 84th, 2nd and 98th percentiles) to realize how well the forecasted number of earthquakes matches *N*_obs_. It is possible to estimate empirically the probabilities ${\delta }_{1,\mathrm{Bayes}}=P(n\le {N}_{\text{obs}}|{N}_{\text{fore}})$ and ${\delta }_{2,\mathrm{Bayes}}=P(n\ge {N}_{\text{obs}}|{N}_{\text{fore}})$ as the number of data that are $(\le {N}_{\text{obs}})$ and $(\ge {N}_{\text{obs}})$ divided by the number of samples simulated for ${\varvec{\uptheta}}$ (the number of samples $N\left(M\ge {M}_{l}|{\varvec{\uptheta}},\mathbf{s}\mathbf{e}\mathbf{q}, {M}_{l}\right)$ is equal to the number of generated samples of ${\varvec{\uptheta}}$).

### S-test based on the simulation-based Bayesian framework

The spatial distribution of forecast and observation can be compared based on the S-test^58,59^, which provides an overall measure of how well the forecast in single cell units (associated with the mesh grid of the space **A** of the aftershock zone centered at *x* and *y*) matches the observations in single cell units. According to this test, we calculate the log-likelihood of observations ${N}_{\mathrm{obs},n}$ given the forecasts ${N}_{\mathrm{fore},n}$ in each grid, where *n* = 1:*N*_*grid*_, assuming that the total number of cell units centered at the Cartesian coordinate (*x*, *y*)∈**A** is equal to *N*_*grid*_. Consider that ${N}_{\mathrm{fore}}={\sum }_{n=1}^{{N}_{grid}}{N}_{\mathrm{fore},n}$ and ${N}_{\mathrm{obs}}={\sum }_{n=1}^{{N}_{grid}}{N}_{\mathrm{obs},n}$, where ${N}_{\mathrm{fore},n}={\mathbb{E}}[N(x, y, {M}_{l}|{\varvec{\uptheta}},\mathbf{s}\mathbf{e}\mathbf{q}, {M}_{l})]$ (see Eq. 9) is the (expected) number of forecasted events with $M\ge {M}_{l}$ at the Cartesian coordinate (*x*, *y*)$\in$ **A**, and ${N}_{\mathrm{obs},n}$ is the number of observed events (with $M\ge {M}_{l}$) in the same cell unit within the forecasting interval [*T*_*start*_, *T*_*end*_]. We isolate the spatial forecasts so that sum of the number of events over the whole grid cells matches the observation; thus, we set ${N}_{\mathrm{fore},n}={N}_{\mathrm{fore},n}\cdot \frac{{N}_{\mathrm{obs}}}{{N}_{\mathrm{fore}}}$. We estimate the likelihood *S*_obs_ as:

33 $$S_{{{\text{obs}}}} = \ln \left( {\prod\limits_{n = 1}^{{N_{{{\text{grid}}}} }} {\frac{{\left( {N_{{{\text{fore}},n}} } \right)^{{N_{{{\text{obs}},n}} }} e^{{ - N_{{{\text{fore}},n}} }} }}{{N_{{{\text{obs}},n}} !}}} } \right){ = }\sum\limits_{n = 1}^{{N_{{{\text{grid}}}} }} {\left[ { - N_{{{\text{fore}},n}} + N_{{{\text{obs}},n}} \ln \left( {N_{{{\text{fore}},n}} } \right) - \ln \left( {N_{{{\text{obs}},n}} !} \right)} \right]}$$

The S-test is done herein based on the *standard* S-test^59^ and the *Bayesian* S-test. The two methods are described as follows:

1. The standard S-test accounts for the forecast uncertainty by simulating catalogues that are consistent with the forecast. For simulating each catalogue: (a) the number of events in each simulated catalog is normalized to match ${N}_{\mathrm{obs}}$; (b) For each event in the previous step, generate a uniform random number $\sim$ Uniform (0, 1) in order to locate the event in the corresponding spatial cell according to the proportion of ${N}_{\mathrm{fore},n}$ in the cell units (i.e., using the inverse cumulative distribution function). Stage (b) will lead to construction of ${N}_{\mathrm{sim},n}$ to be the number of simulated events (with ${M\ge M}_{l}$) in the cell unit (*x*, *y*)$\in$ **A**, where *n* = 1:*N*_*grid*_ considering that ${N}_{\mathrm{obs}}={\sum }_{n=1}^{{N}_{grid}}{N}_{\mathrm{sim},n}$. Repeating stage (b) many times (say e.g. 500 to 1000 iterations) leads to the construction of forecast-consistent simulated catalogues of events. Finally, for each simulated catalogue, the log-likelihood *S*_sim_ of ${N}_{\mathrm{sim},n}$ simulations given the forecasts ${N}_{\mathrm{fore},n}$, in all cell units can be calculated as follows:
  34 $$S_{{{\text{sim}}}} = \ln \left( {\prod\limits_{n = 1}^{{N_{{{\text{grid}}}} }} {\frac{{\left( {N_{{{\text{fore}},n}} } \right)^{{N_{{{\text{sim}},n}} }} e^{{ - N_{{{\text{fore}},n}} }} }}{{N_{{{\text{sim}},n}} !}}} } \right){ = }\sum\limits_{n = 1}^{{N_{{{\text{grid}}}} }} {\left[ { - N_{{{\text{fore}},n}} + N_{sim,n} \ln \left( {N_{{{\text{fore}},n}} } \right) - \ln \left( {N_{{{\text{sim}},n}} !} \right)} \right]}$$
  Repeating the calculation of Eq. (34) for each simulated catalog, we obtain the vector of log-likelihood ${\mathbf{S}}_{\mathrm{sim}}$.
2. The presented simulation-based workflow furnishes, as a side-product, the Bayesian S-test and forecast-consistent catalogues. Stage (b) in method (1) described above can be accomplished by directly using the normalized spatial seismicity forecasts $\left[N(x, y, {M\ge M}_{l}|{\varvec{\uptheta}},\mathbf{s}\mathbf{e}\mathbf{q}, {M}_{l})\cdot \frac{{N}_{\mathrm{obs}}}{{N}_{\mathrm{fore}}}\right]$ for the cell units based on different realizations of the vector of uncertain parameters ${\varvec{\uptheta}}$. Thus, the number of simulated catalogs is equal to the number of generated samples of ${\varvec{\uptheta}}$. The log-likelihood *S*_sim_ can be estimated according to Eq. (34).

This probability *P*(*S* ≤ *S*_obs_) can be estimated as follows:

35 $$P\left( {S \le S_{{{\text{obs}}}} } \right) = \frac{{{\mathbb{N}}\left( {{\mathbf{S}}_{{{\text{sim}}}} \le S_{{{\text{obs}}}} } \right)}}{{{\mathbb{N}}\left( {{\mathbf{S}}_{{{\text{sim}}}} } \right)}}$$

where ${\mathbb{N}}\left({\mathbf{S}}_{\mathrm{sim}}\le {S}_{\mathrm{obs}}\right)$ indicates the number of the components in the vector ${\mathbf{S}}_{\mathrm{sim}}$ that are ≤ *S*_obs_, and the denominator ${\mathbb{N}}\left({\mathbf{S}}_{\mathrm{sim}}\right)$ is the total number of elements in ${\mathbf{S}}_{\mathrm{sim}}$ which is equal to the number of simulated catalogs. If *S*_obs_ falls in the lower tail of the distribution *P*(*S* ≤ *S*_obs_), this indicates that the observation is not consistent with the forecast in each cell; thus, the forecast is not accurate.

## Supplementary Information

Supplementary Information.

## Data Availability

The earthquake catalog of Kermanshah seismic sequence used in this study and the MATLAB code developed for this workflow is available on a Git repository hosting service GitHub https://github.com/HossEbi/Bayesian_spatiotemporal_ETAS_model_ver2. The data is also available upon request from the corresponding author.
