# Supplementary material for: Improvements to seismicity forecasting based on a Bayesian spatio-temporal ETAS model
Source: Sci Rep. 2022 Dec 5;12:20970. doi: 10.1038/s41598-022-24080-1 (PMC9723186; doi:10.1038/s41598-022-24080-1)
Supplement: Supplementary file 1 — Supplementary Information. [file 41598_2022_24080_MOESM1_ESM.pdf]

Supplementary Information
Scientific Reports
https://doi.org/10.1038/s41598-022-24080-1

Improvements to seismicity forecasting based on a Bayesian spatio-temporal ETAS model

Hossein Ebrahimian[1*], Fatemeh Jalayer[2,3], Behnam Maleki Asayesh[4,5], Sebastian Hainzl[6], Hamid Zafarani[7]

[1] Department of Structures for Engineering and Architecture, University of Naples Federico II, Naples, Italy, e-mail: ebrahimian.hossein@unina.it, (corresponding Author)

[2] Institute for Risk and Disaster Reduction (IRDR), University College London, London, UK, email: f.jalayer@ucl.ac.uk

[3] Department of Structures for Engineering and Architecture, University of Naples Federico II, Naples, Italy, e-mail: fatemeh.jalayer@unina.it.

[4] Institute of Geosciences, University of Potsdam; GFZ German Research Center for Geosciences, Postdam, Germany, e-mail: maleki@gfz-potsdam.de

[5] GFZ German Research Center for Geosciences, Postdam, Germany, e-mail: maleki@gfz-potsdam.de

[6] GFZ German Research Center for Geosciences, Postdam, Germany, e-mail: hainzl@gfz-potsdam.de

[7] International Institute of Earthquake Engineering and Seismology (IIEES), Tehran, Iran, e-mail: h.zafarani@iiees.ac.ir

SI-Method

SI-1. Markov Chain Monte Carlo (MCMC) simulation procedure

SI-1.1. Metropolis-Hastings (MH) algorithm

In order to sample from the posterior distribution $p(\boldsymbol{\theta}|\mathbf{seq},M_l)$, Markov Chain Monte Carlo (MCMC) simulation routine is employed herein. The MCMC routine here employs the Metropolis-Hastings (MH) algorithm. The MH algorithm generates a Markov chain that produces a sequence of samples $[\boldsymbol{\theta}_1 \to \boldsymbol{\theta}_2 \to \cdots \to \boldsymbol{\theta}_i \to \cdots]$, where $\boldsymbol{\theta}_i$ represents the state of Markov chain at i^{th} iteration (the first few samples are often discarded to reduce the initial transient effect). A Markov chain is a stochastic process where the transition from current state to a new state is done by using a conditional transition function that is conditioned on the current (last) state. Given the Markovian nature of MCMC simulation scheme, and to generate $(i+1)^{\text{th}}$ sample $\boldsymbol{\theta}_{i+1}$ from the current i^{th} sample $\boldsymbol{\theta}_i$ based on MH routine, the following procedure is done:

(a) Simulate a *candidate* sample $\boldsymbol{\theta}^*$ from a *proposal* distribution $q(\boldsymbol{\theta}|\boldsymbol{\theta}_i)$. It is important to note that there are no specific restrictions about the choice of $q(\cdot)$ apart from the fact that it should be possible to calculate both $q(\boldsymbol{\theta}_{i+1}|\boldsymbol{\theta}_i)$ and $q(\boldsymbol{\theta}_i|\boldsymbol{\theta}_{i+1})$.

(b) Calculate the acceptance probability $\min(1,r)$, where r is defined as follows (see also Equation 19 of the manuscript):

$$r=\frac{p\left(\boldsymbol{\theta}^*\middle|\mathbf{seq},M_l\right)}{p\left(\boldsymbol{\theta}_i\middle|\mathbf{seq},M_l\right)}\cdot\frac{q\left(\boldsymbol{\theta}_i\middle|\boldsymbol{\theta}^*\right)}{q\left(\boldsymbol{\theta}^*\middle|\boldsymbol{\theta}_i\right)}=\left(\underbrace{\frac{p\left(\mathbf{seq}\middle|\boldsymbol{\theta}^*,M_l\right)}{p\left(\mathbf{seq}\middle|\boldsymbol{\theta}_i,M_l\right)}}_{\text{likelihood ratio}}\cdot\underbrace{\frac{p\left(\boldsymbol{\theta}^*\middle|M_l\right)}{p\left(\boldsymbol{\theta}_i\middle|M_l\right)}}_{\text{prior ratio}}\right)\cdot\underbrace{\frac{q\left(\boldsymbol{\theta}_i\middle|\boldsymbol{\theta}^*\right)}{q\left(\boldsymbol{\theta}^*\middle|\boldsymbol{\theta}_i\right)}}_{\text{proposal ratio}} \tag{SI-1}$$

(c) Generate u from a Uniform distribution between (0, 1), i.e., $u \sim$ Uniform (0, 1);
- if $u \le \min(1,r) \to$ set $\boldsymbol{\theta}_{n+1}=\boldsymbol{\theta}^*$ (*accept* the *candidate* state to be taken as the *next* state of the Markov chain);
- else set $\boldsymbol{\theta}_{n+1}=\boldsymbol{\theta}_n$ (the *current* state is taken as the *next* state)

SI-1.2. Applying an adaptive Metropolis-Hastings algorithm (adaptive MCMC)

In order to improve the rate of convergence of the simulation process, we used an adaptive MH algorithm (as proposed in[52]) that introduces a sequence of intermediate candidate evolutionary PDF's that resemble more and more the target PDF. Let $\{p_1, p_2,\ldots, p_{Nchain}\}$ be the sequence (*chain*) of PDF's leading to $p(\boldsymbol{\theta}|\mathbf{seq},M_l)=p_{Nchain}$, where *Nchain* is the number of chains and each chain contains *Nseed* samples (as indicated subsequently). The following adaptive simulation-based procedure is employed:

- Simulate *Nseed* samples $\{\boldsymbol{\theta}_1, \boldsymbol{\theta}_2, \ldots, \boldsymbol{\theta}_{Nseed}\}^{(1)}$, where the superscript (1) denotes the first simulation level (first chain), with the target PDF p_1 as the first sequence of samples (i.e., first simulation level, or the first chain). Since we have no idea of choosing a proposal PDF to get MCMC samples, and instead of accepting or rejecting a proposal for $\boldsymbol{\theta}$ involving all its components simultaneously (called *block-wise* updating scheme), it might be computationally simpler and more efficient at the first stage to make proposals for individual components of $\boldsymbol{\theta}$, one at a time (a *component-wise* updating approach). In the so-called block-wise updating, the proposal distribution has the same dimension as the target distribution. For instance, if the vector of model parameters $\boldsymbol{\theta}$ involve n uncertain parameters (herein, n can vary from 6 to 8 parameters), we design an n-dimensional proposal distribution, and either accept or reject the candidate state (with all n variables) as a block. The block-wise updating approach can be associated with high rejection rates. This may cause problem when we want to generate the first sequence of samples. Therefore, we utilize the more stable component-wise updating for the first chain. Knowing that the vector of ETAS model parameters $\boldsymbol{\theta}$ has n=6 to 8 variables (depending on whether we calculate K separately or

leave it to MCMC updating, respectively and also on the type of the kernel density of distance), we start from the first variable and generate a candidate state based on a proposal distribution for this individual component, and finally accept or reject it based on MH algorithm. Note that in this stage, we have only varied the current component and kept the other variables in vector $\boldsymbol{\theta}$ constant. Then, we move to the next components one-by-one and do the same procedure, while considering the previous (updated) components. Therefore, what happens in the current step is conditional on the updated parameters in the previous steps. The proposal distribution for each component is assumed to be a lognormal distribution herein. The prior PDF for each component is a lognormal distribution (as shown in Equation 20 of the manuscript). If the parameter K is being calculated according to *method* (*a*) in Section 4.2.2 of the manuscript, this should be done after each realization of other uncertain parameters.

- Construct a kernel density function $\kappa^{(1)}$ as the weighted sum (average) of n-dimensional Gaussian PDFs centered among the samples $\{\boldsymbol{\theta}_1, \boldsymbol{\theta}_2, \ldots, \boldsymbol{\theta}_{Nseed}\}^{(1)}$, with the covariance matrix $\mathbf{S}^{(1)}$ of the samples $\boldsymbol{\theta}_i^{(1)}$, where i=1:*Nseed*, with the weights associated to each sample as w_i, as follows (see Ang et al. 1992, Au and Beck 2002):

$$\kappa^{(1)}(\boldsymbol{\theta}) = \frac{1}{Nseed}\sum_{i=1}^{Nseed}\frac{1}{w_i^{\,n}\sqrt{(2\pi)^n\left|\mathbf{S}^{(1)}\right|}}\exp\left(-\frac{1}{2w_i^{\,2}}\left(\boldsymbol{\theta}-\boldsymbol{\theta}_i^{(1)}\right)^{\mathrm{T}}\left(\mathbf{S}^{(1)}\right)^{-1}\left(\boldsymbol{\theta}-\boldsymbol{\theta}_i^{(1)}\right)\right) \tag{SI-2}$$

The kernel density $\kappa^{(1)}$ constructed in Equation (SI-2) approximates p_1. The kernel function κ can be viewed as a PDF consisting of bumps at $\boldsymbol{\theta}_i$, where width w_i controls the common size of the bumps. Therefore, a large value of w_i tends to over-smooth the kernel density, while a small value may cause noise-shaped bumps. In view of this, the w_i can be assumed to have a fixed width (= w), or alternatively the *adaptive kernel* estimate can be employed (Ang et al. 1992, Au and Beck 1999) that is defined for each sample $\boldsymbol{\theta}_i$, i=1:*Nseed*. The adaptive kernel has better convergence and smoothing properties over the fixed-width kernel estimate. The fixed width w is estimated as follows (Au and Beck 2002):

$$w=\left(\frac{4}{(n+2)N_d}\right)^{\frac{1}{n+4}} \tag{SI-3}$$

where N_d is the number of distinct samples ($N_d \le Nseed$). For one-dimensional problems, this leads to the well-known fixed-width value of $[(4/3)/Nseed]^{1/5}$ (see Silverman 1986). The reason for using N_d is due to the fact that for the next simulation levels, where we are going to use a block-wise updating approach in the MCMC scheme, one may be faced with rejection of candidate states within the Markov chain. Thus, we need to count the distinct samples.

In the adaptive kernel method, the idea is to use a larger width in regions of lower probability density. Following the general strategy used in the past (see Ang et al. 1992, Au and Beck 1999), the adaptive band width w_i for the ith sample $\boldsymbol{\theta}_i$ can be written as $w_i = w\lambda_i$, where the local bandwidth factor λ_i can be estimated as follows:

$$\lambda_i=\left(\kappa(\boldsymbol{\theta}_i)\Big/\left(\prod_{j=1}^{Nseed}\kappa(\boldsymbol{\theta}_j)\right)^{\frac{1}{Nseed}}\right)^{-\omega} \tag{SI-4}$$

where $0\le \omega \le 1.0$ is the sensitivity factor, and $\kappa(\boldsymbol{\theta}_i)$ is calculated based on Equation (SI-2) where $\boldsymbol{\theta}=\boldsymbol{\theta}_i$ with the choice of fixed-width w (see Equation SI-3). The denominator in Equation (SI-4) is a geometric mean of the kernel estimator at all *Nseed* points. It is numerically more stable to estimate first $[\kappa(\boldsymbol{\theta}_j)]^{1/Nseed}$ and then multiply it for all j=1: *Nseed* values (i.e., estimate denominator as $\prod_{j=1}^{Nseed}\left[\kappa(\boldsymbol{\theta}_j)^{1/Nseed}\right]$). Abramson (1982) showed that varying the bandwidth proportional to $\kappa^{-1/2}$ (i.e., ω=0.50) provides good performance for the kernel smoothing density. To this end, the value of ω=0.50 is employed here in this work as also suggested by other research endeavors (Ang et al. 1992, Au and Beck 1999).

- Simulate *Nseed* Markov chain samples $\{\theta_1, \theta_2, \ldots, \theta_{Nseed}\}^{(2)}$ with the target PDF p_2 as the second simulation level (chain). We use $\kappa^{(1)}$ as the proposal distribution $q(\cdot)$ in Equation (SI-1) in this stage to generate the second chain of samples. To generally simulate sample θ from the kernel $\kappa^{(nc)}$ (where nc=1:*Nchain*), we generate a discrete random index from the vector [1, 2, …, *Nseed*] with the corresponding weights $[w_1, w_2, \cdots, w_{Nseed}]$ using an inverse transformation sampling; if index=j, then generate θ from the Gaussian PDF κ_j, where:

$$\begin{aligned}\kappa_j(\boldsymbol{\theta}) &= \frac{1}{(w\lambda_j)^n\sqrt{(2\pi)^n\left|\mathbf{S}^{(nc)}\right|}}\cdot\exp\left(-\frac{1}{2(w\lambda_j)^2}(\boldsymbol{\theta}-\boldsymbol{\theta}_j)^{\mathrm{T}}\left(\mathbf{S}^{(nc)}\right)^{-1}(\boldsymbol{\theta}-\boldsymbol{\theta}_j)\right)\\ &= \frac{1}{\sqrt{(2\pi)^n\left|\mathbf{S}_j^{(nc)}\right|}}\cdot\exp\left(-\frac{1}{2}(\boldsymbol{\theta}-\boldsymbol{\theta}_j)^{\mathrm{T}}\left(\mathbf{S}_j^{(nc)}\right)^{-1}(\boldsymbol{\theta}-\boldsymbol{\theta}_j)\right)\end{aligned} \quad \text{(SI-5)}$$

  where $\mathbf{S}_j^{(nc)} = w_j{}^2\mathbf{S}^{(nc)}$, where $\mathbf{S}^{(nc)}$ is the covariance matrix of the samples $\{\theta_1, \theta_2, \ldots, \theta_{Nseed}\}^{(nc)}$. The next Section SI-2 shows how a sample θ can be drawn from the Gaussian PDF κ_j. From this sequence on, the MCMC updating is done in a block-wise manner as we generate a candidate θ and accept/reject it as a block. The second chain of samples $\{\theta_1, \theta_2, \ldots, \theta_{Nseed}\}^{(nc=2)}$ are then used to construct the kernel density $\kappa^{(2)}$ based on Equation (SI-2).

- In general, $\kappa^{(nc)}$ is used as the proposal distribution in order to move from the nc^{th} simulation level (which approximates p_{nc}) into $(nc+1)^{\text{th}}$ chain (with target PDF p_{nc+1}). This will continue until the $Nchain^{\text{th}}$ simulation level where Markov chain samples are simulated for the target updated $p(\theta|\mathbf{seq},M_l)=p_{Nchain}$.

References of Section SI-1:

Ang, G. L., Ang, A. H. S., & Tang, W. H. Optimal importance-sampling density estimator. *J Eng. Mech.* **118**, 6, 1146-1163 (1992).

Au, S. K., & Beck, J. L. A new adaptive importance sampling scheme. *Struct. Safety* **21**, 135–158 (1999).

SI-2. Generating dependent Gaussian variables

A positive definite matrix $\mathbf{S}_{n\times n}$ can always be factorized base on Cholesky decomposition as follows:

$$\mathbf{S} = \mathbf{L}\mathbf{L}^T \tag{SI-6}$$

where $\mathbf{L}_{n\times n}$ is a lower triangular matrix (i.e., for all $j>i$, $L_{ij}=0$ where L_{ij} denotes the (i, j)-entry of the matrix $\mathbf{L}$). A Gaussian vector $\boldsymbol{\theta}_{n\times 1}$ with mean $\boldsymbol{\mu}_{\boldsymbol{\theta}}$ and covariance $\mathbf{S}$ can be generated as follows:

$$\boldsymbol{\theta} = \boldsymbol{\mu}_{\boldsymbol{\theta}} + \mathbf{L}\mathbf{Z} \tag{SI-7}$$

where $\mathbf{Z}_{n\times 1}$ is a vector of standard Gaussian *i.i.d.* random variables with zero mean $\mathbf{0}_{n\times n}$, and covariance equal to the identity matrix $\mathbf{I}_{n\times n}$. In order to see why the vector $\boldsymbol{\theta}$ can be written according to Equation (SI-10), we verify the properties of $\boldsymbol{\theta}$ as follows. The expectation of $\boldsymbol{\theta}$, denoted as $\mathbb{E}(\boldsymbol{\theta})$, can be defined as:

$$\mathbb{E}(\boldsymbol{\theta}) = \mathbb{E}(\boldsymbol{\mu}_{\boldsymbol{\theta}} + \mathbf{L}\mathbf{Z}) = \mathbb{E}(\boldsymbol{\mu}_{\boldsymbol{\theta}}) + \mathbf{L}\underbrace{\mathbb{E}(\mathbf{Z})}_{=\mathbf{0}_{n\times 1}} = \boldsymbol{\mu}_{\boldsymbol{\theta}} \tag{SI-8}$$

The covariance matrix of $\boldsymbol{\theta}$ can be written as:

$$\mathbb{E}\left[(\boldsymbol{\theta}-\boldsymbol{\mu}_{\boldsymbol{\theta}})(\boldsymbol{\theta}-\boldsymbol{\mu}_{\boldsymbol{\theta}})^T\right] = \mathbb{E}\left(\mathbf{L}\mathbf{Z}\mathbf{Z}^T\mathbf{L}^T\right) = \mathbf{L}\underbrace{\mathbb{E}\left(\mathbf{Z}\mathbf{Z}^T\right)}_{=\mathbf{I}_{n\times n}}\mathbf{L}^T = \mathbf{L}\mathbf{L}^T = \mathbf{S} \tag{SI-9}$$

Thus, $\boldsymbol{\theta}$ expressed as Equation (SI-7), has a mean equal to $\boldsymbol{\mu}_{\boldsymbol{\theta}}$ and covariance matrix equal to $\mathbf{S}$.

SI-Results

SI-3. Estimating/validating the completeness magnitude M_c for different forecasting intervals in phase 1 of the seismic sequence

Discussion on the estimation of the completeness magnitude M_c

The completeness magnitude M_c is theoretically defined as the lowest magnitude at which 100% of the earthquakes in a space-time volume are detected (Rydelek and Sacks 1989). Following a large earthquake, there exists a detection problem of small aftershocks that cannot be distinguished from the coda wave of the mainshock (i.e. increased noise). So, while our network is capable of detecting small events in normal situation, it could not register those events in the immediate aftermath of a large earthquake. Therefore, first minutes and hours after a large mainshock, small aftershocks are missed, and therefore, we need to increase M_c due to the detection problem.
To address the catalogue incompleteness, the completeness magnitude M_c at the beginning of each forecasting interval is checked so that the desired lower magnitude M_l be always greater than or equal to M_c, i.e., $M_l \geq M_c$. The issue of magnitude incompleteness seems to be more critical when providing early forecasts in the immediate aftermath of a main seismic event. This can be attributed both to the lack of data in the short time elapsed after the main event and the missing data in certain magnitude ranges. Nevertheless, as more time passes and the observation history at the time of forecast starts to become more populated, the magnitude incompleteness seems to be less critical. Herein, we have employed three alternative methods to check/estimate the completeness magnitude that varies through time:

1. Frequency-magnitude distribution plot of the aftershock events in the observation history seq available at the time of issuing the forecast, i.e., [T_o, T_{start}): the normal trend in frequency-magnitude curve has an approximately exponential decrease of N that stands for the number of aftershock with magnitudes equal to or greater than m, $N(M \geq m)$, as the observed magnitude m increases (i.e., linearly decreasing in logarithmic scale following a Gutenberg-Richter (GR) relationship. In case that the data is incomplete, a flattening in a certain lower magnitude range (having higher frequencies) can be monitored. Accordingly, M_c is visually identified as the point where the magnitude-frequency curve becomes approximately linear in the semi-logarithmic scale (see e.g., Figure 3a in the section *Results* and also similar figures in this section).

2. Bayesian updating approach for calculating β-value versus various magnitude thresholds: M_c is detected as the magnitude threshold, denoted here as m_l, where the maximum likelihood of the posterior probability distribution of β (mode of the distribution denoted as β_{ML} herein) rendered by the Bayesian updating approach becomes quasi invariant or reaches its peak value with respect to the adopted m_l. This magnitude threshold can be interpreted as M_c (see Ebrahimian et al. 2014; Ebrahimian and Jalayer 2017; Erahimian et al. 2019). The posterior probability distribution for β given the data **D** and a magnitude threshold m_l is denoted herein as $p(\beta|\mathbf{D}, m_l)$. Data **D** consists of all the events in the catalog in the time span of [T_o, T_{start}) with $M \geq m_l$. The probability $p(\beta|\mathbf{D}, m_l)$ can be determined according to the Bayes's theorem as (see also Ebrahimian et al. 2014, Ebrahimian and Jalayer 2017):

$$p\left(\beta|\mathbf{D},m_l\right)=c^{-1}p\left(\mathbf{D}|\beta,m_l\right)p\left(\beta|m_l\right)=c^{-1}\left(\prod_k p\left(m_k|\beta,m_k\geq m_l\right)\right)p\left(\beta\right)=c^{-1}\left(\prod_k \beta e^{-\beta\left(m_k-m_l\right)}\right)p\left(\beta\right) \quad \text{(SI-13)}$$

where c^{-1} is the normalizing constant of the Bayes's expression; $p(\beta|\mathbf{D}, m_l)$ is the likelihood function for data **D** given β and m_l; $p(\beta|m_l) \cong p(\beta)$ is the prior probability distribution. The likelihood $p(\mathbf{D}|\beta, m_l)$ can be seen as the multiplication of $p(m_k|\beta, m_k \geq m_l)$, which is the conditional probability of observing events with magnitude $m_k \geq m_l$. Herein, we use a Uniform probability distribution to define the prior $p(\beta)$ indicating non-in formative prior. For instance, Figure 3b (section *Results*; see also similar figures in this section) illustrates β_{ML}-value calculated as the maximum likelihood (mode) of posterior probability distribution $p(\beta|\mathbf{D}, m_l)$, with respect to various magnitude thresholds m_l. It can be seen that β_{ML} increases monotonically with respect to m_l up to a certain value that indicates M_c.

3. The semi-logarithmic plots showing the observed earthquake magnitudes as a function of the time elapsed after the mainshock is a simple way to detect the missed aftershocks with small magnitudes directly after the mainshock. This is a proper visual check to ensure that observed catalog of data is complete for magnitudes greater than M_c that is selected by either of previous two approaches. Therefore, the data in seq (see section 4.2) that will be used for updating the ETAS model parameters does not include small magnitude ranges missed in an early time interval right after the occurrence of the main event. Figure 3c (Section *Results*; see also similar figures in this section) provides a graphical control to see how well M_c is estimated by the previous two methods so that at the time of starting the forecast, the seq is complete in the range of magnitudes above M_c. The semi-logarithmic plot indicates the presence of voids in the magnitude range right after the occurrence of main event that can be kept away by the selected level of M_c.

Plots for M_c

The graphical representation for finding M_c based on three methods described in Section 4.2.1 of the manuscript for four forecasting intervals right after the occurrence of the Azgeleh event with Mw7.3 is shown in Figure SI-1 to Figure SI-4.

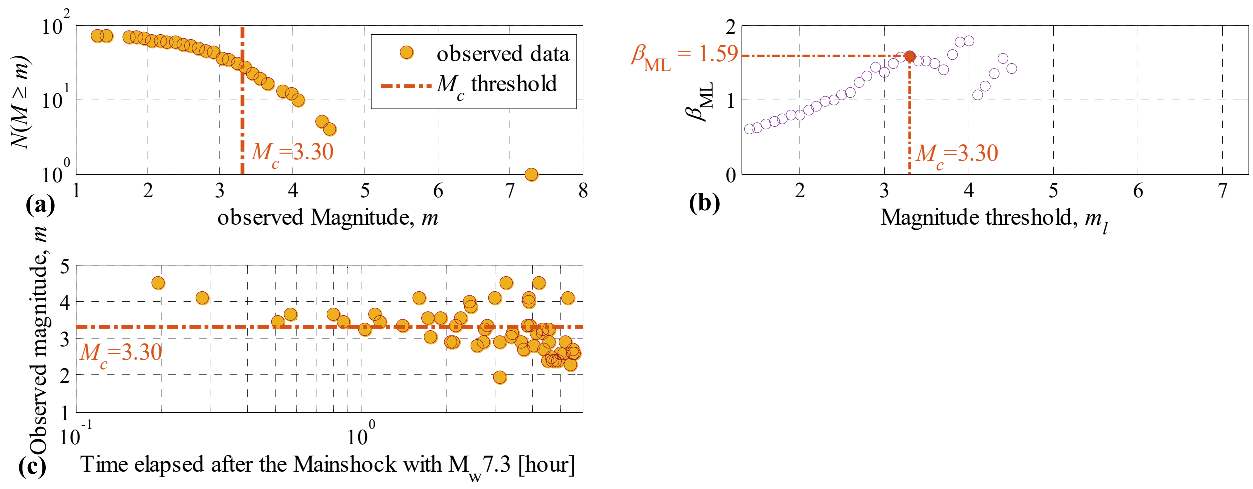

Figure SI-1: Visual presentation of three strategies introduced in Section 4.2.1 (*Methods*) for estimation/validation of M_c for the first forecasting interval [13/11/2017-00:00UTC, 13/11/2017-06:00UTC] in phase 1 of the seismic sequence

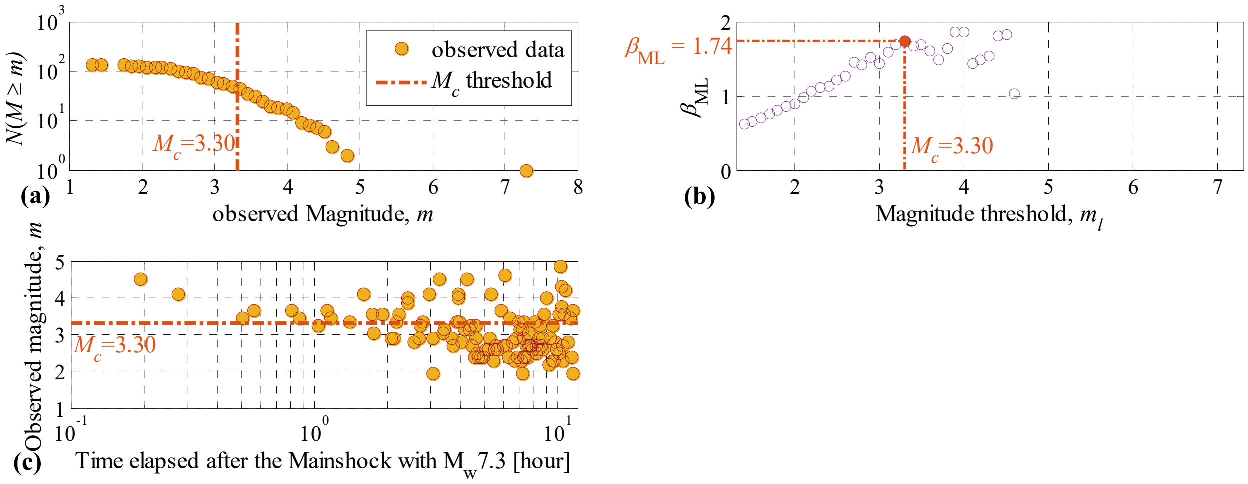

Figure SI-2: Visual presentation of three strategies introduced in Section 4.2.1 (*Methods*) for estimation/validation of M_c for the first forecasting interval [13/11/2017-06:00UTC, 14/11/2017-06:00UTC] in phase 1 of the seismic sequence

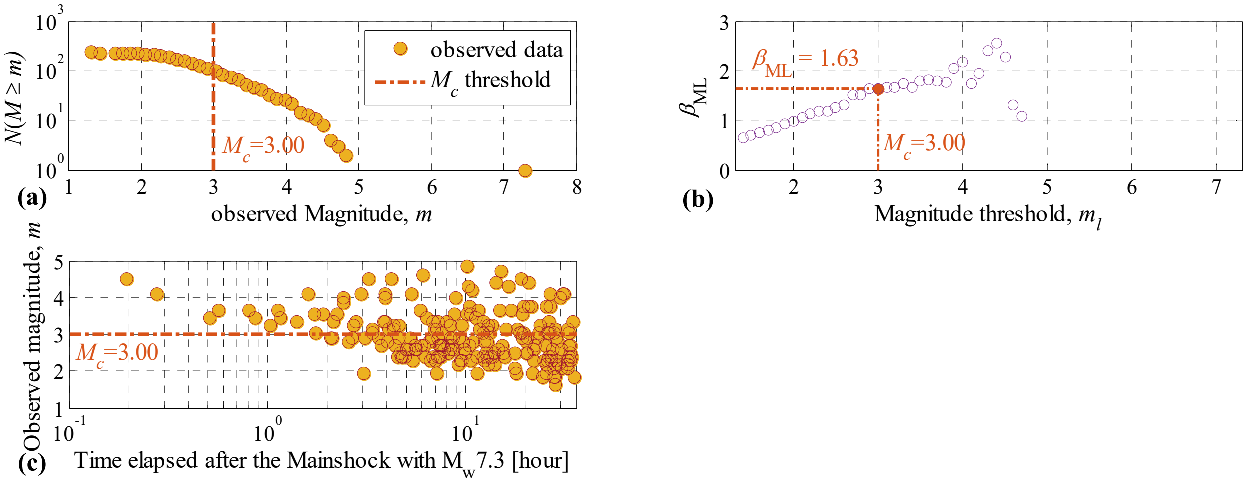

Figure SI-3: Visual presentation of three strategies introduced in Section 4.2.1 (*Methods*) for estimation/validation of M_c for the first forecasting interval [14/11/2017-06:00UTC, 15/11/2017-06:00UTC] in phase 1 of the seismic sequence

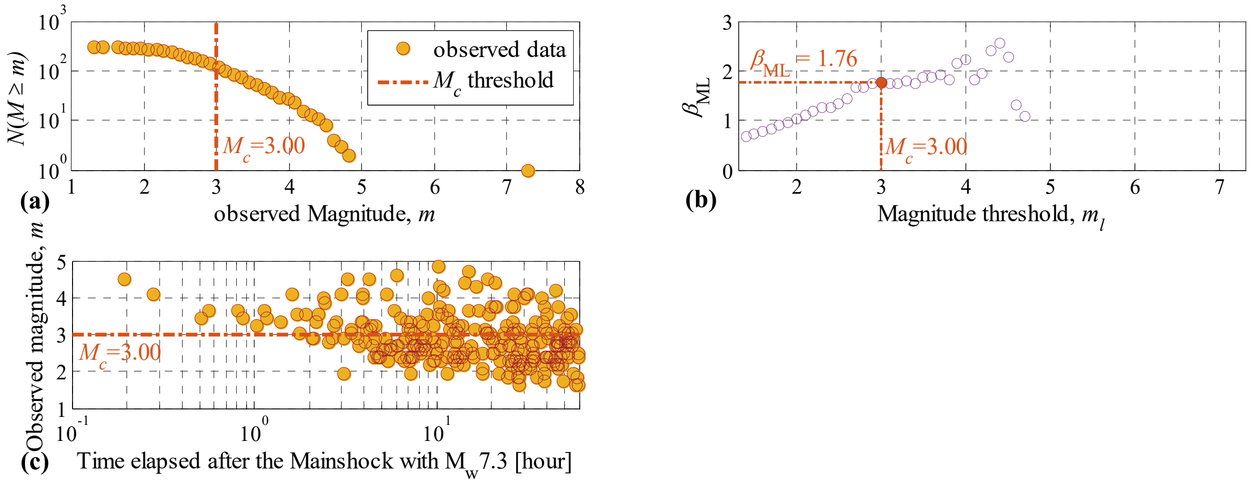

Figure SI-4: Visual presentation of three strategies introduced in Section 4.2.1 (*Methods*) for estimation/validation of M_c for the first forecasting interval [15/11/2017-06:00UTC, 16/11/2017-06:00UTC] in phase 1 of the seismic sequence

References of Section SI-3:

Rydelek, P. A., & Sacks I. S. Testing the completeness of earthquake catalogs and the hypothesis of self-similarity. *Nature* **337**, 251–253 (1989).

SI-4. Background Seismicity and the maximum expected magnitude $M_{\max}$

In this section, we discuss the methods used in order to assess maximum expected earthquake magnitude $M_{\max}$ (see Section 4.4, *Methods*, of the original manuscript) and background seismicity rate (denoted as $\mu(x, y|m_l)$ in Section 4.2 , *Methods*, of the original manuscript) for various magnitude lower magnitude thresholds m_l. To this end, two catalogs are combined including:

(1) A comprehensive and uniform earthquake catalogue for the Iranian Plateau (Mousavi-Bafrouei and Babaie Mahani 2020) for the time period before 2006: this catalog contains data in the period of 400 B.C. to the end of 2018 considering that all the magnitude scales are converted to moment magnitude M_w.

(2) The Iranian Seismological Center (IRSC) catalog based on IGUT seismic network (Institute of Geophysics, University of Tehran) for the period from 2006 up to end of October 2017.

We have used seismic events that occurred in the area with longitude ranging between [44°, 48°] and latitude ranging between [32°, 37°], which is slightly larger than the one shown in Figure 1 of the original manuscript, and in the time domain before 01/11/2017 (which is the starting time T_o for the seismic sequence in Phase 1, see Figure 2 of the original manuscript). Figure SI-5(a) shows the tectonic features and seismogenic sources of the considered zone for calculating the background seismicity together with the location of the events scaled by their magnitudes from the above two catalogs. The declustered catalog of Mousavi-Bafrouei and Babaie Mahani (2020) for the study area contains 493 events in the time interval [01/01/859 – 31/12/2005] with magnitude ranging from 3.5 to 7.0. The standard error in magnitude determination is less than 0.3 for this catalog. We obtain magnitude of completeness $M_c =$ 4.5 for this catalog by using Entire Magnitude Range (EMR) method (Ogata and Katsura, 1993; Woessner and Wiemer, 2005), as shown in Figure SI-5(b). After elimination of events with magnitude less than M_c, only 268 events remained in this catalog. For the temporal period of [01/01/2006 – 31/10/2017], we used IRSC catalog containing 9023 events with the magnitude range [0.14-6.32] before declustering. The standard error in magnitude determination was less than 0.2 for this catalog. This catalog is also declustered by using conjugate windows which is based on Gardner and Knopoff (1974) space-time declustring method. We found 763 clusters of earthquakes with a total number of 4811 (about 53.32%) events out of 9023. By using the EMR approach, we obtained magnitude of completeness $M_c = 1.5$, as shown in Figure SI-5(c). After elimination of events with magnitude less than M_c, about 3174 events remained in this catalog. To estimate the region-characteristic maximum possible earthquake magnitude ($M_{\max}$), we have employed the methodology proposed by Kijko and Singh (2011). Kijko and Singh (2011) present several probabilistic (statistical) techniques for the evaluation of $M_{\max}$, which can be used depending on the assumptions about the statistical distribution model and/or the information available about past seismicity. By using AUE package (Kijko and Sellevoll 1989; Kijko and Singh 2011), we estimated $M_{\max}$=7.5 ±0.25, as well as the occurrence rate of different magnitude thresholds for the study area. This result is in agreement of Zafarani et al. (2020) study that they estimated maximum possible earthquake for the seismogenic zones in this area about 7.5. Table SI-1 illustrates the annual rate of exceedance $\lambda(M \geq m_l)$, the return period ($T_R = 1/\,\lambda(M \geq m_l)$, years), and the probability of exceedance in different periods of time t ($P_t = 1 - \exp[-\,\lambda(M \geq m_l) \cdot t]$, where t is in year) for different magnitude thresholds m_l. To this end, the daily spatial rate representing the background seismicity of the aftershock zone **A** (with the area equal to A) with $M \geq m_l$ in the cell unit centered at $(x, y)\in\mathbf{A}$ can be estimated simply as $\mu(x, y|m_l) = \frac{\lambda(M \geq m_l)}{365 \cdot A}$. It is to note that in this study, our forecasting time unit is [day].

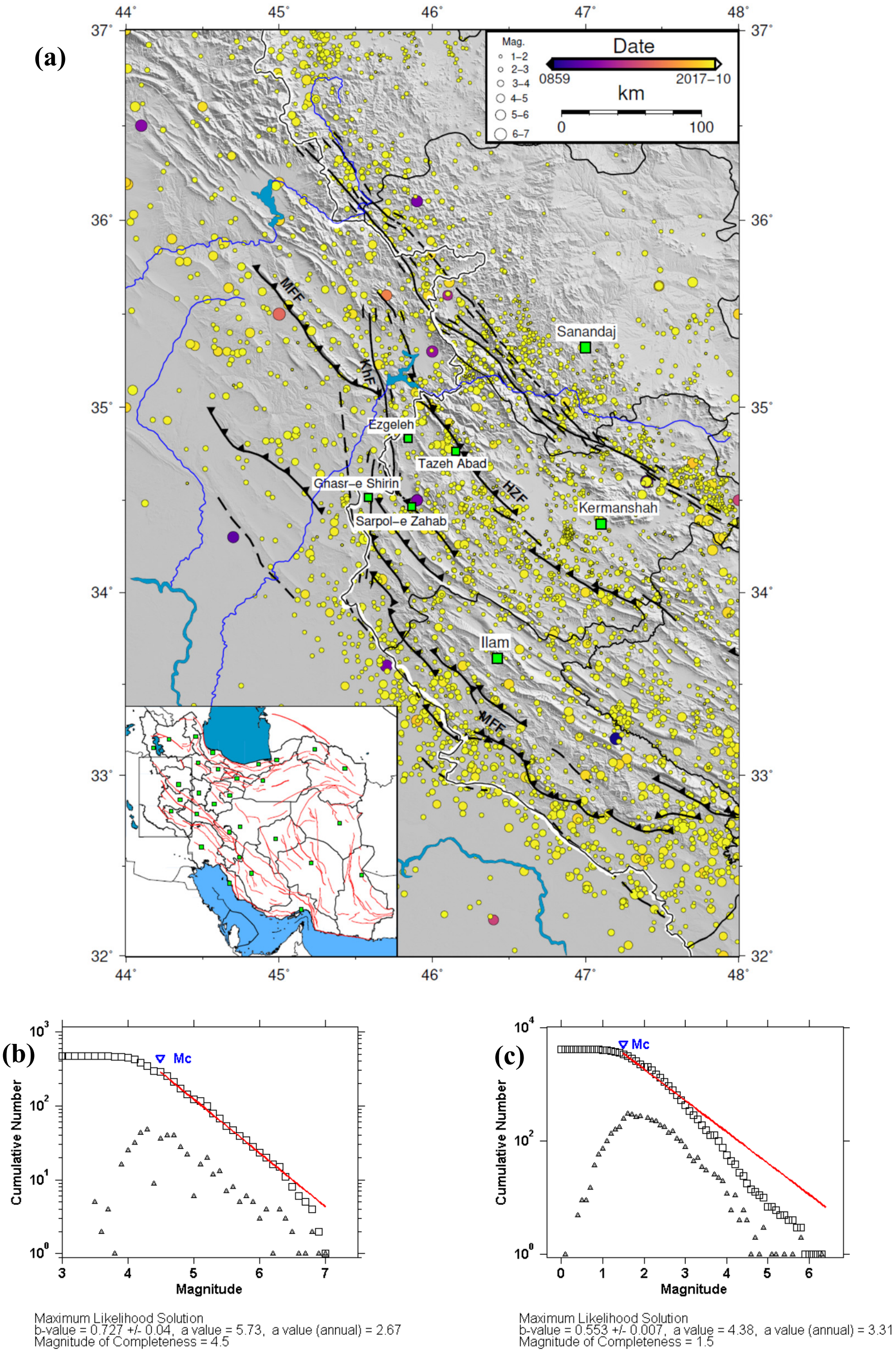

Figure SI-5: (a) Tectonic features and epicenter of the seismic events scaled by their magnitudes based on the two catalogs; Magnitude of completeness for (b) catalog from 859 to end of 2005 (Mousavi-Bafrouei and Babaie Mahani, 2020), (c) IRSC catalog from 2006 up to October 2017.

Table SI-1: The annual rate of exceedance, the return period (years), and the probability of exceedance in different periods of time based on various magnitude thresholds M_l.

Magnitude threshold m_l.	$\lambda(M \geq M_l)$	T_R	P_1	P_{50}	P_{100}	P_{1000}
1.5	9.4157	0.106	0.99939	1	1	1
1.6	8.3349	0.12	0.99878	1	1	1
1.7	7.3846	0.135	0.99769	1	1	1
1.8	6.5484	0.153	0.99587	1	1	1
1.9	5.8117	0.172	0.99297	1	1	1
2	5.1622	0.194	0.9886	1	1	1
2.1	4.5889	0.218	0.98231	1	1	1
2.2	4.0826	0.245	0.97365	1	1	1
2.3	3.6349	0.275	0.9622	1	1	1
2.4	3.2387	0.309	0.94763	1	1	1
2.5	2.8878	0.346	0.92969	1	1	1
2.6	2.5768	0.388	0.90829	1	1	1
2.7	2.3008	0.435	0.88348	1	1	1
2.8	2.0558	0.486	0.85543	1	1	1
2.9	1.838	0.544	0.82446	1	1	1
3	1.6443	0.608	0.79095	1	1	1
3.1	1.4719	0.679	0.75539	1	1	1
3.2	1.3183	0.759	0.71828	1	1	1
3.3	1.1814	0.846	0.68013	1	1	1
3.4	1.0593	0.944	0.64144	1	1	1
3.5	0.95017	1.05	0.60269	1	1	1
3.6	0.85271	1.17	0.56428	1	1	1
3.7	0.76555	1.31	0.52659	1	1	1
3.8	0.68756	1.45	0.48992	1	1	1
3.9	0.61771	1.62	0.45452	1	1	1
4	0.55512	1.8	0.42057	1	1	1
4.1	0.49899	2	0.38821	1	1	1
4.2	0.44861	2.23	0.35753	1	1	1
4.3	0.40338	2.48	0.32859	1	1	1
4.4	0.36273	2.76	0.3014	0.99999	1	1
4.5	0.32617	3.07	0.27595	0.99999	1	1
4.6	0.29328	3.41	0.2522	0.99997	1	1
4.7	0.26366	3.79	0.23011	0.99993	1	1
4.8	0.23698	4.22	0.20962	0.99986	1	1
4.9	0.21292	4.7	0.19064	0.99971	1	1
5	0.19122	5.23	0.17311	0.99944	1	1
5.1	0.17162	5.83	0.15693	0.99896	0.99999	1
5.2	0.15393	6.5	0.14204	0.99813	0.99998	1
5.3	0.13794	7.25	0.12833	0.99677	0.99995	1
5.4	0.12347	8.1	0.11574	0.9946	0.99989	1
5.5	0.11039	9.06	0.10417	0.99128	0.99977	1
5.6	0.098536	10.1	0.09356	0.98636	0.99954	1
5.7	0.087802	11.4	0.08384	0.97934	0.99909	1
5.8	0.078072	12.8	0.07493	0.96959	0.99827	1
5.9	0.069247	14.4	0.06676	0.95647	0.99683	1
6	0.061239	16.3	0.05929	0.93929	0.9944	1
6.1	0.053968	18.5	0.05245	0.91739	0.99044	1
6.2	0.047363	21.1	0.04619	0.89013	0.98422	1
6.3	0.041359	24.2	0.04046	0.85699	0.97474	1

6.4	0.0359	27.9	0.03522	0.81754	0.96079	1
6.5	0.030932	32.3	0.03043	0.77152	0.94086	1
6.6	0.026409	37.9	0.02604	0.71882	0.91321	1
6.7	0.022289	44.9	0.02203	0.65951	0.87591	1
6.8	0.018535	54	0.01835	0.5938	0.82688	1
6.9	0.015112	66.2	0.01499	0.52208	0.76403	0.99998
7	0.011989	83.4	0.01191	0.44485	0.68531	0.99987
7.1	0.00914	109	0.0091	0.36274	0.58886	0.99928
7.2	0.006537	153	0.00651	0.27644	0.47309	0.99584
7.3	0.00416	240	0.00415	0.1867	0.3368	0.97522
7.4	0.001987	503	0.00198	0.0943	0.1792	0.84633

References of Section SI-4:

Gardner, J. K., Knopoff, L. Is the sequence of earthquakes in Southern California, with aftershocks removed, Poissonian?. *Bull. Seism. Soc. Am.*, **64**, 5, 1363-1367 (1974).

Kijko, A., Sellevoll, M. A.. Estimation of earthquake hazard parameters from incomplete data files. Part I. Utilization of extreme and complete catalogs with different threshold magnitudes. *Bull. Seism. Soc. Am.*, **79**, 3, 645-654 (1989).

Kijko, A., Singh, M. Statistical tools for maximum possible earthquake magnitude estimation. *Acta Geophysica*, **59**, 4, 674-700 (2011)

Mousavi-Bafrouei, S.H., Mahani, A.B. A comprehensive earthquake catalogue for the Iranian Plateau (400 B.C. to December 31, 2018). *J Seismol.*, **24**, 709–724 (2020).

Ogata, Y., Katsura, K. Analysis of temporal and spatial heterogeneity of magnitude frequency distribution inferred from earthquake catalogues. *Geophys. J. Int.*, **113**, 3, 727-738 (1993).

Woessner, J., Wiemer, S. (2005). Assessing the quality of earthquake catalogues: Estimating the magnitude of completeness and its uncertainty. *Bull. Seism. Soc. Am.*, **95**, 2, 684-698 (2005).

Zafarani, H., Jafarian, Y., Eskandarinejad, A., Lashgari, A., Soghrat, M. R., Sharafi, H., & Haji-Saraei, M. A. E. (2020). Seismic hazard analysis and local site effect of the 2017 Mw 7.3 Sarpol-e Zahab, Iran, earthquake. *Nat. Hazards*, **103**, 1783–1805 (2020).

SI-5. Correlation between the ETAS model parameters in Phase 1 shown in Table 1 of the original manuscript

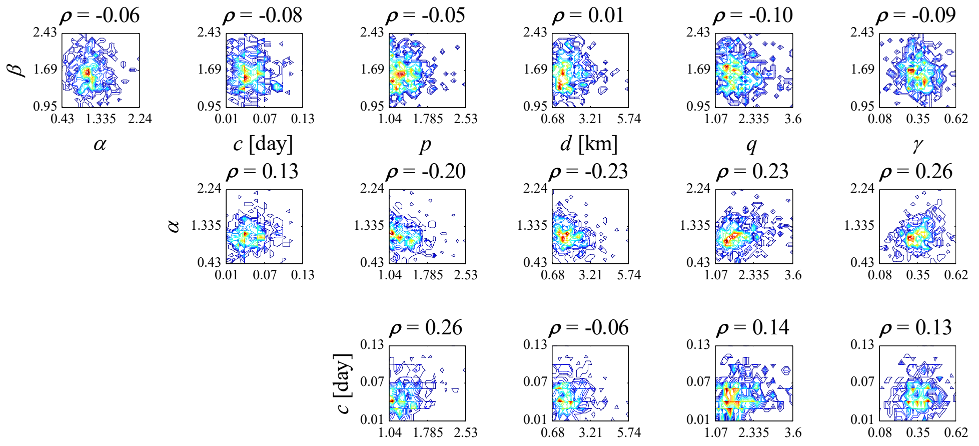

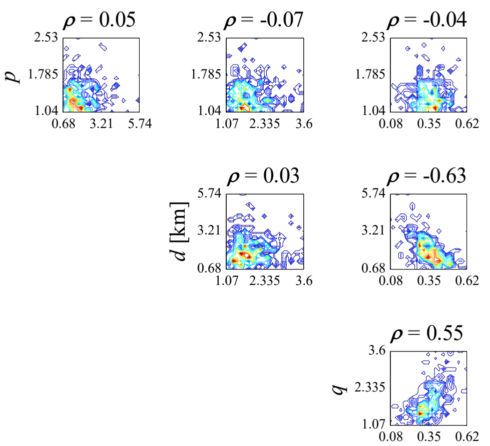

Correlation Matrix, [T_{start}=13/11-00:00, T_{end}=13/11-06:00];

	K	α	c	p	d	q	γ
β	-0.02	-0.06	-0.08	-0.05	0.01	-0.10	-0.09
K		0.00	-0.01	-0.10	-0.07	-0.09	-0.01
α			0.13	-0.20	-0.23	0.23	0.26
c				0.26	-0.06	0.14	0.13
p					0.05	-0.07	-0.04
d						0.03	-0.63
q							0.55

Figure SI-6: Contour lines representing the correlation between pairs of the posterior marginal samples of θ=[β, α, c, p, d, q, γ] for the second forecasting interval (see the 2nd row in Table 1) and the corresponding correlation matrix.

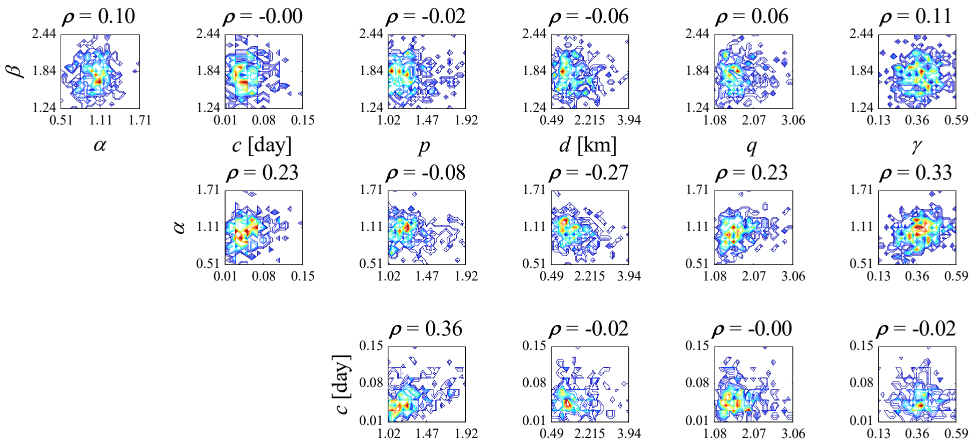

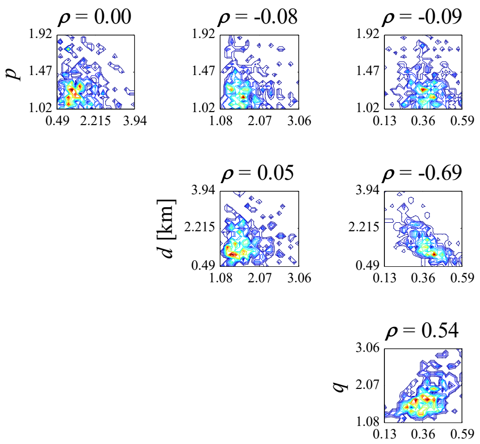

Correlation Matrix, [T_{start}=13/11-06:00, T_{end}=14/11-06:00];

	K	α	c	p	d	q	γ
β	-0.02	0.10	0.00	-0.02	-0.06	0.06	0.11
K		-0.03	-0.06	-0.16	-0.06	0.13	0.10
α			0.23	-0.08	-0.27	0.23	0.33
c				0.36	-0.02	0.00	-0.02
p					0.00	-0.08	-0.09
d						0.05	-0.69
q							0.54

Figure SI-7: Contour lines representing the correlation between pairs of the posterior marginal samples of **θ**=[β, α, c, p, d, q, γ] for the third forecasting interval (see the 3rd row in Table 1) and the corresponding correlation matrix.

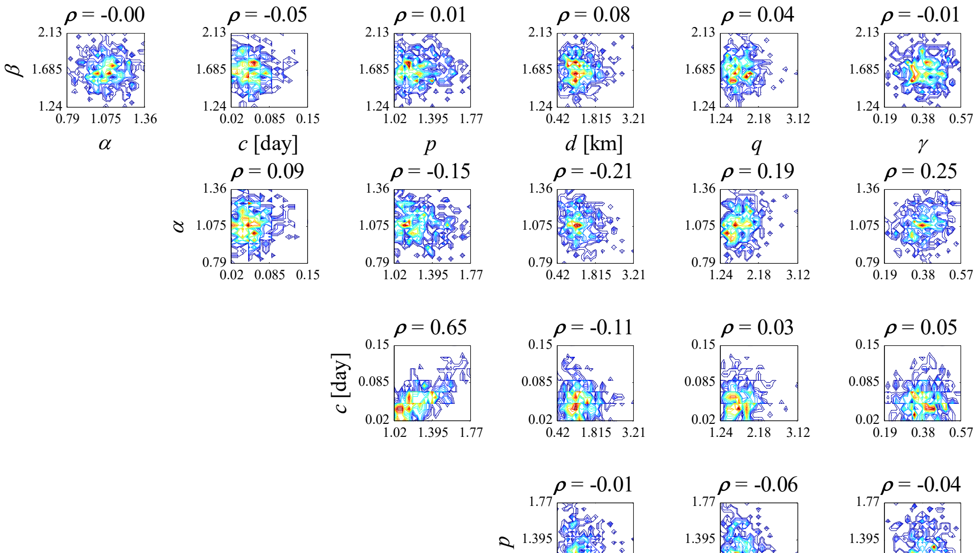

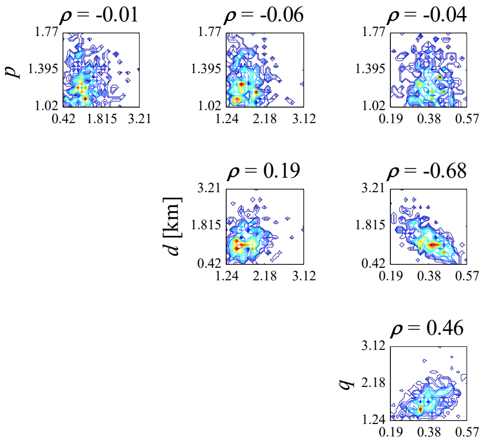

Correlation Matrix, [T_{start}=14/11-06:00, T_{end}=15/11-06:00];

	K	α	c	p	d	q	γ
β	0.03	0.00	-0.05	0.01	0.08	0.04	-0.01
K		-0.07	-0.25	-0.42	0.01	-0.06	-0.04
α			0.09	-0.15	-0.21	0.19	0.25
c				0.65	-0.11	0.03	0.05
p					-0.01	-0.06	-0.04
d						0.19	-0.68
q							0.46

Figure SI-8: Contour lines representing the correlation between pairs of the posterior marginal samples of θ=[β, α, c, p, d, q, γ] for the fourth forecasting interval (see the 4th row in Table 1) and the corresponding correlation matrix.

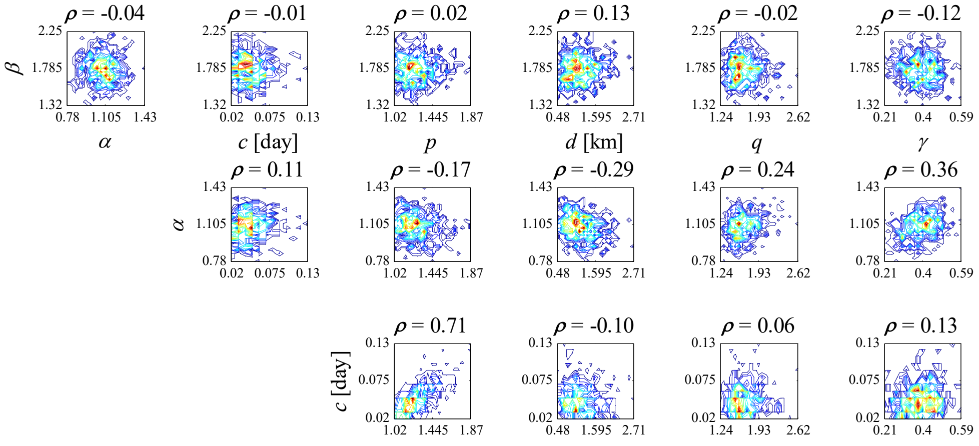

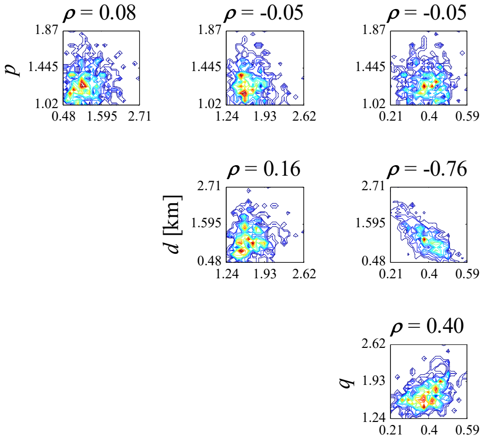

Correlation Matrix, [T_{start}=15/11-06:00, T_{end}=16/11-06:00];

	K	α	c	p	d	q	γ
β	-0.01	-0.04	-0.01	0.02	0.13	-0.02	-0.12
K		0.04	-0.08	-0.17	-0.02	0.06	0.05
α			0.11	-0.17	-0.29	0.24	0.36
c				0.71	-0.10	0.06	0.13
p					0.08	-0.05	-0.05
d						0.16	-0.76
q							0.40

Figure SI-9: Contour lines representing the correlation between pairs of the posterior marginal samples of $\theta=[\beta, \alpha, c, p, d, q, \gamma]$ for the fifth forecasting interval (see the 5th row in Table 1) and the corresponding correlation matrix.

.

SI-6. Forecasting Results for different time intervals within Phase 1

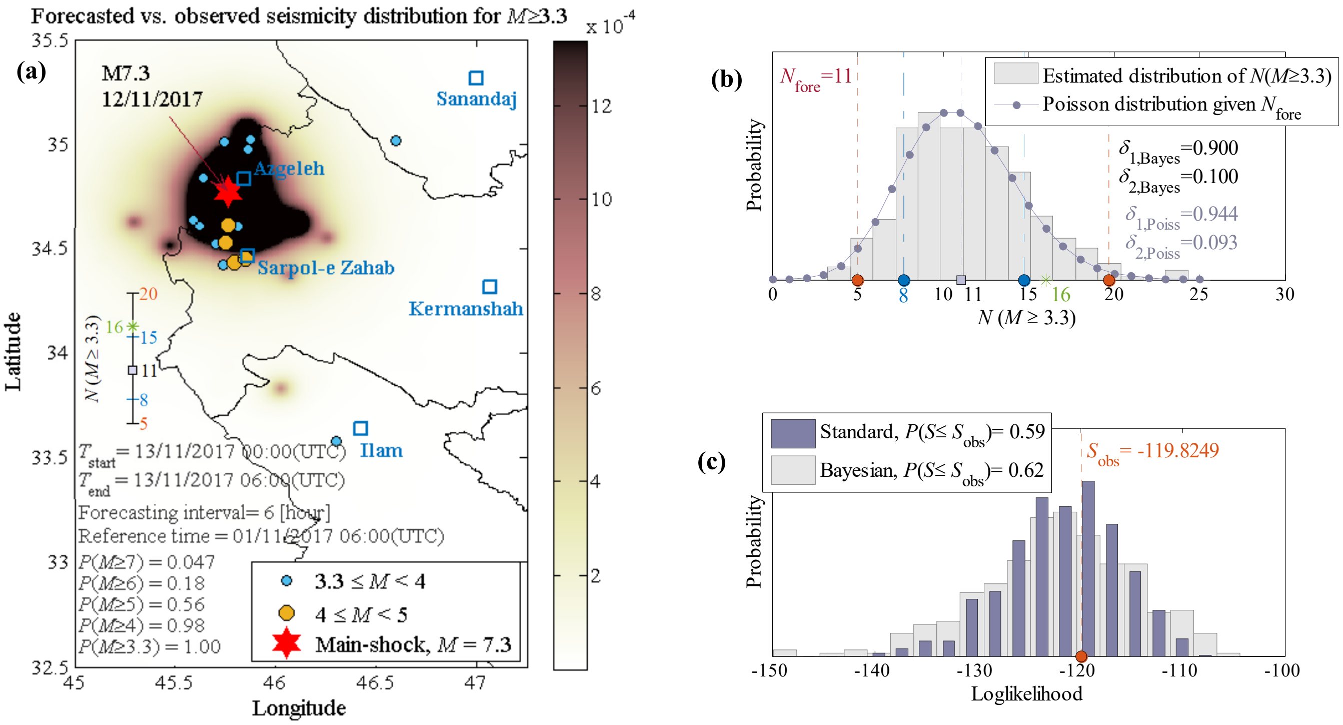

Figure SI-10: (a) The map showing forecasted vs. observed seismicity distribution in the aftershock zone for the 2nd forecasting time window [T_{start}=13/11/2017-00:00UTC, $Tend$=13/11/2017-06:00UTC] including: the expected value for the number of events in each cell unit with M≥3.30; the reported $P(M \geq m)$; the earthquakes that occurred during the corresponding forecasting time window; the mainshock of M_w7.3; and bar chart showing the observed vs. the percentiles of the forecasted number of events. (b) N-test based on the simulation-based Bayesian workflow and the Poisson distribution; consistent with the error-bar on the left-side of Figure SI-10(a), the green star shows the observed number of events within the forecasting interval (=16), the grey-filled square is the median value or the 50th percentile (=11); blue circles are the 16th and 84th percentiles (=8 & 15); red circles show the 2nd and 98th percentiles (=5 & 20); N_{fore} = 11 is the expected number of events $N(M \geq 3.30)$. (c) S-test based on the simulation-based Bayesian framework and the standard method.

Discussion on Figure SI-10: For this second early forecasting interval (5 hours and 42 minutes after the main event) in phase 1, the **seq** includes 27 events with M≥3.3. The observed number of events (=16) lies close to (+1) standard deviation of the mean estimate (=15). Both N-test and L-test show that the forcasts issued in terms of the number of events and the spatial distribution of seismicity are within the accepotable range. For the S-test, (Figure SI-10c), the probability $P(S \leq S_{obs})$ is more than 50% showing that the spatial distriution of the forecasts matches very well with the observed seismicity.

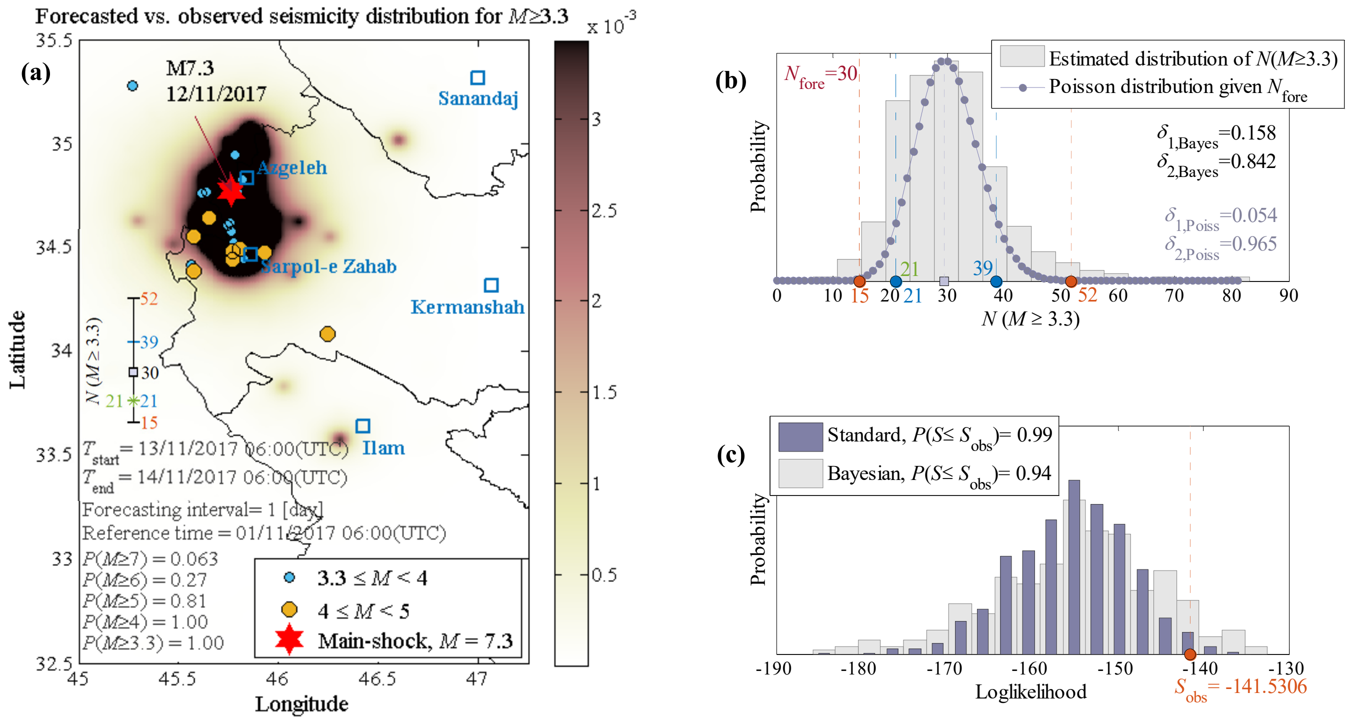

Figure SI-11: (a) The map showing forecasted vs. observed seismicity distribution in the aftershock zone for the 3rd forecasting time window [T_{start}=13/11/2017-06:00UTC, T_{end}=14/11/2017-06:00UTC] including: the expected value for the number of events in each cell unit with M≥3.30; the reported $P(M \geq m)$; the earthquakes that occurred during the corresponding forecasting time window; the mainshock of M_w7.3; and bar chart showing the observed vs. the percentiles of the forecasted number of events. (b) N-test based on the simulation-based Bayesian workflow and the Poisson distribution; consistent with the error-bar on the left-side of Figure SI-11(a), the green star shows the observed number of events within the forecasting interval (=21), the grey-filled square is the median value or the 50th percentile (=30); blue circles are the 16th and 84th percentiles (=21 & 39); red circles show the 2nd and 98th percentiles (=15 & 52); N_{fore} = 30 is the expected number of events $N(M \geq 3.30)$. (c) S-test based on the simulation-based Bayesian framework and the standard method.

Discussion on Figure SI-11: For this third early forecasting interval (around 12 hours after the main event) in phase 1, the **seq** includes 43 events with M≥3.3. It is noted that the observed number of events (=21; see also Figure 2) lies on (-1) standard deviation of the mean estimate (=21). Both N-test and L-test show that the forcasts issued in terms of the number of events and the spatial distribution of seismicity are within the accepotable range. For the L-test, (Figure SI-11c), the probability $P(S \leq S_{obs})$ is large showing that the spatial distriution of the simulated catalogs are less likely compared to the observed distribution.

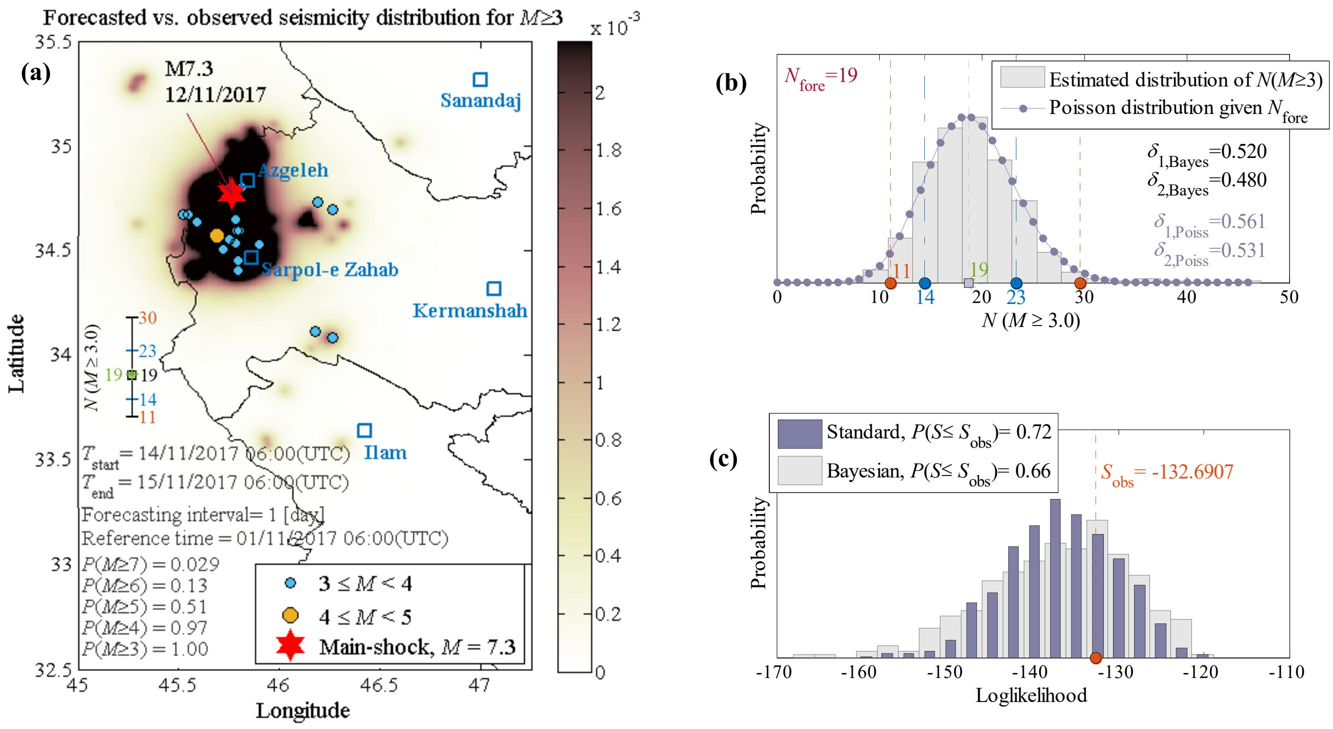

Figure SI-12: (a) The map showing forecasted vs. observed seismicity distribution in the aftershock zone for the 4th forecasting time window [T_{start}=14/11/2017-06:00UTC, T_{end}=15/11/2017-06:00UTC] including: the expected value for the number of events in each cell unit with M≥3.0; the reported $P(M≥m)$; the earthquakes that occurred during the corresponding forecasting time window; the mainshock of M_w7.3; and bar chart showing the observed vs. the percentiles of the forecasted number of events. (b) N-test based on the simulation-based Bayesian workflow and the Poisson distribution; consistent with the error-bar on the left-side of Figure SI-12(a), the green star shows the observed number of events within the forecasting interval (=19), the grey-filled square is the median value or the 50th percentile (=19); blue circles are the 16th and 84th percentiles (=14 & 23); red circles show the 2nd and 98th percentiles (=11 & 30); N_{fore} = 19 is the expected number of events $N(M≥3.0)$. (c) S-test based on the simulation-based Bayesian framework and the standard method.

Discussion on Figure SI-12: For this 4th forecasting interval (around 36 hours after the main event) in Phase 1, the **seq** includes 98 events with M≥3.0. The observed number of events (=19; see also Figure 2) is equal to the N_{fore} = 19. Both N-test and S-test show that the forcasts issued in terms of the number of events and the spatial distribution of seismicity matches perfectly the observed seimicity.

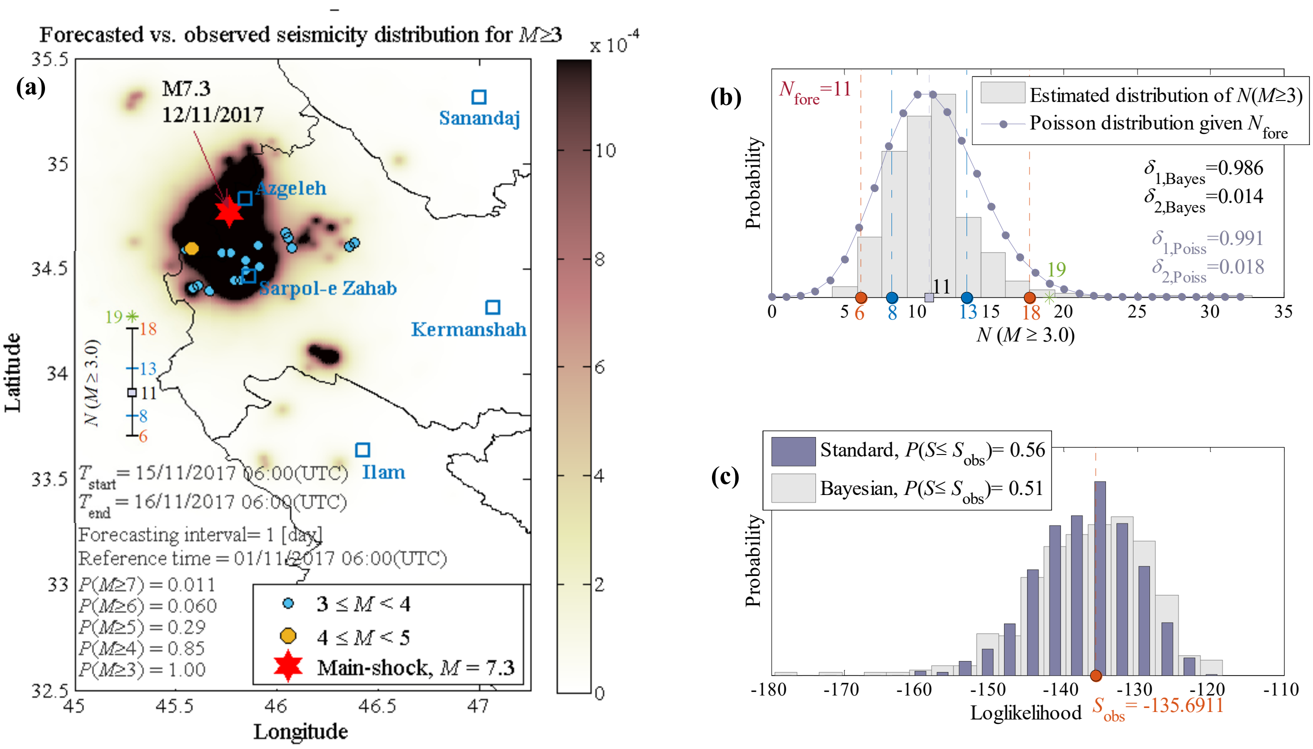

Figure SI-13: (a) The map showing forecasted vs. observed seismicity distribution in the aftershock zone for the 5th forecasting time window [T_{start}=15/11/2017-06:00UTC, T_{end}=16/11/2017-06:00UTC] including: the expected value for the number of events in each cell unit with M≥3.0; the reported $P(M≥m)$; the earthquakes that occurred during the corresponding forecasting time window; the mainshock of M_w7.3; and bar chart showing the observed vs. the percentiles of the forecasted number of events. (b) N-test based on the simulation-based Bayesian workflow and the Poisson distribution; consistent with the error-bar on the left-side of Figure SI-13(a), the green star shows the observed number of events within the forecasting interval (=19), the grey-filled square is the median value or the 50th percentile (=11); blue circles are the 16th and 84th percentiles (=8 & 13); red circles show the 2nd and 98th percentiles (=6 & 18); N_{fore} = 11 is the expected number of events $N(M≥3.0)$. (c) S-test based on the simulation-based Bayesian framework and the standard method.

Discussion on Figure SI-13: For this 5th forecasting interval (around 60 hours after the main event) in phase 1, the **seq** includes 117 events with M≥3.0. It can be seen that observed number of events (=19; see also Figure 2) lies close to (+2) standard deviation of the mean estimate (=18). Both N-test shows that the forcasts issued in terms of the number of events is not acceptable, while S-test reveals that the spatial distribution of seismicity is predicted very well, i.e., $P(S\leq S_{\text{obs}})$ is around 50%. With reference to Figure 2, while the number of events with M≥3.0 in this day does not change with respect to the previous day (=19), the number of events with M≥3.3 is increased. This shows a sign of triggered seismicity within this day.

SI-7. Plots for estimating the completeness magnitude M_c for different forecasting intervals in phase 3 of the seismic sequence

The graphical representation for finding M_c based on first two methods described in Section 4.2.1 of the manuscript for various forecasting intervals right after the occurrence of the Sarpol-e Sahab event with M_w6.3 is shown in Figure SI-14 to Figure SI-17.

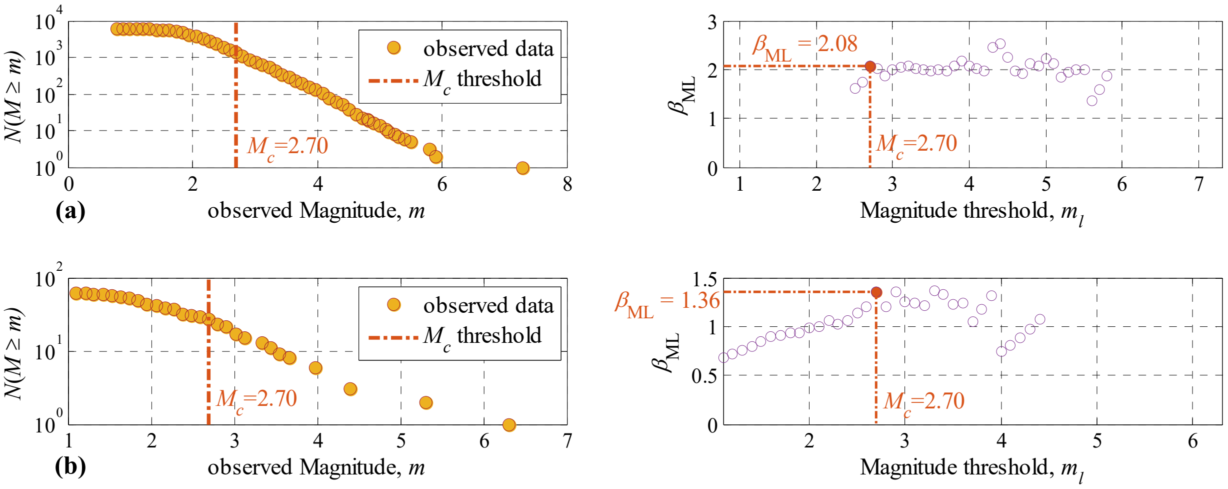

Figure SI-14: Visual presentation of two strategies introduced in Section 4.2.1 (*Methods*) for estimation/validation of M_c considering the events in the time intervals (a) [old T_o=01/11/2017-06:00 UTC – new T_o=20/11/2018-00:00 UTC], (b) [new T_o=20/11/2018-00:00 UTC, T_{start}=25/11/2018-18:00 UTC], in phase 3 of the seismic sequence

Discussion on Figure SI-14: Figure SI-14a shows two different strategies for estimating M_c for the events within the aftershock zone in the time interval of one year [old T_o=01/11/2017-06:00 UTC – new T_o=20/11/2018-00:00 UTC]. These two methods, presented in Section SI-3-Results (*Supplementary Information* ; see also the original manuscript Section 2.1.1 and Section 4.1), include: the frequency-magnitude semi-logarithmic plot (left plot), and the use of Bayesian inference to find the change in the maximum likelihood of the posterior probability distribution of the slope of regression (right plot). Selection of $M_c = 2.70$ meets the requirements of both methods especially in light of the Bayesian approach (shown in right plot). At the lower threshold of $m_l = 2.70$, we have reached the maximum estimate for β_{ML} value. It is noted that β_{ML} estimated through the Bayesian inference versus various magnitude thresholds m_l fails to provide the result for $m_l < 2.50$ as the slope of regression becomes very close to zero. On the same page, and based on the new T_o, Figures SI-14b illustrates the visual presentation of the two aforementioned methods for estimating M_c for the events within the aftershock zone and in the time interval of 5 days [new T_o=20/11/2018-00:00 UTC, T_{start}=25/11/2018-18:00 UTC]. The data within this time interval forms the **seq** for the first forecast issued with T_{start} = 25/11/2018-18:00 UTC (i.e., 1 hour and 23 minutes after the occurrence of M_w6.3 event, as discussed in Section 2.2.2). It can be seen that $M_c = 2.70$ is a proper choice also for this time interval in order to issue forecasts starting at T_{start}. The selection of $M_c = 2.70$ leads to $\beta_{\mathrm{ML}} = 2.08$ in Figure SI-14a (medium term) and $\beta_{\mathrm{ML}} = 1.36$ (short term) in Figure SI-14b.

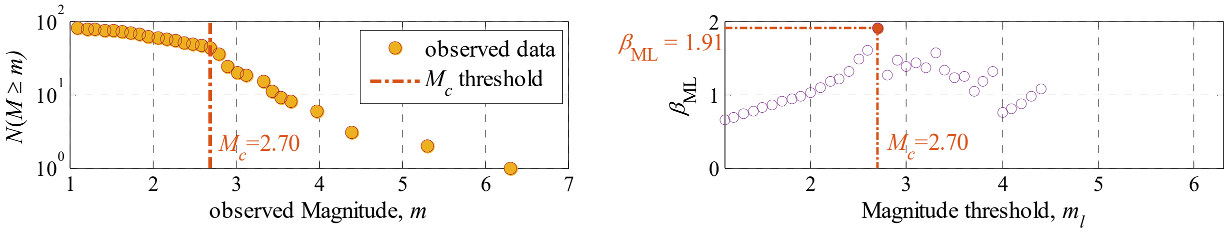

Figure SI-15: Visual presentation of two strategies introduced in Section 4.2.1 (*Methods*) for estimation of M_c for the forecasting interval [25/11/2018-19:00UTC, 26/11/2018-00:00UTC] in phase 3 of the seismic sequence

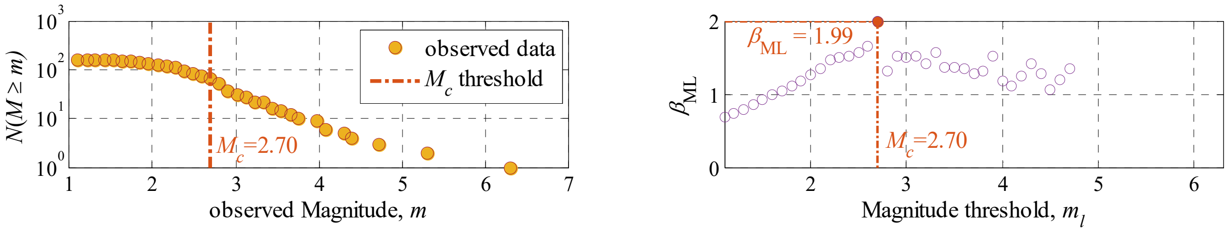

Figure SI-16: Visual presentation of two strategies introduced in Section 4.2.1 (*Methods*) for estimation of M_c for the forecasting interval [26/11/2018-00:00UTC, 26/11/2018-06:00UTC] in phase 3 of the seismic sequence

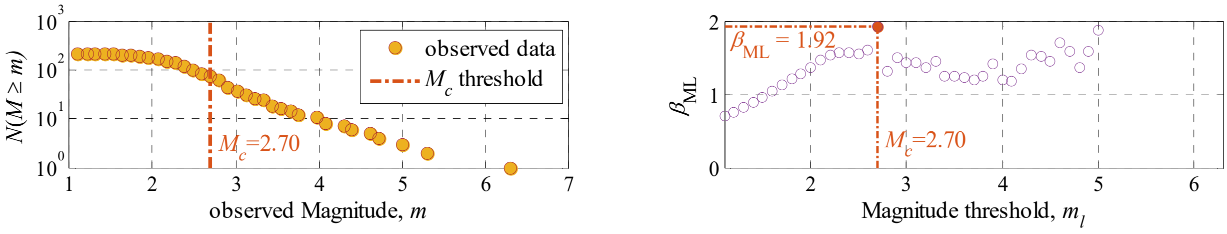

Figure SI-17: Visual presentation of two strategies introduced in Section 4.2.1 (*Methods*) for estimation of M_c for the forecasting interval [26/11/2018-06:00UTC, 27/11/2018-00:00UTC] in phase 3 of the seismic sequence

SI-8. Distribution of ETAS model parameters for Phase 3 of the seismic sequence

Table SI-2: Distribution of the ETAS model parameters including marginal PDFs of posterior (shown with bar plots) and lognormal PDFs of prior (shown with dashed orange lines), and the statistics of posterior (including mean and [2%-98%] confidence interval (CI) for providing early seismicity forecasts in Phase 3 of the seismic sequence triggered by M_w 6.3 at 25-November 2018

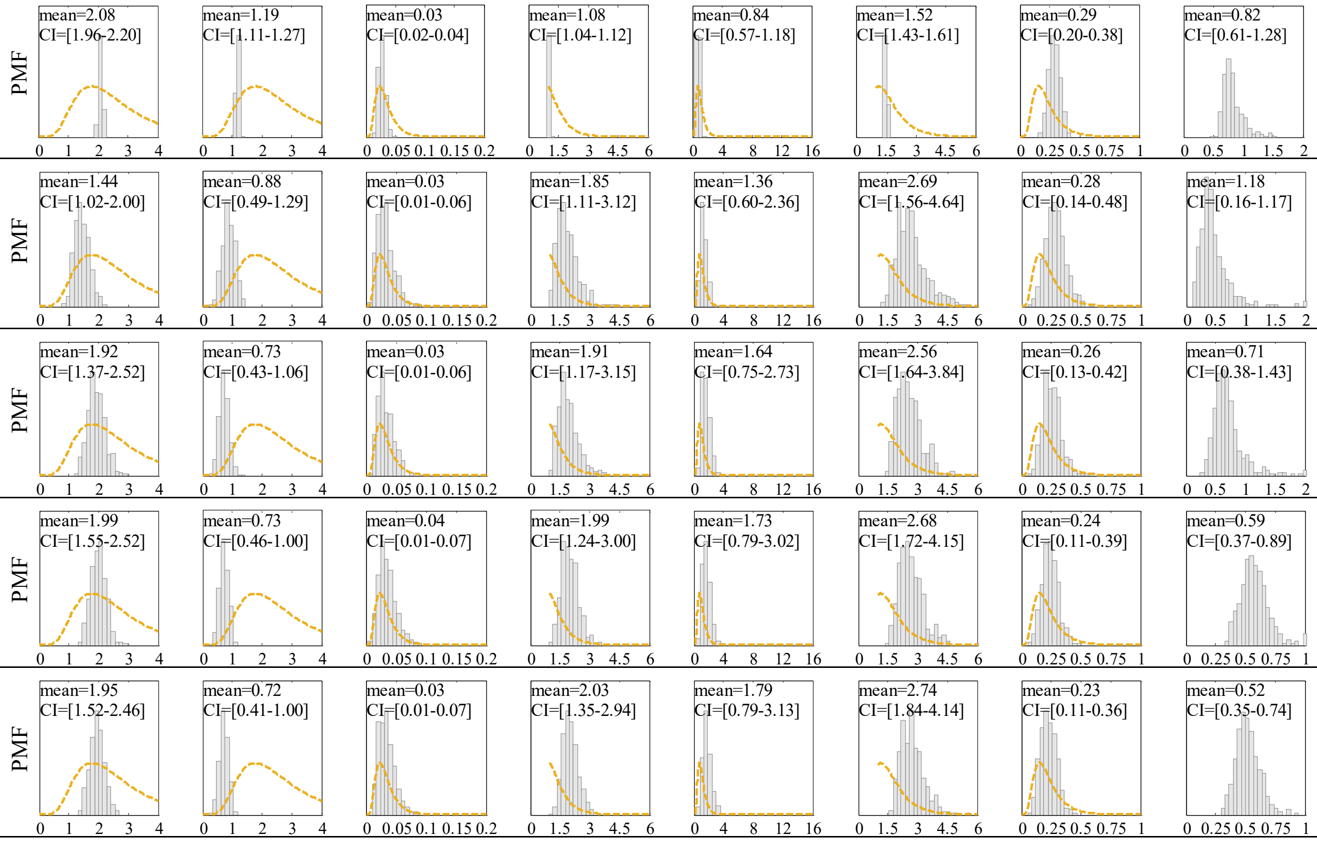

$[T_{start}, T_{end}]$* (dd/mm-hour)	β	α	c [day]	p	d [km]	q	γ	K
(a) ** [20/11-00:00, 21/11-00:00]; $N_d = 410$ $M_l = 2.70$	mean=2.08 CI=[1.96-2.20]	mean=1.19 CI=[1.11-1.27]	mean=0.03 CI=[0.02-0.04]	mean=1.08 CI=[1.04-1.12]	mean=0.84 CI=[0.57-1.18]	mean=1.52 CI=[1.43-1.61]	mean=0.29 CI=[0.20-0.38]	mean=0.82 CI=[0.61-1.28]
(b) [25/11-18:00, 26/11-00:00]; $N_d = 375$ $M_l = 2.70$	mean=1.44 CI=[1.02-2.00]	mean=0.88 CI=[0.49-1.29]	mean=0.03 CI=[0.01-0.06]	mean=1.85 CI=[1.11-3.12]	mean=1.36 CI=[0.60-2.36]	mean=2.69 CI=[1.56-4.64]	mean=0.28 CI=[0.14-0.48]	mean=1.18 CI=[0.16-1.17]
(c) [25/11-19:00, 26/11-00:00]; $N_d = 375$ $M_l = 2.70$	mean=1.92 CI=[1.37-2.52]	mean=0.73 CI=[0.43-1.06]	mean=0.03 CI=[0.01-0.06]	mean=1.91 CI=[1.17-3.15]	mean=1.64 CI=[0.75-2.73]	mean=2.56 CI=[1.64-3.84]	mean=0.26 CI=[0.13-0.42]	mean=0.71 CI=[0.38-1.43]
(d) [26/11-00:00, 26/11-06:00]; $N_d = 353$ $M_l = 2.70$	mean=1.99 CI=[1.55-2.52]	mean=0.73 CI=[0.46-1.00]	mean=0.04 CI=[0.01-0.07]	mean=1.99 CI=[1.24-3.00]	mean=1.73 CI=[0.79-3.02]	mean=2.68 CI=[1.72-4.15]	mean=0.24 CI=[0.11-0.39]	mean=0.59 CI=[0.37-0.89]
(e) [26/11-06:00, 27/11-00:00]; $N_d = 401$ $M_l = 2.70$	mean=1.95 CI=[1.52-2.46]	mean=0.72 CI=[0.41-1.00]	mean=0.03 CI=[0.01-0.07]	mean=2.03 CI=[1.35-2.94]	mean=1.79 CI=[0.79-3.13]	mean=2.74 CI=[1.84-4.14]	mean=0.23 CI=[0.11-0.36]	mean=0.52 CI=[0.35-0.74]
Statistics of prior marginal PDFs	median=2.10 COV=0.50	median=1.20 COV=0.50	median=0.03 COV=0.50	median=1.10 COV=0.50	median=1.00 COV=0.50	median=1.50 COV=0.50	median=0.30 COV=0.50	–

* The time window denotes the *forecasting interval*; note that "hour" is based on UTC.

** The reference time for this interval is 01/11/2017-06:00 UTC (old T_o). This interval is before the occurrence of the main event of M_w 6.3 at 25/11/2018.

SI-9. Correlation structure among posteriors of the ETAS model parameters in Phase 3

Correlation Matrix, [T_{start} = new T_o= 20/11/2018-00:00, T_{end}=21/11/2018-00:00]; the **seq** consists of all the events with $M \geq M_l = 2.7$ that took place in the interval of [old T_o – new T_o]

	K	α	c	p	d	q	γ
β	-0.02	-0.08	0.00	0.01	-0.04	0.00	0.04
K		-0.23	-0.66	-0.89	-0.06	0.01	0.05
α			0.17	-0.03	-0.31	0.18	0.38
c				0.73	-0.02	-0.01	0.01
p					0.10	-0.08	-0.12
d						0.20	-0.89
q							0.20

Discussion on the correlations between pairs of model parameters **θ** *based on the 1-year seismicity:*

The correlations between the pairs of ETAS parameters, learnt through (around) 1-year **seq** data as descried above, are reported in the *Supplementary Information* (Section SI-6-Results; first correlation matrix). Thus, it can be a representative of the correlation structure in a larger time span. It is seen that β has almost no correlation with other ETAS parameters. K reveals a moderate correlation with α and high correlation with temporal parameters (c, p). This observation shows that K in long term is not affected by the spatial parameters. Parameter α has correlation with temporal parameter c (and not with p as was observed previously in early forecasts in Phase 1), and to a higher extent with the spatial parameters (d, q, γ). There is a high correlation between the temporal parameters (c, p), and significant negative correlation between the spatial parameters (d, γ); however, the high positive correlation between (q, γ), as observed in the previous early estimates in Phase 1, does not exist herein.

Discussion on the correlations between pairs of model parameters **θ** in Phase 3:

The correlation structure between the pairs of the ETAS model parameters for the first forecasting interval in Phase 3 [T_{start} =25/11/2018-18:00UTC, T_{end} =26/11/2018-00:00UTC] (Section 2.2.2) are shown in the *Supplementary Information* (Section SI-9-Results, second correlation matrix). There are only moderate correlations between the temporal parameters (c, p), high negative correlation between spatial parameters (d, γ), and moderate positive correlation between (q, γ). This shows that the parameter K and α (affecting the number of forecasted events) have low correlations with each other as well as other model parameters, which can be seen as evidence that the seismicity forecast for this time interval was not accepted through N-test and S-test. For the second forecasting interval [T_{start} =25/11/2018-19:00UTC, T_{end}=26/11/2018-00:00UTC] (Section 2.2.3), and the last two forecasting intervals [T_{start} =26/11/2018-00:00UTC, T_{end} =26/11/2018-06:00UTC] and [T_{start} =26/11/2018-06:00UTC, T_{end} =27/11/2018-00:00UTC] (see Section 2.2.4), the correlations between pairs of the ETAS model parameters are shown in Section SI-9-Results (third to fifth correlation matrix). Compared to the previous forecast, it is interesting to note that parameter K reveals correlations with α and the two temporal parameters (c, p); however, α still does not reveal any correlation with other ETAS parameters. This is not in line with the observations in Phase 1, and it may be attributed to the presence of higher background seismicity level employed in current phase. There is also high correlation between (c, p), and between pairs of the three spatial parameters (d, q, γ). It is noted that the correlations between (d, q) for different forecasting intervals in Phase 3 are higher compared to those in Phase 1. Again, this may be attributed to the nonhomogeneous background seismicity used in this phase of forecasting.

Correlation Matrix, [T_{start}=25/11-18:00, T_{end}=26/11-00:00];

	K	α	c	p	d	q	γ
β	-0.05	-0.02	0.05	0.03	-0.02	0.04	0.09
K		0.05	-0.07	-0.14	-0.03	0.08	0.06
α			0.09	-0.18	-0.03	0.09	0.10
c				0.36	-0.11	-0.10	0.00
p					0.03	-0.20	-0.17
d						0.23	-0.63
q							0.43

Correlation Matrix, [T_{start}=25/11-19:00, T_{end}=26/11-00:00];

	K	α	c	p	d	q	γ
β	-0.03	0.08	-0.06	-0.04	0.01	0.06	0.03
K		-0.35	-0.28	-0.55	-0.01	0.01	0.03
α			0.09	-0.18	-0.02	0.05	0.06
c				0.60	0.05	0.03	-0.08
p					0.01	-0.07	-0.12
d						0.50	-0.61
q							0.21

Correlation Matrix, [T_{start}=26/11-00:00, T_{end}=26/11-06:00];

	K	α	c	p	d	q	γ
β	-0.02	0.05	0.03	0.06	-0.03	0.01	0.00
K		-0.23	-0.18	-0.34	0.03	0.03	-0.02
α			-0.03	-0.16	-0.06	0.01	0.03
c				0.72	-0.18	-0.11	0.07
p					-0.14	-0.08	0.11
d						0.54	-0.56
q							0.23

Correlation Matrix, [T_{start}=26/11-06:00, T_{end}=27/11-00:00];

	K	α	c	p	d	q	γ
β	-0.04	0.02	0.09	0.07	0.07	-0.05	-0.08
K		-0.80	-0.37	-0.44	-0.03	-0.03	-0.02
α			0.16	0.03	0.02	0.10	-0.01
c				0.75	-0.03	-0.01	0.03
p					0.00	-0.09	0.02
d						0.53	-0.62
q							0.15

SI-10. Forecasting Results for different time intervals within Phase 1

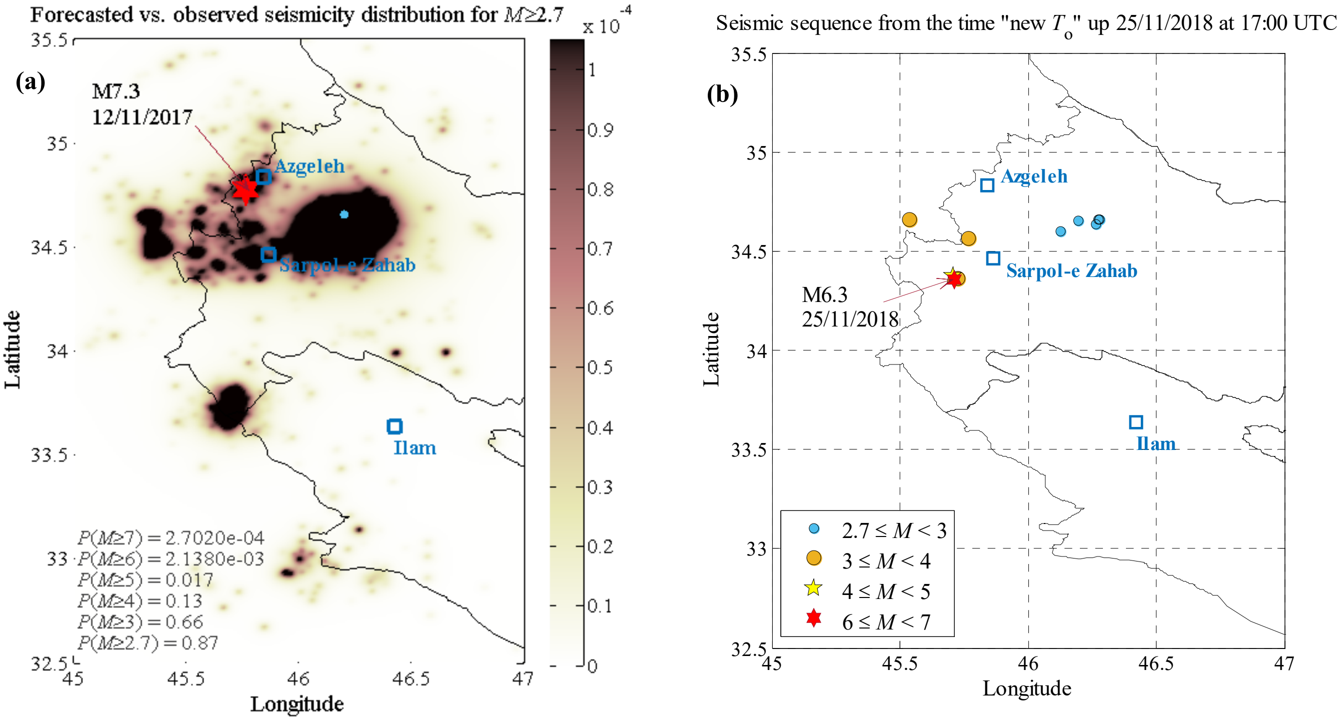

Figure SI-18: (a) The distribution of updated background seismicity $\mu(x, y|M_l)$ within the aftershock zone for Phase 3 starting from new T_o; (b) seismic activity before the occurrence of Sarpol-e Zahab earthquake with M_w6.3 starting from new T_o up to 25/11/2018-17:00 UTC

Discussion on Figure SI-18: Figure SI-18a shows the distribution of the background seismicity within the aftershock zone at new T_o associated with Phase 3. Obviously, this new background seismicity also includes the long-term seismicity used in Phase 1. To have a measure of the increased seismicity in the interval of [old T_o – new T_o], the expected number of events with $M \geq 2.7$, $\mathbb{E}[N(M \geq 2.70|\boldsymbol{\theta}, \mathbf{seq}, M_l = 2.7)] \cong 2$ (see Equation 12), can be interpreted as the daily rate of seismicity for $M \geq M_l$. It is noted that the real number of events with $M \geq 2.7$ in the one-day time interval is equal to one (see the dotted blue circle in Figure SI-18a), which is within the confidence interval of the forecasted seismicity. The daily base seismicity estimated from the long-term seismic activity in the aftershock zone for $M \geq M_l = 2.7$ (at time old T_o=01/11/2017-06:00 UTC) is estimated to be $\frac{2.301}{365} = 0.0063$ (see Table SI-1). Thus, the background seismicity has increased more than 300 times. On the same page, it is interesting to see the forecasted seismicity through the exceedance daily probability for $P(M \geq 6) = 0.0021$, as shown in Figure SI-18a. We are interested in $M \geq 6$ as the Sarpol-e Zahab event of M_w6.3 took place five days after new T_o (see Figure 6). From the calculation of the long-term seismicity level, the estimated daily probability is around $1 - \exp(-(6.1239 \times 10^{-2})/365) = 1.68 \times 10^{-4}$ (see Table SI-1 where the rate of events from long-term background seismicity with $M \geq 6$ is 6.1239×10^{-2}). Hence, the level of (forecasted) seismicity for probability of occurrence of $M \geq 6$ at the desired date is more than 12 times higher than the base seismicity level, which reveals an alarming level. Figure SI-18b illustrates all the events with $M \geq M_l = 2.7$ starting from new T_o up 25/11/2018 at 17:00 UTC (right after the M_w6.3 at 16:37 UTC. Within this time interval, there are 11 events in two different clusters. A set of 6 seismic events with $2.7 \leq M < 3.0$ (blue-colored circles) are clustered in the right side showing the continuation of the M_w5.9 (Tazehabad event) in Phase 2 of the seismic sequence. The three seismic events with $3.0 \leq M < 4.0$ (orange-colored circles) are taken place close to the epicenter of M_w6.3 that is shown with red-colored hexagram (these events can also

be traced in Figure 7). It is noted that among the three events, the closest one with $M_w3.1$ took place less than three hours before the main event (i.e., the main event was preceded by only one foreshock in the same date). The yellow-colored pentagram shows the first aftershock with $M_w4.4$ that took place right after the $M_w6.3$ main event. With reference to the **seq**, a total of 16 triggered aftershocks occurred in 1-hour time interval from 17:00 UTC up to 18:00 UTC (i.e., the time of T_{start}). The aftershock data also features the triggered event of $M_w5.3$ at 17:09 UTC.

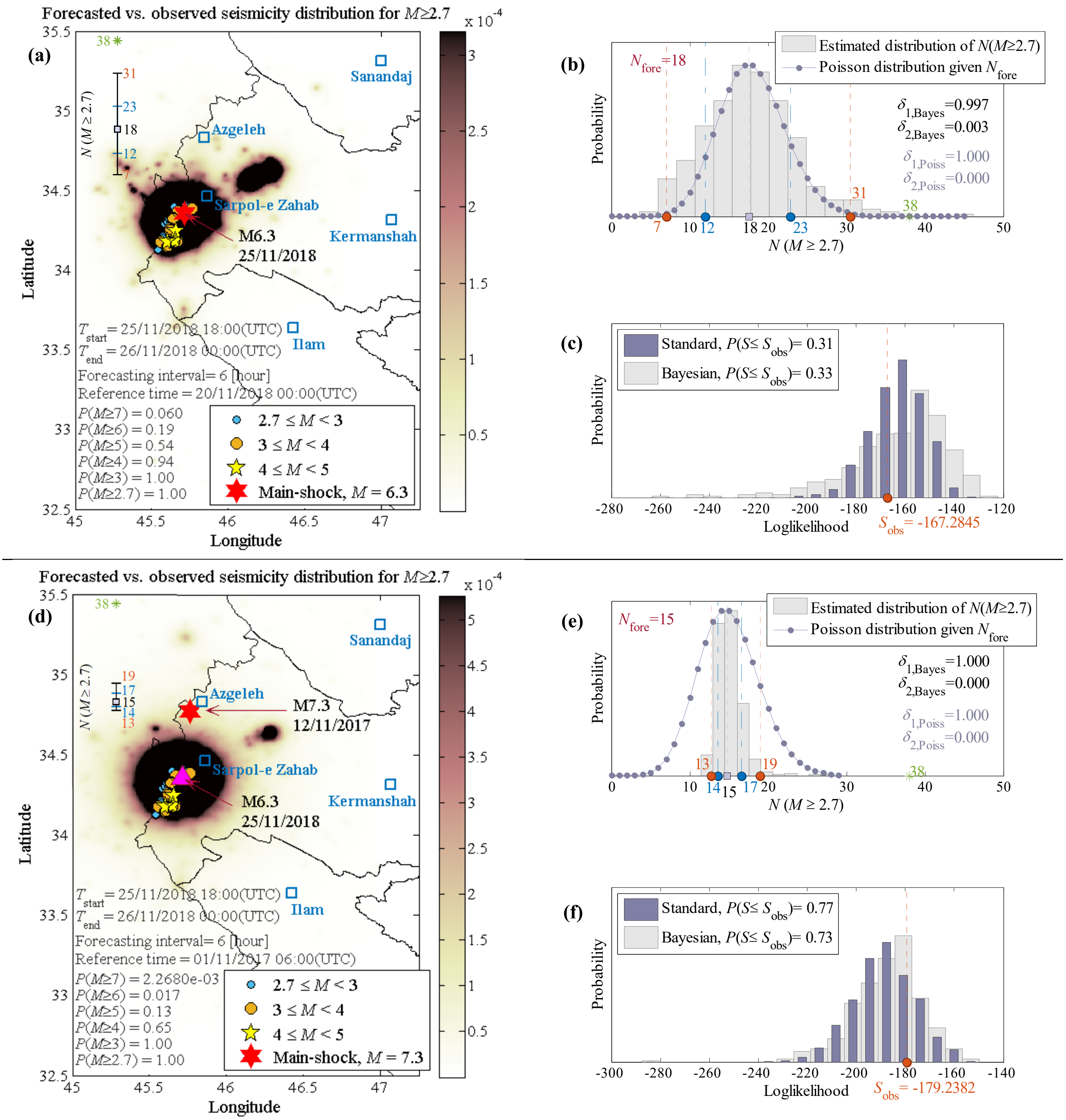

Figure SI-19: (a) The map showing forecasted vs. observed seismicity distribution in the aftershock zone for the forecasting time window [T_{start}=25/11/2018-18:00UTC, T_{end}=26/11/2018-00:00UTC] with the reference time of "new T_o" including: the expected value for the number of events in each cell unit with M≥2.70; the reported $P(M\geq m)$; the earthquakes that occurred during the corresponding forecasting time window; the main event of M_w6.3; and bar chart showing the observed vs. the percentiles of the forecasted number of events. (b) N-test associated with the forecast issued in subplot "a" based on the simulation-based Bayesian workflow and the Poisson distribution; consistent with the error-bar on the left-side, the green star shows the observed number of

events within the forecasting interval (=38), the grey-filled square is the median value or the 50th percentile (=18); blue circles are the 16th and 84th percentiles (=12 & 23); red circles show the 2nd and 98th percentiles (=7 & 31); N_{fore} = 18 is the expected number of events $N(M \geq 2.70)$. (c) S-test associated with the forecast issued in subplot "a" based on the simulation-based Bayesian framework and the standard method. (d) The same map as the subplot "a" but with the refrence time of "old T_o" (e) N-test associated with the forecast issued in subplot "d" consistent with the error-bar on the left-side with the median value or the 50th percentile (=15); the 16th and 84th percentiles (=14 & 17); the 2nd and 98th percentiles (=13 & 18); N_{fore} = 15. (f) S-test associated with the forecast issued in subplot "d".

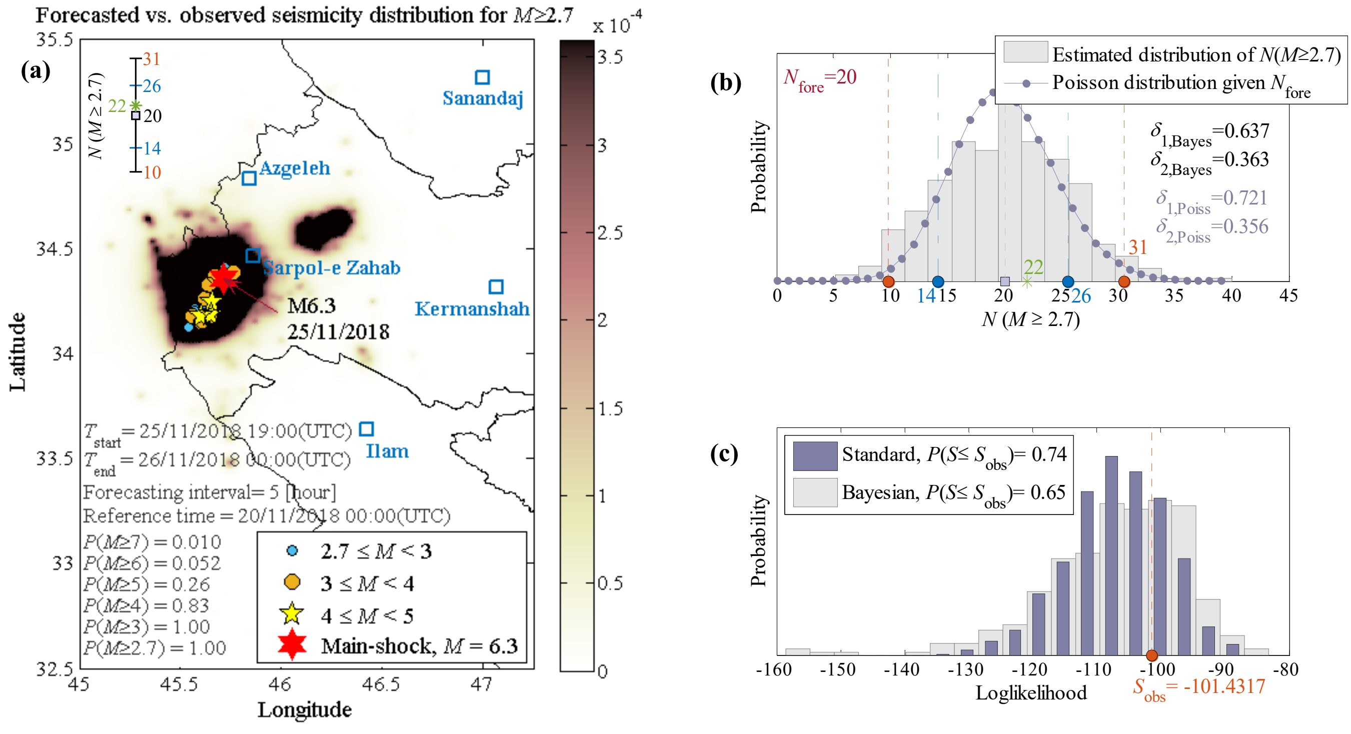

Figure SI-20: (a) The map showing forecasted vs. observed seismicity distribution in the aftershock zone for the forecasting time window [T_{start} =25/11/2018-19:00UTC, T_{end} =26/11/2018-00:00UTC] including: the expected value for the number of events in each cell unit with $M \geq 2.70$; the reported $P(M \geq m)$; the earthquakes that occurred during the corresponding forecasting time window; the main event of M_w6.3; and bar chart showing the observed vs. the percentiles of the forecasted number of events. (b) N-test based on the simulation-based Bayesian workflow and the Poisson distribution; consistent with the error-bar on the left-side, the green star shows the observed number of events within the forecasting interval (=22), the grey-filled square is the median value or the 50th percentile (=20); blue circles are the 16th and 84th percentiles (=14 & 26); red circles show the 2nd and 98th percentiles (=10 & 31); N_{fore} = 20 is the expected number of events $N(M \geq 2.70)$. (c) S-test based on the simulation-based Bayesian framework and the standard method.

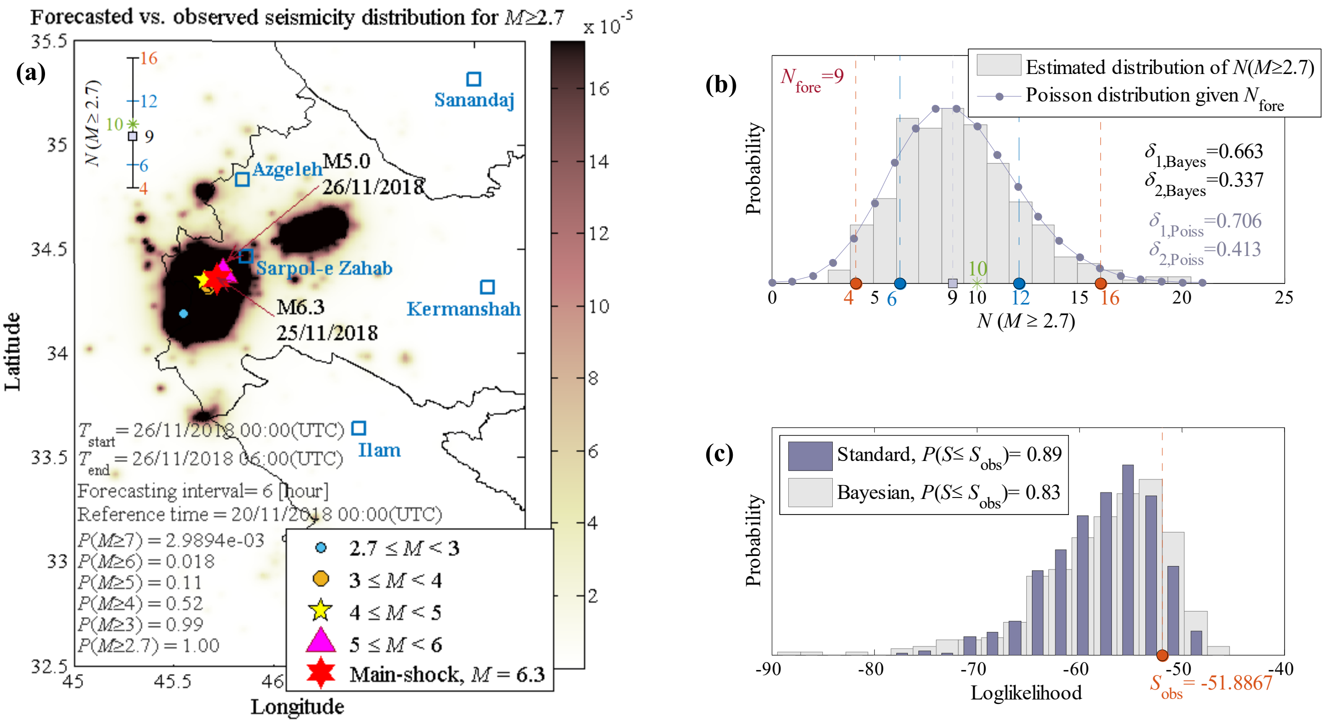

Figure SI-21: (a) The map showing forecasted vs. observed seismicity distribution in the aftershock zone for the forecasting time window [T_{start} =26/11/2018-00:00UTC, T_{end} =26/11/2018-06:00UTC] including: the expected value for the number of events in each cell unit with M≥2.70; the reported $P(M≥m)$; the earthquakes that occurred during the corresponding forecasting time window; the main event of M_w6.3; and bar chart showing the observed vs. the percentiles of the forecasted number of events. (b) N-test based on the simulation-based Bayesian workflow and the Poisson distribution; consistent with the error-bar on the left-side, the green star shows the observed number of events within the forecasting interval (=10), the grey-filled square is the median value or the 50th percentile (=9); blue circles are the 16th and 84th percentiles (=6 & 12); red circles show the 2nd and 98th percentiles (=4 & 16); N_{fore} = 9 is the expected number of events $N(M≥2.70)$. (c) S-test based on the simulation-based Bayesian framework and the standard method.

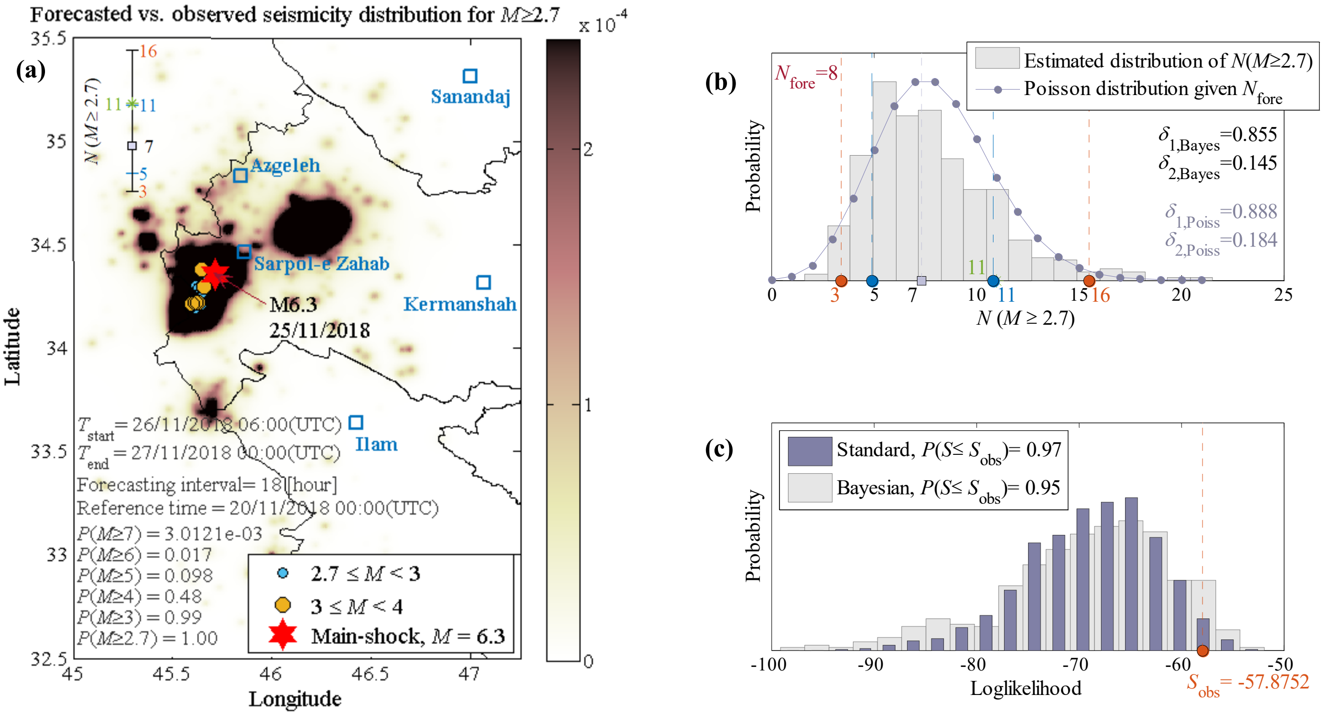

Figure SI-22: (a) The map showing forecasted vs. observed seismicity distribution in the aftershock zone for the forecasting time window [T_{start} =26/11/2018-06:00UTC, T_{end} =27/11/2018-00:00UTC] including: the expected value for the number of events in each cell unit with M≥2.70; the reported $P(M≥m)$; the earthquakes that occurred during the corresponding forecasting time window; the main event of M_w6.3; and bar chart showing the observed vs. the percentiles of the forecasted number of events. (b) N-test based on the simulation-based Bayesian workflow and the Poisson distribution; consistent with the error-bar on the left-side, the green star shows the observed number of events within the forecasting interval (=11), the grey-filled square is the median value or the 50th percentile (=7); blue circles are the 16th and 84th percentiles (=5 & 11); red circles show the 2nd and 98th percentiles (=3 & 16); N_{fore} = 8 is the expected number of events $N(M≥2.70)$. (c) S-test based on the simulation-based Bayesian framework and the standard method.

SI-11. Posterior distribution of the ETAS model parameters based on simple spatial kernel density formulation together with the correlation structure

Table SI-3: Distribution of the ETAS model parameters based on *simple spatial kernel density* model including marginal PDFs of posterior (shown with bar plots) and lognormal PDFs of prior (shown with dashed orange lines), and the statistics of posterior (including mean and [2%-98%] confidence interval (CI) for providing early seismicity

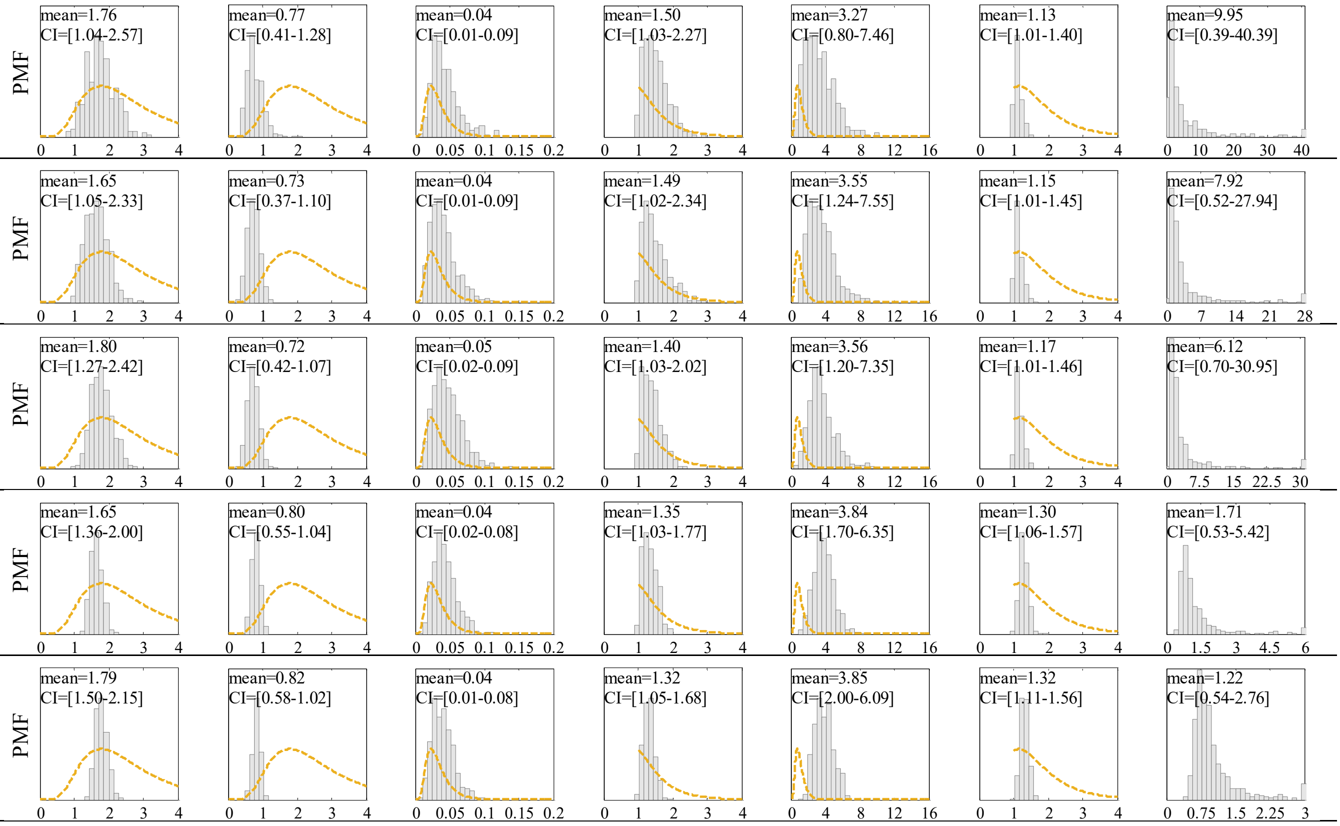

$[T_{start}, T_{end}]^*$ (dd/mm-hour)	β	α	c [day]	p	d [km]	q	K
(a) [12/11-21:00, 13/11-06:00]; $N_d = 365$ $M_l = 3.40$	mean=1.76 CI=[1.04-2.57]	mean=0.77 CI=[0.41-1.28]	mean=0.04 CI=[0.01-0.09]	mean=1.50 CI=[1.03-2.27]	mean=3.27 CI=[0.80-7.46]	mean=1.13 CI=[1.01-1.40]	mean=9.95 CI=[0.39-40.39]
(b) [13/11-00:00, 13/11-06:00]; $N_d = 449$ $M_l = 3.30$	mean=1.65 CI=[1.05-2.33]	mean=0.73 CI=[0.37-1.10]	mean=0.04 CI=[0.01-0.09]	mean=1.49 CI=[1.02-2.34]	mean=3.55 CI=[1.24-7.55]	mean=1.15 CI=[1.01-1.45]	mean=7.92 CI=[0.52-27.94]
(c) [13/11-06:00, 14/11-06:00]; $N_d = 424$ $M_l = 3.30$	mean=1.80 CI=[1.27-2.42]	mean=0.72 CI=[0.42-1.07]	mean=0.05 CI=[0.02-0.09]	mean=1.40 CI=[1.03-2.02]	mean=3.56 CI=[1.20-7.35]	mean=1.17 CI=[1.01-1.46]	mean=6.12 CI=[0.70-30.95]
(d) [14/11-06:00, 15/11-06:00]; $N_d = 490$ $M_l = 3.00$	mean=1.65 CI=[1.36-2.00]	mean=0.80 CI=[0.55-1.04]	mean=0.04 CI=[0.02-0.08]	mean=1.35 CI=[1.03-1.77]	mean=3.84 CI=[1.70-6.35]	mean=1.30 CI=[1.06-1.57]	mean=1.71 CI=[0.53-5.42]
(e) [15/11-06:00, 16/11-06:00]; $N_d = 471$ $M_l = 3.00$	mean=1.79 CI=[1.50-2.15]	mean=0.82 CI=[0.58-1.02]	mean=0.04 CI=[0.01-0.08]	mean=1.32 CI=[1.05-1.68]	mean=3.85 CI=[2.00-6.09]	mean=1.32 CI=[1.11-1.56]	mean=1.22 CI=[0.54-2.76]
Statistics of prior marginal PDFs	median=2.30 COV=0.50	median=2.30 COV=0.50	median=0.03 COV=0.50	median=1.10 COV=0.50	median=1.00 COV=0.50	median=1.50 COV=0.50	–

*The time window denotes the *forecasting interval*; note that "hour" is based on UTC.

Correlation Matrix, $[T_{start}$=12/11-21:00, T_{end}=13/11-06:00];

	K	α	c	p	d	q
β	0.02	0.00	0.05	-0.05	-0.02	-0.01
K		-0.11	0.00	-0.19	-0.05	-0.14
α			0.09	-0.04	0.06	-0.02
c				0.07	0.02	-0.04
p					-0.13	-0.06
d						0.52

Correlation Matrix, [T_{start}=13/11-00:00, T_{end}=13/11-06:00];

	K	α	c	p	d	q
β	0.09	-0.12	-0.02	0.08	-0.02	0.00
K		-0.01	0.00	-0.10	-0.06	-0.10
α			0.11	-0.18	0.01	-0.16
c				0.29	-0.04	-0.03
p					-0.08	0.03
d						0.62

Correlation Matrix, [T_{start}=13/11-06:00, T_{end}=14/11-06:00];

	K	α	c	p	d	q
β	-0.04	0.06	-0.07	-0.04	0.04	0.00
K		0.03	0.00	-0.12	-0.11	-0.14
α			0.09	-0.05	-0.04	-0.17
c				0.45	-0.05	-0.08
p					-0.01	-0.07
d						0.73

Correlation Matrix, [T_{start}=14/11-06:00, T_{end}=15/11-06:00];

	K	α	c	p	d	q
β	0.00	0.01	-0.06	-0.07	-0.08	-0.06
K		0.04	-0.10	-0.14	-0.08	-0.08
α			0.09	-0.07	-0.09	-0.18
c				0.68	-0.04	-0.04
p					0.03	0.01
d						0.82

Correlation Matrix, [T_{start}=15/11-06:00, T_{end}=16/11-06:00];

	K	α	c	p	d	q
β	-0.03	0.03	0.00	0.00	0.00	0.02
K		0.01	-0.14	-0.24	-0.10	-0.10
α			0.12	-0.04	-0.14	-0.17
c				0.68	-0.12	-0.13
p					-0.01	-0.01
d						0.83

SI-12. Posterior distribution of the ETAS model parameters based on *Semi-Fast* Method together with the correlation structure

Table SI-4: Distribution of the ETAS model parameters based on *Semi-Fast* method including marginal PDFs of posterior (shown with bar plots) and lognormal PDFs of prior (shown with dashed orange lines), and the statistics of posterior (including mean and [2%-98%] confidence interval (CI) for providing early seismicity forecasts after M_w7.3 at 12-November 2017

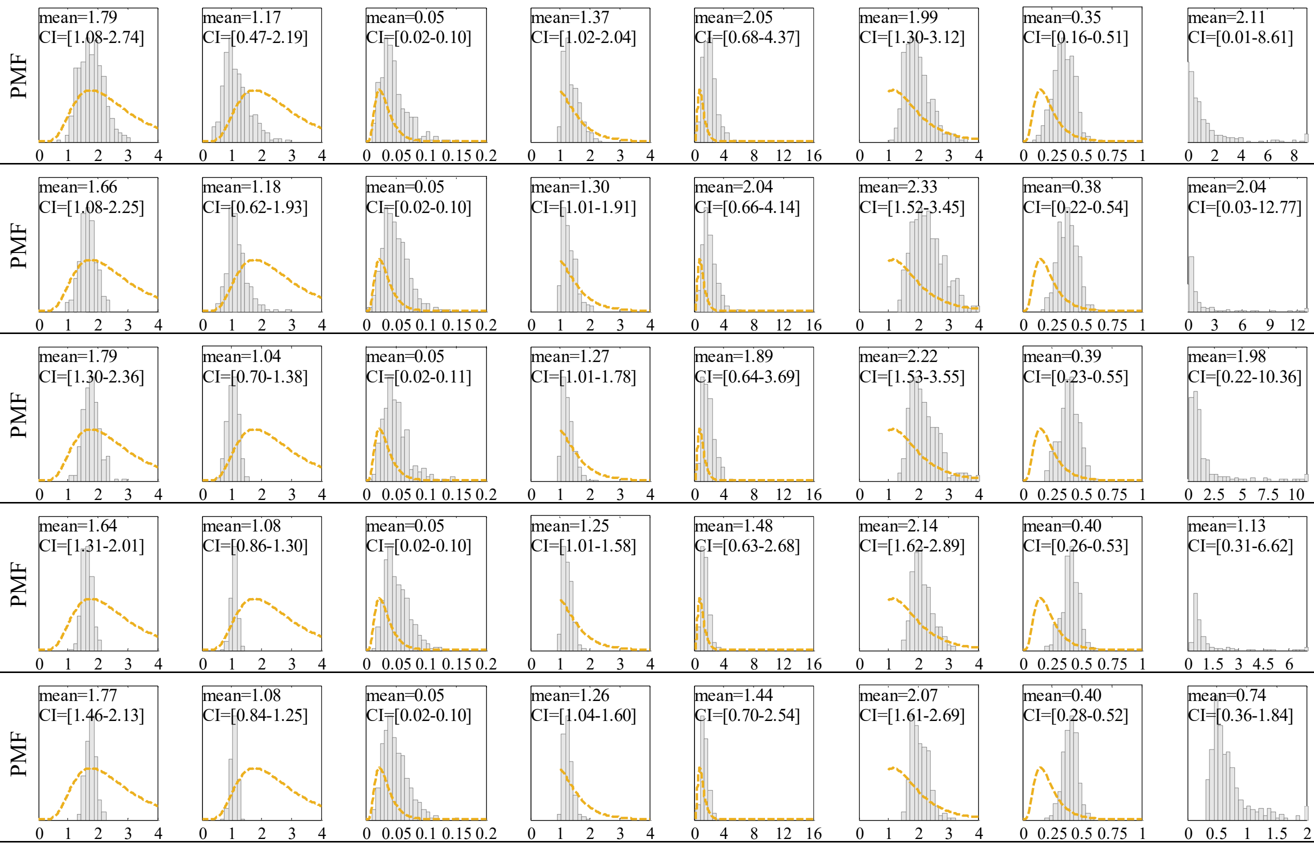

$[T_{start}, T_{end}]^*$ (dd/mm-hour)	β	α	c [day]	p	d [km]	q	γ	K
(a) [12/11-21:00, 13/11-06:00]; $N_d = 372$ $M_l = 3.40$	mean=1.79 CI=[1.08-2.74]	mean=1.17 CI=[0.47-2.19]	mean=0.05 CI=[0.02-0.10]	mean=1.37 CI=[1.02-2.04]	mean=2.05 CI=[0.68-4.37]	mean=1.99 CI=[1.30-3.12]	mean=0.35 CI=[0.16-0.51]	mean=2.11 CI=[0.01-8.61]
(b) [13/11-00:00, 13/11-06:00]; $N_d = 346$ $M_l = 3.30$	mean=1.66 CI=[1.08-2.25]	mean=1.18 CI=[0.62-1.93]	mean=0.05 CI=[0.02-0.10]	mean=1.30 CI=[1.01-1.91]	mean=2.04 CI=[0.66-4.14]	mean=2.33 CI=[1.52-3.45]	mean=0.38 CI=[0.22-0.54]	mean=2.04 CI=[0.03-12.77]
(c) [13/11-06:00, 14/11-06:00]; $N_d = 276$ $M_l = 3.30$	mean=1.79 CI=[1.30-2.36]	mean=1.04 CI=[0.70-1.38]	mean=0.05 CI=[0.02-0.11]	mean=1.27 CI=[1.01-1.78]	mean=1.89 CI=[0.64-3.69]	mean=2.22 CI=[1.53-3.55]	mean=0.39 CI=[0.23-0.55]	mean=1.98 CI=[0.22-10.36]
(d) [14/11-06:00, 15/11-06:00]; $N_d = 376$ $M_l = 3.00$	mean=1.64 CI=[1.31-2.01]	mean=1.08 CI=[0.86-1.30]	mean=0.05 CI=[0.02-0.10]	mean=1.25 CI=[1.01-1.58]	mean=1.48 CI=[0.63-2.68]	mean=2.14 CI=[1.62-2.89]	mean=0.40 CI=[0.26-0.53]	mean=1.13 CI=[0.31-6.62]
(e) [15/11-06:00, 16/11-06:00]; $N_d = 337$ $M_l = 3.00$	mean=1.77 CI=[1.46-2.13]	mean=1.08 CI=[0.84-1.25]	mean=0.05 CI=[0.02-0.10]	mean=1.26 CI=[1.04-1.60]	mean=1.44 CI=[0.70-2.54]	mean=2.07 CI=[1.61-2.69]	mean=0.40 CI=[0.28-0.52]	mean=0.74 CI=[0.36-1.84]
Statistics of prior marginal PDFs	median=2.30 COV=0.50	median=2.30 COV=0.50	median=0.03 COV=0.50	median=1.10 COV=0.50	median=1.00 COV=0.50	median=1.50 COV=0.50	median=0.20 COV=0.50	–

*The time window denotes the *forecasting interval*; note that "hour" is based on UTC.

Correlation Matrix, [T_{start}=12/11-21:00, T_{end}=13/11-06:00];

	K	α	c	p	d	q	γ
β	0.08	-0.02	0.01	0.02	-0.04	0.07	0.08
K		-0.03	0.01	-0.14	0.00	-0.08	-0.09
α			0.06	-0.08	-0.12	0.26	0.21
c				0.11	-0.14	-0.07	0.06
p					-0.08	-0.17	-0.03
d						0.08	-0.66
q							0.49

Correlation Matrix, [T_{start}=13/11-00:00, T_{end}=13/11-06:00];

	K	α	c	p	d	q	γ
β	-0.06	0.07	0.00	-0.01	0.06	0.00	-0.13
K		-0.13	-0.07	-0.24	-0.01	-0.06	-0.03
α			0.23	-0.09	-0.11	0.28	0.16
c				0.23	-0.13	0.09	0.14
p					0.01	-0.08	0.01
d						0.02	-0.76
q							0.46

Correlation Matrix, [T_{start}=13/11-06:00, T_{end}=14/11-06:00];

	K	α	c	p	d	q	γ
β	0.03	-0.01	0.01	0.07	0.03	0.10	0.00
K		-0.09	-0.13	-0.24	0.11	-0.03	-0.17
α			0.05	-0.08	-0.22	0.23	0.24
c				0.40	-0.05	-0.08	-0.05
p					0.02	-0.09	-0.05
d						0.08	-0.73
q							0.45

Correlation Matrix, [T_{start}=14/11-06:00, T_{end}=15/11-06:00];

	K	α	c	p	d	q	γ
β	0.12	-0.01	0.07	-0.04	0.06	0.00	-0.03
K		-0.09	-0.23	-0.42	0.03	-0.03	-0.04
α			0.10	-0.11	-0.23	0.25	0.27
c				0.64	-0.09	-0.12	0.01
p					0.00	-0.18	-0.04
d						0.12	-0.76
q							0.44

Correlation Matrix, [T_{start}=15/11-06:00, T_{end}=16/11-06:00];

	K	α	c	p	d	q	γ
β	-0.05	-0.09	-0.07	0.00	-0.05	-0.12	-0.02
K		-0.02	-0.18	-0.40	-0.07	0.05	0.06
α			0.04	-0.25	-0.21	0.21	0.25
c				0.67	0.00	0.08	0.01
p					0.18	-0.11	-0.19
d						0.18	-0.74
q							0.40

SI-13. Posterior distribution of the ETAS model parameters based on *Fast* Method together with the correlation structure

Table SI-5: Distribution of the ETAS model parameters based on *Fast* method including marginal PDFs of posterior (shown with bar plots) and lognormal PDFs of prior (shown with dashed orange lines), and the statistics of posterior (including mean and [2%-98%] confidence interval (CI) for providing early seismicity forecasts after M_w7.3 at 12-November 2017

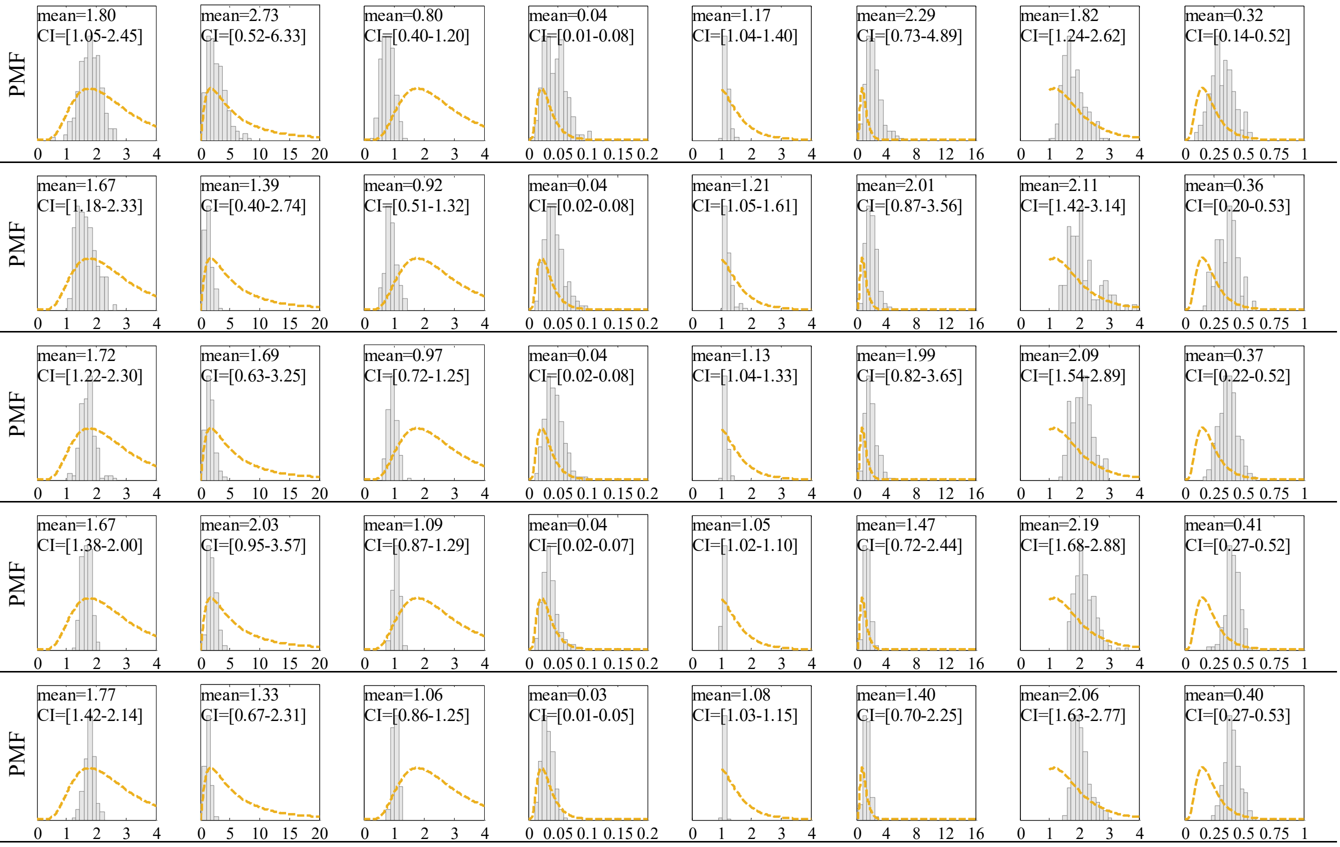

$[T_{start}, T_{end}]$* (dd/mm-hour)	β	K	α	c [day]	p	d [km]	q	γ
(a) [12/11-21:00, 13/11-06:00]; $N_d = 231$ $M_l = 3.40$	mean=1.80 CI=[1.05-2.45]	mean=2.73 CI=[0.52-6.33]	mean=0.80 CI=[0.40-1.20]	mean=0.04 CI=[0.01-0.08]	mean=1.17 CI=[1.04-1.40]	mean=2.29 CI=[0.73-4.89]	mean=1.82 CI=[1.24-2.62]	mean=0.32 CI=[0.14-0.52]
(b) [13/11-00:00, 13/11-06:00]; $N_d = 118$ $M_l = 3.30$	mean=1.67 CI=[1.18-2.33]	mean=1.39 CI=[0.40-2.74]	mean=0.92 CI=[0.51-1.32]	mean=0.04 CI=[0.02-0.08]	mean=1.21 CI=[1.05-1.61]	mean=2.01 CI=[0.87-3.56]	mean=2.11 CI=[1.42-3.14]	mean=0.36 CI=[0.20-0.53]
(c) [13/11-06:00, 14/11-06:00]; $N_d = 171$ $M_l = 3.30$	mean=1.72 CI=[1.22-2.30]	mean=1.69 CI=[0.63-3.25]	mean=0.97 CI=[0.72-1.25]	mean=0.04 CI=[0.02-0.08]	mean=1.13 CI=[1.04-1.33]	mean=1.99 CI=[0.82-3.65]	mean=2.09 CI=[1.54-2.89]	mean=0.37 CI=[0.22-0.52]
(d) [14/11-06:00, 15/11-06:00]; $N_d = 236$ $M_l = 3.00$	mean=1.67 CI=[1.38-2.00]	mean=2.03 CI=[0.95-3.57]	mean=1.09 CI=[0.87-1.29]	mean=0.04 CI=[0.02-0.07]	mean=1.05 CI=[1.02-1.10]	mean=1.47 CI=[0.72-2.44]	mean=2.19 CI=[1.68-2.88]	mean=0.41 CI=[0.27-0.52]
(e) [15/11-06:00, 16/11-06:00]; $N_d = 222$ $M_l = 3.00$	mean=1.77 CI=[1.42-2.14]	mean=1.33 CI=[0.67-2.31]	mean=1.06 CI=[0.86-1.25]	mean=0.03 CI=[0.01-0.05]	mean=1.08 CI=[1.03-1.15]	mean=1.40 CI=[0.70-2.25]	mean=2.06 CI=[1.63-2.77]	mean=0.40 CI=[0.27-0.53]
Statistics of prior marginal PDFs	median=2.30 COV=0.50	median=5.00 COV=1.00	median=2.30 COV=0.50	median=0.03 COV=0.50	median=1.10 COV=0.50	median=1.00 COV=0.50	median=1.50 COV=0.50	median=0.20 COV=0.50

*The time window denotes the *forecasting interval*; note that "hour" is based on UTC.

Correlation Matrix, [T_{start}=12/11-21:00, T_{end}=13/11-06:00];

	K	α	c	p	d	q	γ
β	0.06	0.01	0.04	-0.05	-0.09	-0.07	0.04
K		-0.39	0.00	-0.54	0.01	0.02	0.10
α			0.32	-0.25	-0.20	0.25	0.24
c				-0.00	-0.24	0.01	0.25
p					0.13	-0.23	-0.25
d						0.14	-0.61
q							0.52

Correlation Matrix, [T_{start}=13/11-00:00, T_{end}=13/11-06:00];

	K	α	c	p	d	q	γ
β	0.02	-0.09	-0.06	0.00	0.05	-0.04	-0.10
K		-0.40	0.05	-0.56	-0.03	-0.09	-0.08
α			0.07	-0.30	-0.34	0.34	0.33
c				0.13	-0.09	0.11	0.07
p					0.27	-0.14	-0.15
d						-0.15	-0.70
q							0.64

Correlation Matrix, [T_{start}=13/11-06:00, T_{end}=14/11-06:00];

	K	α	c	p	d	q	γ
β	0.09	-0.04	-0.02	-0.04	0.00	0.12	0.06
K		-0.36	0.01	-0.62	0.03	-0.02	0.02
α			0.09	-0.24	-0.21	0.22	0.27
c				0.25	0.04	0.05	-0.05
p					0.06	-0.13	-0.15
d						-0.01	-0.74
q							0.56

Correlation Matrix, [T_{start}=14/11-06:00, T_{end}=15/11-06:00];

	K	α	c	p	d	q	γ
β	-0.03	0.11	0.08	-0.07	-0.08	-0.03	0.07
K		-0.45	-0.01	-0.67	0.08	-0.05	-0.03
α			0.16	-0.20	-0.28	0.08	0.17
c				0.15	-0.02	0.15	0.10
p					0.13	0.03	-0.07
d						0.18	-0.70
q							0.46

Correlation Matrix, [T_{start}=15/11-06:00, T_{end}=16/11-06:00];

	K	α	c	p	d	q	γ
β	-0.10	-0.11	0.10	0.18	0.08	0.13	0.01
K		-0.28	-0.07	-0.76	0.00	-0.08	-0.02
α			0.06	-0.22	-0.28	0.06	0.20
c				0.23	-0.01	-0.01	-0.03
p					0.16	0.12	-0.05
d						0.15	-0.71
q							0.48
